# Magnetic Resonance Imaging, Clinical, and Biopsy Findings in Suspected Prostate Cancer: A Systematic Review and Meta-Analysis

**DOI:** 10.1001/jamanetworkopen.2024.4258

**Published:** 2024-03-29

**Authors:** Arya Haj-Mirzaian, Kristine S. Burk, Ronilda Lacson, Daniel I. Glazer, Sanjay Saini, Adam S. Kibel, Ramin Khorasani

**Affiliations:** 1 Center for Evidence-Based Imaging, Department of Radiology, Brigham and Women’s Hospital, Harvard Medical School, Boston, Massachusetts; 2 Department of Radiology, Brigham and Women’s Hospital, Harvard Medical School, Boston, Massachusetts; 3 Dana-Farber Cancer Institute, Harvard Medical School, Boston, Massachusetts; 4 Department of Radiology, Massachusetts General Hospital, Harvard Medical School, Boston, Massachusetts; 5 Division of Urological Surgery, Brigham and Women’s Hospital, Harvard Medical School, Boston, Massachusetts

## Abstract

**Importance:**

Multiple strategies integrating magnetic resonance imaging (MRI) and clinical data have been proposed to determine the need for a prostate biopsy in men with suspected clinically significant prostate cancer (csPCa) (Gleason score ≥3 + 4). However, inconsistencies across different strategies create challenges for drawing a definitive conclusion.

**Objective:**

To determine the optimal prostate biopsy decision-making strategy for avoiding unnecessary biopsies and minimizing the risk of missing csPCa by combining MRI Prostate Imaging Reporting & Data System (PI-RADS) and clinical data.

**Data Sources:**

PubMed, Ovid MEDLINE, Embase, Web of Science, and Cochrane Library from inception to July 1, 2022.

**Study Selection:**

English-language studies that evaluated men with suspected but not confirmed csPCa who underwent MRI PI-RADS followed by prostate biopsy were included. Each study had proposed a biopsy plan by combining PI-RADS and clinical data.

**Data Extraction and Synthesis:**

Studies were independently assessed for eligibility for inclusion. Quality of studies was appraised using the Quality Assessment of Diagnostic Accuracy Studies 2 tool and the Newcastle-Ottawa Scale. Mixed-effects meta-analyses and meta-regression models with multimodel inference were performed. Reporting of this study followed the Preferred Reporting Items for Systematic Reviews and Meta-Analyses (PRISMA) guideline.

**Main Outcomes and Measures:**

Independent risk factors of csPCa were determined by performing meta-regression between the rate of csPCa and PI-RADS and clinical parameters. Yields of different biopsy strategies were assessed by performing diagnostic meta-analysis.

**Results:**

The analyses included 72 studies comprising 36 366 patients. Univariable meta-regression showed that PI-RADS 4 (β-coefficient [SE], 7.82 [3.85]; *P* = .045) and PI-RADS 5 (β-coefficient [SE], 23.18 [4.46]; *P* < .001) lesions, but not PI-RADS 3 lesions (β-coefficient [SE], −4.08 [3.06]; *P* = .19), were significantly associated with a higher risk of csPCa. When considered jointly in a multivariable model, prostate-specific antigen density (PSAD) was the only clinical variable significantly associated with csPCa (β-coefficient [SE], 15.50 [5.14]; *P* < .001) besides PI-RADS 5 (β-coefficient [SE], 9.19 [3.33]; *P* < .001). Avoiding biopsy in patients with lesions with PI-RADS category of 3 or less and PSAD less than 0.10 (vs <0.15) ng/mL^2^ resulted in reducing 30% (vs 48%) of unnecessary biopsies (compared with performing biopsy in all suspected patients), with an estimated sensitivity of 97% (vs 95%) and number needed to harm of 17 (vs 15).

**Conclusions and Relevance:**

These findings suggest that in patients with suspected csPCa, patient-tailored prostate biopsy decisions based on PI-RADS and PSAD could prevent unnecessary procedures while maintaining high sensitivity.

## Key Points

**Question:**

What is the optimal approach to integrating prostate magnetic resonance imaging and clinical parameters for identifying patients requiring prostate biopsy while avoiding unnecessary procedures and minimizing the risk of missing clinically significant prostate cancer (csPCa)?

**Findings:**

In this systematic review and meta-analysis of 36 366 patients, Prostate Imaging Reporting & Data System (PI-RADS) category 4 and 5 lesions and prostate-specific antigen density (PSAD) were the only independent imaging and clinical factors associated with csPCa. The strategy to forego biopsy in men lesions with PI-RADS category of 3 or less and PSAD less than 0.10 or less than 0.15 ng/mL^2^ could reduce unnecessary biopsies by 30% or 48%, respectively, while maintaining a sensitivity of 97% or 95%.

**Meaning:**

These findings suggest that prostate biopsies may not be necessary for patients with equivocal or negative magnetic resonance imaging results and low PSAD.

## Teaser

This systematic review and meta-analysis evaluates biopsy decision-making strategies for avoiding unnecessary biopsies and minimizing risk of missing clinically significant prostate cancer.

## Introduction

Prostate cancer is the second most common cancer in men worldwide, with an estimated incidence of 1.4 million in 2020.^1^ Several guidelines recommend magnetic resonance imaging (MRI) as a tool to identify clinically significant prostate cancer (csPCa) in all individuals with suspected prostate cancer.^2,3,4^ Thereby, biparametric or multiparametric MRI is routinely performed in accordance with the Prostate Imaging Reporting & Data System (PI-RADS) in men with suspected csPCa, including biopsy-naive patients or patients with previously negative biopsy results.^5^ Patients with focal lesions scored as PI-RADS category 4 or 5 are considered to have a high likelihood of csPCa and should undergo an image-guided targeted biopsy.^6^ However, the published literature regarding the association of PI-RADS 3 or less lesions and csPCa is controversial, and there is no consensus on which patients with equivocal (PI-RADS category 3) or negative (PI-RADS categories 1, 2, or no focal lesion) prostate MRI findings could avoid biopsy.^7^

At most institutions, men with suspected csPCa and negative or equivocal MRI results are still referred for systematic prostate biopsy due to the limitation of MRI PI-RADS in excluding csPCa with an overall sensitivity of 85%, consistent with multiple guidelines such as the American Urological Association, European Association of Urology, European Society for Radiotherapy and Oncology, and National Comprehensive Cancer Network.^2,3,4,8,9^ The major challenge of this approach is the low yield of systematic biopsy in this patient cohort and its associated morbidity and health care costs. As such, ancillary clinical data have been proposed to complement MRI to minimize the number of unnecessary biopsies. This concept has been recently expanded by numerous studies, and several MRI-based risk models and strategies have been developed to guide decisions on prostate biopsy.^10,11,12,13,14,15,16,17,18,19,20,21,22,23,24,25,26,27,28,29,30,31,32,33,34,35,36,37,38,39,40,41,42,43,44,45,46,47,48,49,50,51,52,53,54,55,56,57,58,59,60,61,62,63,64,65,66,67,68,69,70,71,72,73,74,75,76,77,78,79,80,81^ However, it has been challenging to integrate the proposed approaches into clinical practice owing to considerable inconsistencies among them. Previously performed studies were predominantly single institutional, enrolled heterogeneous patient populations, and incorporated different sets of clinical parameters with PI-RADS. The ability to extrapolate broader conclusions from these studies is therefore limited. The purpose of this study was to determine optimal prostate biopsy decision-making by combining MRI PI-RADS with clinical data to avoid unnecessary prostate biopsies while minimizing the risk of missed csPCa.

## Methods

### Design

This systematic review and meta-analysis was performed and reported in accordance with the Cochrane Handbook for Systematic Reviews of Interventions^82^ and the Preferred Reporting Items for Systematic Reviews and Meta-Analyses (PRISMA) guidelines for searching, diagnostic test accuracy, and harms outcomes.^83^

### Literature Search and Studies Selection

A comprehensive search was conducted in PubMed, Ovid MEDLINE, Web of Science, Embase, and the Cochrane Library from inception to July 1, 2022. Bibliographies of the relevant review articles were manually examined for possible inclusion of additional eligible studies. Search terms are presented in eMethods 1 in Supplement 1.

Eligibility criteria were defined based on the population, intervention, comparison, and outcome approach. Studies were considered eligible for inclusion when meeting all of the following criteria: (1) included patients with suspected csPCa, but not patients on active surveillance or patients with previously confirmed or treated prostate cancer; (2) all patients underwent prebiopsy prostate MRI with PI-RADS assessment; (3) all patients had at least 1 relevant clinical parameter available, such as prebiopsy prostate-specific antigen (PSA); (4) all patients underwent systematic and targeted (for PI-RADS≥3 lesions) transrectal and/or transperineal prostate biopsy^12,84^; (5) all patients had csPCa defined based on the International Society of Urological Pathology guideline (ie, Gleason score ≥3 + 4)^85^; and (6) the study combined PI-RADS and clinical parameters (eg, PSA) to propose a biopsy decision plan. Exclusion criteria included studies (1) involving nonhuman subjects, (2) not published in English, or (3) not published as an original article. Eligibility criteria details are presented in eMethods 2 in Supplement 1.

After removing duplicates, 2 reviewers (A.H.M., a third-year radiology resident, and K.S.B., a fellowship-trained abdominal radiologist with 3 years of experience) independently screened all titles and abstracts in duplicate using Covidence software.^86^ The full text of articles that passed initial screening was examined by the same reviewers using a predefined stepwise protocol (eMethods 3 in Supplement 1). Disagreements were resolved by consensus. The interrater agreement between reviewers for the binary decision of inclusion or exclusion was assessed using 200 randomly selected abstracts and showed a strong level of agreement with a Cohen κ of 0.93.

### Data Extraction and Quality Assessment

Data extraction and quality assessment were conducted by a reviewer (A.H.M.) using standardized extraction forms. Data regarding study design (author, year of publication, number of patients, prospective vs retrospective, consecutive vs nonconsecutive, and inclusion and exclusion criteria), patient characteristics (age, body mass index, race and ethnicity according to their respective study definitions [included because of their potential association with the rate of csPCa], family history of PCa, positive digital rectal examination findings, and prior prostate biopsy), prostate MRI (Tesla, multiparametric vs biparametric), PI-RADS, prostate volume, transitional vs peripheral zone index lesion, clinical parameters (total PSA, free PSA, free/total PSA, and PSA density [PSAD]), biopsy (biopsy method, pathology assessment method, and time interval between MRI and biopsy), and rate of csPCa were recorded.

The quality assessment was conducted using the Quality Assessment of Diagnostic Accuracy Studies 2 tool and the Newcastle-Ottawa Scale.^87,88^ Both tools were modified in accordance with the research question. By combining these tools, studies were rated as having a low, moderate, or high risk of bias. Further details regarding the quality assessment are presented in eMethods 4 and 5, eTables 1 and 2, and eFigure 1 in Supplement 1.

### Statistical Analysis

The meta-analysis was performed using R Studio, version 1.1.383 (R Project for Statistical Computing) using the meta, version 4.13-0 and metafor, version 2.4-0 packages. The pooled logit-transformed proportions of csPCa were calculated using a random-effects approach and generalized linear mixed-effects model via the metaprop function.^89,90^ Between-study heterogeneity was estimated using *I*^2^ values with cutoffs of 25%, 50%, and 75% to distinguish low, moderate, and high heterogeneity, respectively.^91^ Potential publication bias was assessed using a funnel plot and Egger regression asymmetry test using the metabias function.^90,92^

Univariable mixed-effects meta-regression was used to assess independent risk factors of csPCa using the metareg function. Multicollinearity of the factors associated with csPCa was evaluated using an intercorrelation matrix and addressed by combining and/or removing close-to-redundant factors with an absolute *r* greater than 0.6.^93^ Multivariable mixed-effects meta-regression was then performed using the multimodel inference that allows examination of all possible combinations of risk factors and definition of the most important set of variables associated with csPCa (eMethods 6 in Supplement 1).^94^

Furthermore, we selected studies that assessed the yield of combining PI-RADS and PSAD by reporting patient-level data. The pooled sensitivity, negative predictive value (NPV), number needed to harm (NNH) for not performing a biopsy, and percentage of patients avoiding unnecessary biopsy were calculated using a generalized linear mixed-effects model.^89,90^ Unnecessary biopsy was defined as performing a biopsy in a patient without csPCa in retrospect. A 2-tailed *P* < .05 was considered significant.

## Results

### Study and Population Characteristics

**Figure 1.**
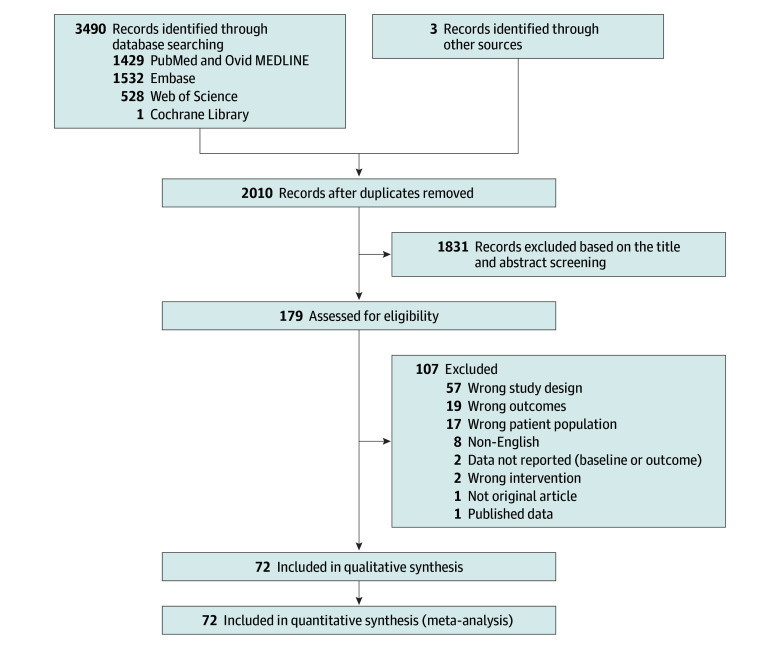
Search Methods and Screening Process

The median patient age was 65.6 years (range, 61.3-69.3 years). Black race was reported for a median of 14% (range, 1%-29%) of all patients included in the analysis. Median total PSA and PSAD of the patients in the included studies were 7.8 ng/mL (range, 5.1-14.7 ng/mL) and 0.15 ng/mL^2^ (range, 0.10-0.33 ng/mL^2^), respectively (to convert PSA levels to μg/L, multiply by 1.0). Most of the included patients were biopsy-naive (81%); the rest had a prior negative biopsy (18%) or prior nonsignificant Gleason score 3 + 3 PCa (<1%). A total of 72 studies including 36 366 patients with suspected csPCa who underwent prostate MRI and subsequent biopsy were included (Figure 1).^10,11,12,13,14,15,16,17,18,19,20,21,22,23,24,25,26,27,28,29,30,31,32,33,34,35,36,37,38,39,40,41,42,43,44,45,46,47,48,49,50,51,52,53,54,55,56,57,58,59,60,61,62,63,64,65,66,67,68,69,70,71,72,73,74,75,76,77,78,79,80,81^ Details of the included study and patient characteristics are summarized in Table 1 and eTables 3 to 5 in Supplement 1. Studies were published between 2016 and 2022. None of the included studies enrolled patients with prior csPCa. Among the 72 included studies, 19 solely included patients with low to moderate risk of csPCa (ie, PI-RADS≤3 and/or transitional zone index lesion and/or total PSA <10 ng/mL),^12,17,23,24,28,29,30,39,49,50,51,57,59,60,62,63,68,70,76^ 1 study solely included high-risk patients with PI-RADS 4 or greater,^20^ and the remaining 52 studies included all suspected patients regardless of the prostate MRI results or clinical parameters.^10,11,13,14,15,16,18,19,21,22,25,26,27,31,32,33,34,35,36,37,38,40,41,42,43,44,45,46,47,48,52,53,54,55,56,58,61,64,65,66,67,69,71,72,73,74,75,77,78,79,80,81^ The most frequently used imaging was 3.0 T (92.6%) and multiparametric MRI (80.3%). Pooled percentages of included patients indicated that a median 27% (range, 0%-100%), 21% (range, 0%-100%), and 48% (range, 0%-100%) had a PI-RADS of 2 or less (including no focal lesion), PI-RADS 3, and PI-RADS 4 or more index lesion, respectively. Systematic and targeted (for PI-RADS≥3 index lesions) transrectal or transperineal prostate biopsy was performed in all included patients. Rates of csPCa ranged from 5% to 80% with a median of 35%. The median rate of nonsignificant PCa was 15% (range, 3%-33%).

**Table 1.** Patient and Study Characteristics

Characteristic	No. (%) or median (range)^a^
Total No. of patients (72 studies^10,11,12,13,14,15,16,17,18,19,20,21,22,23,24,25,26,27,28,29,30,31,32,33,34,35,36,37,38,39,40,41,42,43,44,45,46,47,48,49,50,51,52,53,54,55,56,57,58,59,60,61,62,63,64,65,66,67,68,69,70,71,72,73,74,75,76,77,78,79,80,81^)	36 366
No. of patients per study	351 (52-2512)
Study design	
Prospective	31 (43.1)
Consecutive enrollment	32 (44.4)
Not reported	9 (12.5)
Clinical parameters	
Age (70 studies,^10,11,12,13,14,15,16,17,18,19,20,21,23,24,25,26,27,28,29,30,31,32,33,34,35,36,37,38,39,40,41,42,43,44,45,46,47,48,49,50,51,52,53,54,55,56,57,58,60,61,62,63,64,65,66,67,68,69,70,71,72,73,74,75,76,77,78,79,80,81^ 35 949 patients), y	65.6 (61.3-69.3)
Total PSA (71 studies,^10,11,12,13,14,15,16,17,18,19,20,21,22,23,24,25,26,27,28,29,30,31,32,33,34,35,36,37,38,39,40,41,42,43,44,45,46,47,48,49,50,51,52,53,54,55,56,57,58,60,61,62,63,64,65,66,67,68,69,70,71,72,73,74,75,76,77,78,79,80,81^ 36 211 patients), ng/mL	7.8 (5.1-14.7)
Free PSA (7 studies,^12,32,60,63,69,72,76^ 2348 patients), ng/mL	1.3 (1.1-13.0)
Free/total PSA (15 studies,^10,16,18,28,33,36,49,60,61,63,68,69,72,75,76^ 6038 patients), %	15 (13-19)
PSAD (54 studies,^11,13,15,16,17,18,19,20,21,23,25,26,27,28,29,30,32,35,36,37,38,40,41,42,43,45,46,47,49,50,51,54,55,57,58,60,61,62,63,64,65,67,68,69,70,71,72,73,74,75,76,77,78,80^ 32 200 patients), ng/mL^2^	0.15 (0.10-0.33)
BMI (9 studies,^13,17,19,24,25,31,39,49,79^ 3946 patients)	24.7 (24.2-30.8)
Positive family history of PCa (16 studies,^12,24,26,38,44,46,49,52,56,58,62,63,64,72,73,74^ 10 095 patients), %	17 (1-35)
Positive DRE (33 studies,^10,15,17,18,21,22,23,26,31,32,33,37,38,41,42,44,45,46,47,51,52,53,56,58,62,63,64,72,73,74,77,78,81^ 19 129 patients), %	23 (7-68)
Black race (5 studies,^12,15,46,66,79^ 1880 patients), %	14 (1-29)
Prior negative biopsy (58 studies,^10,11,12,13,15,17,18,21,22,23,24,25,26,28,29,31,33,34,35,37,38,39,40,41,42,43,44,45,46,47,48,49,50,51,52,53,54,55,56,57,58,60,61,62,63,64,65,66,67,70,71,72,73,74,76,77,78,79^ 31 177 patients), %	18 (0-100)
Biopsy naive (59 studies,^10,11,12,13,14,17,18,21,22,23,24,25,26,28,29,31,32,33,34,35,37,38,39,40,41,42,43,45,46,47,48,49,50,51,52,53,54,55,56,57,58,59,60,61,62,63,65,67,70,71,72,73,74,76,77,78,79,80,81^ 28 956 patients), %	81 (0-100)
Prior nonsignificant Gleason score 3 + 3 PCa (58 studies,^10,11,12,13,15,17,18,21,22,23,24,25,26,28,29,31,33,34,35,37,38,39,40,41,42,43,45,47,48,49,50,51,52,53,54,55,56,57,58,59,60,61,62,63,64,65,66,67,69,70,71,72,73,74,76,77,78,79^ 30 620 patients), %	0 (0-32)
MRI	
Tesla (68 studies,^10,11,12,13,14,15,16,17,18,19,21,22,23,24,25,26,27,28,29,30,31,32,33,34,35,36,37,38,39,40,41,42,43,44,45,46,47,48,49,50,51,53,54,55,56,57,58,59,60,61,62,63,64,65,67,68,69,70,71,72,74,75,76,77,78,79,80,81^ 34 351 patients)^b^	
1.5 T	19 (27.9)
3.0 T	63 (92.6)
Sequence (71 studies,^10,11,12,13,14,15,16,17,18,19,20,21,22,23,24,25,26,27,28,29,30,31,32,33,34,35,36,37,38,39,40,41,42,43,44,45,46,47,48,49,50,51,52,53,54,55,56,57,58,59,60,61,63,64,65,66,67,68,69,70,71^ 35 902 patients)^b^	
Biparametric	16 (22.5)
Multiparametric	57 (80.3)
Prostate volume (58 studies,^10,11,12,15,16,17,18,20,21,23,24,25,26,27,28,29,31,32,34,35,37,38,39,40,41,42,43,44,45,46,47,48,50,51,52,53,54,55,57,58,60,61,62,63,65,66,68,69,70,71,72,73,74,75,76,78,79,80^ 29 961 patients), mL	50.5 (28.7-66.0)
PI-RADS, index lesion (72 studies,^10,11,12,13,14,15,16,17,18,19,20,21,22,23,24,25,26,27,28,29,30,31,32,33,34,35,36,37,38,39,40,41,42,43,44,45,46,47,48,49,50,51,52,53,54,55,56,57,58,59,60,61,62,63,64,65,66,67,68,69,70,71,72,73,74,75,76,77,78,79,80,81^ 36 366 patients), %^c^	
No focal lesion, 1, or 2	27 (0-100)
3	21 (0-100)
4	30 (0-58)
5	18 (0-42)
Location of index lesion (8 studies,^11,20,25,29,39,50,75,76^ 3549 patients), %^c^	
Peripheral zone	44 (0-65)
Transitional zone	54 (19-100)
Biopsy	
csPCa (72 studies,^10,11,12,13,14,15,16,17,18,19,20,21,22,23,24,25,26,27,28,29,30,31,32,33,34,35,36,37,38,39,40,41,42,43,44,45,46,47,48,49,50,51,52,53,54,55,56,57,58,59,60,61,62,63,64,65,66,67,68,69,70,71,72,73,74,75,76,77,78,79,80,81^ 36 366 patients), %	35 (5-80)
Non-csPCa (62 studies,^11,12,14,15,16,17,18,19,21,22,23,24,25,26,27,28,31,32,33,34,35,36,37,39,40,41,42,43,44,45,46,47,51,52,53,54,56,57,58,59,60,61,62,63,64,65,66,67,68,69,70,71,72,73,74,75,76,77,78,79,80,81^ 31 408 patients), %	15 (3-33)

Abbreviations: BMI, body mass index (measured as weight in kilograms divided by height in meters squared); csPCa, clinically significant prostate cancer; DRE, digital rectal examination; MRI, magnetic resonance imaging; PI-RADS, Prostate Imaging Reporting & Data System; PSA, prostate-specific antigen; PSAD, prostate-specific antigen density.

SI conversion factor: To convert PSA levels to μg/L, multiply by 1.0.

^a^ Specified per row. Values in parentheses represent the full range of reported data across studies.

^b^ Some studies used both a 1.5- and 3.0-T scanner and both bi- and multiparametric approaches.

^c^ Pooled percentages were calculated using random- and mixed-effects meta-analysis and may not sum to 1.

### Quality Assessment and Publication Bias

Eight studies (11%) were judged to have a moderate risk of bias.^16,19,20,26,28,30,36,81^ No potential source of bias was identified for the other 64 (89%) studies.^10,11,12,13,14,15,17,18,21,22,23,24,25,27,29,31,32,33,34,35,37,38,39,40,41,42,43,44,45,46,47,48,49,50,51,52,53,54,55,56,57,58,59,60,61,62,63,64,65,66,67,68,69,70,71,72,73,74,75,76,77,78,79,80^ Retrospective study design and concern regarding the selection domain (ie, studies only noted that patients with suspected csPCa were included rather than providing detailed inclusion and/or exclusion criteria) were the main sources of bias. Details of the quality assessment are presented in eTables 1 and 2 and eFigure 1 in Supplement 1. A funnel plot in which the *P* value of the weighted linear regression test was 0.65 demonstrated the absence of publication bias (eFigure 2 in Supplement 1).

### Meta-Regression: Independent Determinants of csPCa

**Figure 2.**
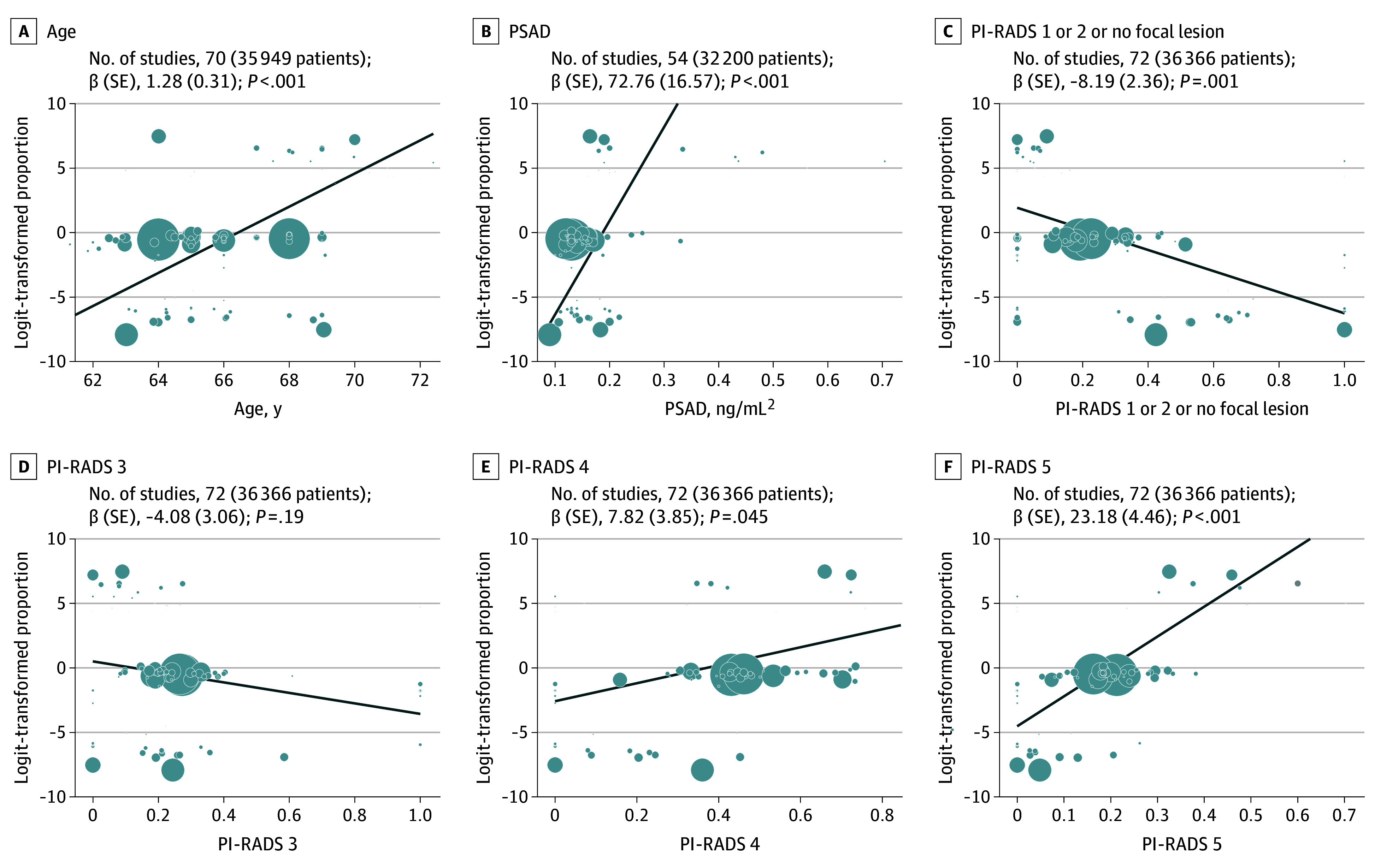
Univariable Meta-Regression for the Association Between the Rate of Clinically Significant Prostate Cancer (csPCa) and Imaging and Clinical Parameters Meta-regression of the proportion of csPCa vs mean and median of parameters on a study-level basis. Values on the y-axes represent the logit-transformed rate of csPCa effect based on a random-effects model. *P* values were obtained from a univariable linear mixed-effects model. PI-RADS indicates Prostate Imaging Reporting & Data System; PSAD, prostate-specific antigen density.

On univariable meta-regression, the following continuous or categorical variables were associated with a higher risk of csPCa (Figure 2; eFigures 3-5 in Supplement 1): age, years (β-coefficient [SE], 1.28 [0.31]; *P* < .001); total PSA (β-coefficient [SE], 1.18 [0.26]; *P* < .001); PSAD (β-coefficient [SE], 72.76 [16.57; *P* < .001); PI-RADS 4 (β-coefficient [SE], 7.82 [3.85]; *P* = .045); and PI-RADS 5 (β-coefficient [SE], 23.18 [4.46]; *P* < .001). The following variables were associated with a lower risk of csPCa (Figure 2; eFigures 3-5 in Supplement 1): free/total PSA (β-coefficient [SE], −662.97 [253.14]; *P* = .02); prostate volume (β-coefficient [SE], −0.39 [0.08]; *P* < .001); and PI-RADS 2 or less, including no focal lesion (β-coefficient [SE], −8.19 [2.36]; *P* = .001). However, PI-RADS 3 was not associated with a lower risk of csPCa (β-coefficient [SE], −4.08 [3.06]; *P* = .19).

The intercorrelation matrix showed a significantly high level of correlation among total PSA (|*r*|>0.6; *P* < .001 for the correlation between PSA and PSAD, and |*r*|>0.6; *P* < .001 for the correlation between PSA and free/total PSA), free/total PSA (|*r*|>0.6; *P* < .001 for the correlation between free/total PSA and PSAD), PSAD (|*r*|>0.6; *P* < .001 for the correlation between PSAD and prostate volume), and prostate volume (eFigure 6 in Supplement 1). Moreover, a moderate level of correlation was noted between PI-RADS 2 or less and PI-RADS 4 (|*r*|>0.6; *P* < .001). To avoid multicollinearity, each multivariable model was built based on a distinct set of noncollinear variables (|*r*|<0.6). For instance, PSAD was chosen to use solely in the multivariable models as it contains all components of other clinical parameters, including total PSA and prostate volume.

**Figure 3.**
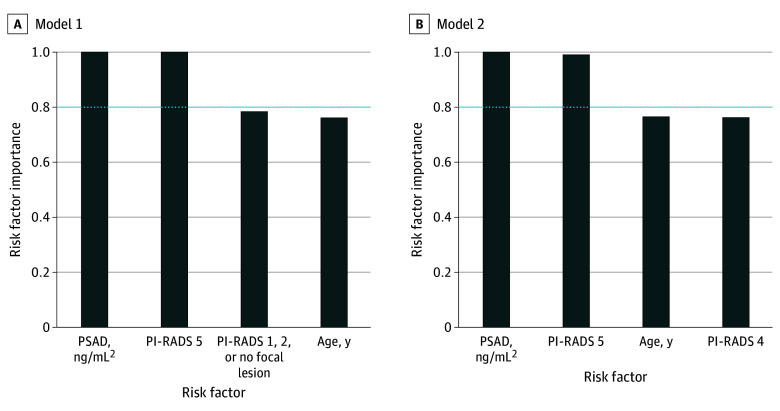
Multivariable Meta-Regression for the Association Between the Rate of Clinically Significant Prostate Cancer (csPCa) and Clinical and Imaging Parameters To avoid multicollinearity, each model was built based on a distinct set of noncollinear variables. Multimodel inference analysis represents models and variables with the highest importance in estimating csPCa. Variables with a risk factor importance of 1 represent the highest significance. The horizontal blue line indicates the cutoff value of 0.8, differentiating between important and less important risk factors; PI-RADS, Prostate Imaging Reporting & Data System; and PSAD, prostate-specific antigen density.

On multivariable meta-regression with multimodel inference, the following variables showed the highest importance with a significant independent association with a higher risk of csPCa (PI-RADS 5: β-coefficient [SE], 9.19 [3.33]; PSAD: β-coefficient [SE], 15.50 [5.14]; both *P* < .001) (Figure 3). Limiting multivariable meta-regression to studies (1) with no risk of bias, (2) including all suspected patients regardless of the prostate MRI results or clinical parameters, and (3) only including biopsy-naive patients showed similar results with the same independent risk factors of csPCa (eTable 6 in Supplement 1).

### Diagnostic Meta-Analysis: Yield of Combining PI-RADS and PSAD

In patients with PI-RADS 2 or less, including no focal lesions (7-11 studies including 1499-2970 patients), avoiding prostate biopsy if PSAD was less than 0.10 ng/mL^2^ (vs <0.15 and <0.20 ng/mL^2^) showed sensitivity of 83% (vs 66% and 36%), NPV of 95% (vs 94% and 92%), NNH of 19 (vs 16 and 13), and avoidance of unnecessary biopsy in 39% (vs 67% and 89%) of men (by comparing performing biopsy in all suspected patients) (Table 2).^17,21,22,23,27,35,41,43,45,55,59,67^ Similarly in patients with PI-RADS of 3 (8-10 studies comprising 1386-1644 patients), avoiding prostate biopsy if PSAD was less than 0.10 ng/mL^2^ (vs <0.15 and <0.20 ng/mL^2^) showed sensitivity of 85% (vs 70% and 44%), NPV of 93% (vs 90% and 87%), NNH of 15 (vs 10 and 8), and avoidance of unnecessary biopsy in 43% (vs 66% and 84%) of men (Table 2).^21,22,25,27,35,41,45,50,51,54,55,56,67^ In patients with PI-RADS 4 or greater (6-7 studies comprising 2716-3114 patients), avoiding prostate biopsy even if PSAD was less than 0.10 ng/mL^2^ (vs <0.15 and <0.20 ng/mL^2^) resulted in an NNH of 3 (vs 2 and 2) (Table 2).^21,22,27,35,41,45,55,67^ Overall, avoiding prostate biopsy in patients with PI-RADS 3 or less and PSAD less than 0.10 ng/mL^2^ (vs <0.15 and <0.20 ng/mL^2^) resulted in avoiding 30% (vs 48% and 59%) of unnecessary biopsies (by comparing performing biopsy in all suspected patients), with an estimated sensitivity of 97% (vs 95% and 87%), NPV of 94% (vs 93% and 90%), and NNH of 17 (vs 15 and 10) (Table 2).

**Table 2.** Yield of Combining PI-RADS Categories and PSAD Using Different Cutoffs

PSAD measure for avoiding biopsy, ng/mL^2^	No. of studies (patients)	Sensitivity (95% CI), %	NPV (95% CI), %	NNH (95% CI)	Unnecessary biopsy avoided (95% CI), %
**In patients with PI-RADS ≤2 index lesion (including no focal lesion)**					
<0.10	8 (1724)^17,21,27,35,41,55,59,67^	83 (69-91)	95 (91-97)	19 (11-33)	39 (28-51)
<0.15	11 (2970)^21,22,23,27,35,41,43,45,55,59,67^	66 (45-83)	94 (88-97)	16 (9-30)	67 (54-78)
<0.20	7 (1499)^21,27,35,41,55,59,67^	36 (28-46)	92 (88-95)	13 (8-21)	89 (84-93)
**In patients with PI-RADS 3 index lesion**					
<0.10	8 (1386)^21,27,35,41,51,54,55,56^	85 (79-90)	93 (86-97)	15 (7-34)	43 (34-53)
<0.15	10 (1454)^21,22,27,35,41,45,50,51,54,67^	70 (49-84)	90 (84-94)	10 (6-16)	66 (52-78)
<0.20	8 (1644)^21,25,27,35,41,50,55,67^	44 (26-65)	87 (82-92)	8 (5-12)	84 (75-90)
**In patients with PI-RADS ≥4 index lesion**					
<0.10	6 (2733)^21,27,35,41,55,67^	86 (81-90)	64 (59-69)	3 (2-3)	38 (29-48)
<0.15	7 (2716)^21,22,27,35,41,45,67^	75 (52-89)	55 (49-60)	2 (2-2)	64 (54-73)
<0.20	7 (3114)^21,25,27,35,41,55,67^	49 (38-60)	53 (43-63)	2 (2-3)	79 (72-84)
**In patients with PI-RADS ≤3 index lesion (including no focal lesion)**					
<0.10	6 (5288)^21,27,35,41,55,67^	97 (95-98)	94 (89-96)	17 (9-27)	30 (25-34)
<0.15	7 (5225)^21,22,27,35,42,45,67^	95 (90-98)	93 (87-97)	15 (8-29)	48 (40-56)
<0.20	6 (5288)^21,27,35,41,55,67^	87 (85-92)	90 (84-94)	10 (6-16)	59 (53-64)

Abbreviations: NNH, number needed to harm; NPV, negative predictive value; PI-RADS, Prostate Imaging Reporting & Data System; PSAD, prostate-specific antigen density.

## Discussion

The aim of our meta-analysis exploring the independent risk factors of csPCa and assessing the added value of combining PI-RADS and clinical parameters was to improve biopsy decision-making for men with suspected csPCa. The findings of this analysis suggest that PI-RADS 4 and 5 lesions, but not PI-RADS 3 lesions, are significant imaging risk factors of csPCa. Among clinical parameters, only PSAD-related factors (ie, total PSA and prostate volume) were found to be independent risk factors of csPCa when considered together with PI-RADS category. The strategy to forego biopsy in men with PI-RADS 3 or less and PSAD less than 0.10 ng/mL^2^ or less than 0.15 ng/mL^2^ would avoid 30% or 48% of unnecessary biopsies, respectively, while maintaining sensitivity of 97% or 95%.

The literature search yielded narrative review articles on MRI-based strategies in prostate cancer diagnosis.^95,96^ These articles discussed the variable diagnostic performance of the proposed strategies, with an overall area under the receiver operating characteristic curve of 0.64 to 0.93 for detecting csPCa. Our study was strengthened by implementing robust inclusion and exclusion criteria, minimizing verification bias since all included patients in our meta-analysis underwent prostate biopsy even after a negative prebiopsy MRI, performing a comprehensive meta-analysis and quality assessments, generating a simplified practical conclusion from a large number of studies, and having a lack of significant publication bias.

Many of the included studies in our meta-analysis combined MRI and clinical data by creating either (1) risk calculators using nomogram regression equations and/or machine learning models or (2) biopsy strategies using a stepwise approach. Despite the promising performance, the proposed approaches could not be integrated into clinical practice owing to several limitations. First, output of the risk calculators is on a sliding scale representing the likelihood of csPCa, which should be categorized into low vs high likelihood in order to identify patients requiring biopsy. However, the suggested risk threshold for performing a biopsy varied among studies and needs to be adjusted based on the net benefit trade-off between improving diagnostic accuracy and reducing unnecessary biopsies. Second, these models mostly require further external validation and recalibration to ensure their satisfactory performance prior to implementation in clinical practice.^15^ Deniffel et al^15^ found that the overall net benefit of risk calculators ranged from not useful to harmful if used without recalibration. Third, variable sets of clinical parameters were deployed to develop models. Fourth, given the growing number of risk calculators and biopsy strategies, it is challenging for clinicians to choose 1 over the others. Finally, studies showed a probable superiority of a combined MRI and PSAD strategy by comparing risk calculators in terms of reducing unnecessary biopsies without missing csPCa.^15^ Thereby, pooling results of previously published studies sheds light on the optimal approach of combining MRI and clinical data for prostate biopsy decision-making.

To date, the clinical importance of PI-RADS 3 or less lesions is conflicting, and it is uncertain whether patients with PI-RADS 3 or less or no focal lesions require a biopsy.^7^ At most institutions, men with suspected csPCa still undergo prostate biopsy even after negative MRI results.^2,3,4,9^ The Prospective Assessment of Image Registration in the Diagnosis of Prostate Cancer trial found that 15% of patients with negative MRI results had csPCa.^97^ In other studies, the range of csPCa in men with PI-RADS 3 and PI-RADS 1 or 2 lesions ranged from 3% to 46% and 0% to 17%, respectively.^7,98,99^ The observed variation could be due to several factors, including heterogenous patient populations and suboptimal interobserver agreement of PI-RADS.^100,101,102^ Our results suggest that combining PI-RADS with PSAD would reduce the number of unnecessary biopsies and improve the diagnostic yield. Although the stepwise approach based on PI-RADS and PSAD has been used in some institutions to drive decision-making toward prostate biopsy, the current guidelines do not advise against biopsies in patients with a low PSAD and equivocal MRI findings given a lack of level 1 evidence. This meta-analysis provides evidence that could potentially influence the evolution of these guidelines.

### Limitations

This analysis has some limitations. First, this study-level meta-analysis was based on published data rather than individual patient data; thus, we were unable to adjust our findings for patient-level confounders. Second, some clinical variables were assessed and reported by only a few studies, which limited our ability to investigate the importance of those factors, such as family history of csPCa, race and ethnicity, genomic analysis, PCa antigen 3 test, and other novel serum and urine biomarkers, in estimating csPCa. Third, regarding stepwise biopsy strategies, the published literature has mainly focused on the yield of combining PI-RADS with PSAD and/or total PSA; we did not have sufficient evidence-based literature on all other clinical parameters to perform a further diagnostic meta-analysis. Exploring other PSAD cutoff points and additional variables like age may have some added value in reducing unnecessary biopsies; however, the current lack of studies on these approaches limits our ability to conduct meta-analyses. Furthermore, the results of pooled analysis for the stepwise biopsy strategy combining PI-RADS with PSAD were driven by 6 to 11 studies comprising 1454 to 5288 patients. Fourth, several PSA-related analyses, such as the Four Kallikrein score,^103^ the Prostate Health Index,^104^ ConfirmMDx,^105^ and SelectMDx,^106^ have been proposed to guide prostate decision-making. Since these scores and indices have been developed by combining clinical factors, including total PSA, we did not include them separately in our model. Fifth, we observed high between-study heterogeneity in the rate of csPCa due to different patient populations, which was addressed by using random-effects models and performing meta-regression analyses. Sixth, non–English-language articles were excluded, which may have resulted in some studies being missed.

## Conclusions

The need to identify men requiring a prostate biopsy remains a key issue in the diagnosis of PCa. Results of our systematic review and meta-analysis suggest that prostate biopsy might be avoided in men with negative or equivocal MRI results and low PSAD. Despite the high sensitivity, 3% to 5% of csPCa cases may still be missed with this approach. This concern can be addressed by future prospective studies using a lower threshold for PSAD and incorporating additional variables for further risk stratification. In addition, we can assess effective follow-up approaches after a decision not to perform a biopsy is made, especially since this decision-making process would need to occur over a person’s lifetime.

## Notes

Supplement 1. **eMethods 1.** Search Strategies **eMethods 2.** Title and Abstract Screening Inclusion and Exclusion Criteria **eMethods 3.** Full-Text Review Stepwise Approach **eMethods 4.** Details of the Quality Assessment With the Newcastle-Ottawa Scale **eTable 1.** Results of the Quality Assessment With the Newcastle-Ottawa Scale **eMethods 5.** Details of the Quality Assessment With the QUADAS-2 Tool **eTable 2.** Results of the Quality Assessment With the QUADAS-2 Tool **eFigure 1.** Pooled Results of the Quality Assessment With the QUADAS-2 Tool **eMethods 6.** Methodology and Analytic Approach: Multimodel Inference Method **eTable 3.** Study Design of the Included Studies **eTable 4.** Baseline Characteristics of Included Studies **eTable 5.** Baseline Characteristics of the Included Studies: csPCa vs Non-cs PCa (ie, Nonsignificant PCa and Benign) **eFigure 2.** Funnel Plot **eFigure 3.** Univariable Meta-Regression for the Association Between the Rate of csPCa and Clinical Parameters **eFigure 4.** Univariable Meta-Regression for the Association Between the Rate of csPCa and Prostate Volume/Clinical Parameters **eFigure 5.** Univariable Meta-Regression for the Association Between the Rate of csPCa and Clinical/Imaging Parameters **eFigure 6**. Intercorrelation Matrix Between Risk Factors of csPCa **eTable 6.** Multiple Variable Meta-Regression on a Subset of Studies: Subgroup Analysis
eReferences

Supplement 2.
Data Sharing Statement

## References

[1] Worldwide cancer data. World Cancer Research Fund International. Accessed January 6, 2023. https://www.wcrf.org/cancer-trends/worldwide-cancer-data/

[2] NCCN guidelines: treatment by cancer type. National Comprehensive Cancer Network. Accessed January 12, 2023. https://www.nccn.org/guidelines/category_1

[3] Mottet N, van den Bergh RCN, Briers E, . EAU-EANM-ESTRO-ESUR-SIOG guidelines on prostate cancer-2020 update: part 1: screening, diagnosis, and local treatment with curative intent. Eur Urol. 2021;79(2):243-262. doi:10.1016/j.eururo.2020.09.042 33172724

[4] *Prostate Cancer: Diagnosis and Management*. National Institute for Health and Care Excellence; 2014. Accessed January 6, 2023. https://www.nice.org.uk/guidance/cg175

[5] Barentsz JO, Weinreb JC, Verma S, . Synopsis of the PI-RADS v2 guidelines for multiparametric prostate magnetic resonance imaging and recommendations for use. Eur Urol. 2016;69(1):41-49. doi:10.1016/j.eururo.2015.08.038 26361169 PMC6364687

[6] Woo S, Suh CH, Kim SY, Cho JY, Kim SH. Diagnostic performance of prostate imaging reporting and data system version 2 for detection of prostate cancer: a systematic review and diagnostic meta-analysis. Eur Urol. 2017;72(2):177-188. doi:10.1016/j.eururo.2017.01.042 28196723

[7] Maggi M, Panebianco V, Mosca A, . Prostate imaging reporting and data system 3 category cases at multiparametric magnetic resonance for prostate cancer: a systematic review and meta-analysis. Eur Urol Focus. 2020;6(3):463-478. doi:10.1016/j.euf.2019.06.014 31279677

[8] Ahmed HU, El-Shater Bosaily A, Brown LC, ; PROMIS Study Group. Diagnostic accuracy of multi-parametric MRI and TRUS biopsy in prostate cancer (PROMIS): a paired validating confirmatory study. Lancet. 2017;389(10071):815-822. doi:10.1016/S0140-6736(16)32401-1 28110982

[9] Early detection of prostate cancer (2018). American Urological Association. Accessed January 12, 2023. https://www.auanet.org/guidelines-and-quality/guidelines/prostate-cancer-early-detection-guideline

[10] Radtke JP, Giganti F, Wiesenfarth M, . Prediction of significant prostate cancer in biopsy-naïve men: validation of a novel risk model combining MRI and clinical parameters and comparison to an ERSPC risk calculator and PI-RADS. PLoS One. 2019;14(8):e0221350. doi:10.1371/journal.pone.0221350 31450235 PMC6710031

[11] Bittencourt LK, Guricova K, Zucker I, Durieux JC, Schoots IG. Risk-based MRI-directed diagnostic pathway outperforms non-risk-based pathways in suspected prostate cancer biopsy-naïve men: a large cohort validation study. Eur Radiol. 2022;32(4):2330-2339. doi:10.1007/s00330-021-08407-6 35028750

[12] Lendínez-Cano G, Ojeda-Claro AV, Gómez-Gómez E, ; AEU-PIEM/2018/000 Investigators. Prospective study of diagnostic accuracy in the detection of high-grade prostate cancer in biopsy-naïve patients with clinical suspicion of prostate cancer who underwent the Select MDx test. Prostate. 2021;81(12):857-865. doi:10.1002/pros.24182 34184761

[13] Sonmez G, Demirtas T, Tombul ST, Akgun H, Demirtas A. Diagnostic efficiency of systemic immune-inflammation index in fusion prostate biopsy. Actas Urol Esp (Engl Ed). 2021;45(5):359-365. doi:10.1016/j.acuro.2020.08.015 34088435

[14] Keck B, Borkowetz A, Poellmann J, . Serum miRNAs support the indication for MRI-ultrasound fusion-guided biopsy of the prostate in patients with low-PI-RADS lesions. Cells. 2021;10(6):1315. doi:10.3390/cells10061315 34070529 PMC8226644

[15] Deniffel D, Healy GM, Dong X, . Avoiding unnecessary biopsy: MRI-based risk models versus a PI-RADS and PSA density strategy for clinically significant prostate cancer. Radiology. 2021;300(2):369-379. doi:10.1148/radiol.2021204112 34032510

[16] Tosun M, Uslu H. Prebiopsy multiparametric MRI and PI-RADS version 2.0 for differentiating histologically benign prostate disease from prostate cancer in biopsies: a retrospective single-center comparison. Clin Imaging. 2021;78:98-103. doi:10.1016/j.clinimag.2021.03.011 33773450

[17] Liang L, Qi F, Cheng Y, . Analysis of risk factors for determining the need for prostate biopsy in patients with negative MRI. Sci Rep. 2021;11(1):6048. doi:10.1038/s41598-021-83802-z 33723287 PMC7960992

[18] Fan YH, Pan PH, Cheng WM, . The Prostate Health Index aids multi-parametric MRI in diagnosing significant prostate cancer. Sci Rep. 2021;11(1):1286. doi:10.1038/s41598-020-78428-6 33674631 PMC7935887

[19] Noh TI, Hyun CW, Kang HE, . A predictive model based on bi-parametric magnetic resonance imaging and clinical parameters for clinically significant prostate cancer in the Korean population. Cancer Res Treat. 2021;53(4):1148-1155. doi:10.4143/crt.2020.1068 33421975 PMC8524004

[20] Apfelbeck M, Pfitzinger P, Bischoff R, . Predictive clinical features for negative histopathology of MRI/ultrasound-fusion-guided prostate biopsy in patients with high likelihood of cancer at prostate MRI: analysis from a urologic outpatient clinic. Clin Hemorheol Microcirc. 2020;76(4):503-511. doi:10.3233/CH-209225 33337358

[21] Falagario UG, Jambor I, Lantz A, . Combined use of prostate-specific antigen density and magnetic resonance imaging for prostate biopsy decision planning: a retrospective multi-institutional study using the Prostate Magnetic Resonance Imaging Outcome Database (PROMOD). Eur Urol Oncol. 2021;4(6):971-979. doi:10.1016/j.euo.2020.08.014 32972896

[22] Sokhi HK, Padhani AR, Patel S, Pope A. Diagnostic yields in patients with suspected prostate cancer undergoing MRI as the first-line investigation in routine practice. Clin Radiol. 2020;75(12):950-956. doi:10.1016/j.crad.2020.08.011 32919755

[23] Anastay V, Gondran-Tellier B, McManus R, . Nonsuspicious prebiopsy multiparametric MRI: is prostate biopsy still necessary? Abdom Radiol (NY). 2020;45(12):4160-4165. doi:10.1007/s00261-020-02728-8 32902661

[24] Sonmez G, Tombul ST, Demirtas T, Demirtas A. Clinical factors for predicting malignancy in patients with PSA < 10 ng/mL and PI-RADS 3 lesions. Asia Pac J Clin Oncol. 2021;17(2):e94-e99. doi:10.1111/ajco.13347 32779392

[25] Kim M, Ryu H, Lee HJ, Hwang SI, Choe G, Hong SK. Who can safely evade a magnetic resonance imaging fusion-targeted biopsy (MRIFTB) for prostate imaging reporting and data system (PI-RADS) 3 lesion? World J Urol. 2021;39(5):1463-1471. doi:10.1007/s00345-020-03352-3 32696126

[26] Busetto GM, Del Giudice F, Maggi M, . Prospective assessment of two-gene urinary test with multiparametric magnetic resonance imaging of the prostate for men undergoing primary prostate biopsy. World J Urol. 2021;39(6):1869-1877. doi:10.1007/s00345-020-03359-w 32681273 PMC8217060

[27] Stevens E, Truong M, Bullen JA, Ward RD, Purysko AS, Klein EA. Clinical utility of PSAD combined with PI-RADS category for the detection of clinically significant prostate cancer. Urol Oncol. 2020;38(11):846.e9-846.e16. doi:10.1016/j.urolonc.2020.05.024 32576527

[28] Wei CG, Chen T, Zhang YY, . Biparametric prostate MRI and clinical indicators predict clinically significant prostate cancer in men with “gray zone” PSA levels. Eur J Radiol. 2020;127:108977. doi:10.1016/j.ejrad.2020.108977 32330776

[29] Al Hussein Al Awamlh B, Marks LS, Sonn GA, . Multicenter analysis of clinical and MRI characteristics associated with detecting clinically significant prostate cancer in PI-RADS (v2.0) category 3 lesions. Urol Oncol. 2020;38(7):637.e9-637.e15. doi:10.1016/j.urolonc.2020.03.019 32307327 PMC7328785

[30] Han C, Liu S, Qin XB, Ma S, Zhu LN, Wang XY. MRI combined with PSA density in detecting clinically significant prostate cancer in patients with PSA serum levels of 4∼10ng/mL: biparametric versus multiparametric MRI. Diagn Interv Imaging. 2020;101(4):235-244. doi:10.1016/j.diii.2020.01.014 32063483

[31] He BM, Shi ZK, Li HS, . A novel prediction tool based on multiparametric magnetic resonance imaging to determine the biopsy strategy for clinically significant prostate cancer in patients with PSA levels less than 50 ng/mL. Ann Surg Oncol. 2020;27(4):1284-1295. doi:10.1245/s10434-019-08111-2 31848822

[32] Borque-Fernando Á, Esteban LM, Celma A, . How to implement magnetic resonance imaging before prostate biopsy in clinical practice: nomograms for saving biopsies. World J Urol. 2020;38(6):1481-1491. doi:10.1007/s00345-019-02946-w 31506748

[33] Hsieh PF, Li WJ, Lin WC, . Combining prostate health index and multiparametric magnetic resonance imaging in the diagnosis of clinically significant prostate cancer in an Asian population. World J Urol. 2020;38(5):1207-1214. doi:10.1007/s00345-019-02889-2 31440806 PMC7190581

[34] Lu YF, Zhang Q, Chen HY, . Improving the detection rate of prostate cancer in the gray zone of PI-RADS v2 and serum tPSA by using prostate-specific antigen-age volume. Medicine (Baltimore). 2019;98(26):e16289. doi:10.1097/MD.0000000000016289 31261602 PMC6616591

[35] Boesen L, Nørgaard N, Løgager V, . Prebiopsy biparametric magnetic resonance imaging combined with prostate-specific antigen density in detecting and ruling out Gleason 7-10 prostate cancer in biopsy-naïve men. Eur Urol Oncol. 2019;2(3):311-319. doi:10.1016/j.euo.2018.09.001 31200846

[36] Lu YF, Zhang Q, Yao WG, . Optimizing prostate cancer accumulating model: combined PI-RADS v2 with prostate specific antigen and its derivative data. Cancer Imaging. 2019;19(1):26. doi:10.1186/s40644-019-0208-6 31122297 PMC6533650

[37] Boesen L, Thomsen FB, Nørgaard N, . A predictive model based on biparametric magnetic resonance imaging and clinical parameters for improved risk assessment and selection of biopsy-naïve men for prostate biopsies. Prostate Cancer Prostatic Dis. 2019;22(4):609-616. doi:10.1038/s41391-019-0149-y 30988407

[38] Bhat NR, Vetter JM, Andriole GL, Shetty AS, Ippolito JE, Kim EH. Magnetic resonance imaging-defined prostate-specific antigen density significantly improves the risk prediction for clinically significant prostate cancer on biopsy. Urology. 2019;126:152-157. doi:10.1016/j.urology.2018.12.010 30580005

[39] Kim TJ, Lee MS, Hwang SI, Lee HJ, Hong SK. Outcomes of magnetic resonance imaging fusion-targeted biopsy of prostate imaging reporting and data system 3 lesions. World J Urol. 2019;37(8):1581-1586. doi:10.1007/s00345-018-2565-3 30460594

[40] Cuocolo R, Stanzione A, Rusconi G, . PSA-density does not improve bi-parametric prostate MR detection of prostate cancer in a biopsy naïve patient population. Eur J Radiol. 2018;104:64-70. doi:10.1016/j.ejrad.2018.05.004 29857868

[41] Hansen NL, Barrett T, Kesch C, . Multicentre evaluation of magnetic resonance imaging supported transperineal prostate biopsy in biopsy-naïve men with suspicion of prostate cancer. BJU Int. 2018;122(1):40-49. doi:10.1111/bju.14049 29024425

[42] Radtke JP, Wiesenfarth M, Kesch C, . Combined clinical parameters and multiparametric magnetic resonance imaging for advanced risk modeling of prostate cancer-patient-tailored risk stratification can reduce unnecessary biopsies. Eur Urol. 2017;72(6):888-896. doi:10.1016/j.eururo.2017.03.039 28400169

[43] Distler FA, Radtke JP, Bonekamp D, . The value of PSA density in combination with PI-RADS for the accuracy of prostate cancer prediction. J Urol. 2017;198(3):575-582. doi:10.1016/j.juro.2017.03.130 28373135

[44] van Leeuwen PJ, Hayen A, Thompson JE, . A multiparametric magnetic resonance imaging-based risk model to determine the risk of significant prostate cancer prior to biopsy. BJU Int. 2017;120(6):774-781. doi:10.1111/bju.13814 28207981

[45] Washino S, Okochi T, Saito K, . Combination of prostate imaging reporting and data system (PI-RADS) score and prostate-specific antigen (PSA) density predicts biopsy outcome in prostate biopsy naïve patients. BJU Int. 2017;119(2):225-233. doi:10.1111/bju.13465 26935594

[46] Mehralivand S, Shih JH, Rais-Bahrami S, . A magnetic resonance imaging-based prediction model for prostate biopsy risk stratification. JAMA Oncol. 2018;4(5):678-685. doi:10.1001/jamaoncol.2017.5667 29470570 PMC5885194

[47] Alberts AR, Roobol MJ, Verbeek JFM, . Prediction of high-grade prostate cancer following multiparametric magnetic resonance imaging: improving the Rotterdam European Randomized Study of Screening for Prostate Cancer risk calculators. Eur Urol. 2019;75(2):310-318. doi:10.1016/j.eururo.2018.07.031 30082150

[48] Sakaguchi K, Hayashida M, Tanaka N, Oka S, Urakami S. A risk model for detecting clinically significant prostate cancer based on bi-parametric magnetic resonance imaging in a Japanese cohort. Sci Rep. 2021;11(1):18829. doi:10.1038/s41598-021-98195-2 34552143 PMC8458280

[49] Liu G, Zhu Y, Yao Z, Jiang Y, Wu B, Bai S. Development and validation of a predictive model for determining clinically significant prostate cancer in men with negative magnetic resonance imaging after transrectal ultrasound-guided prostate biopsy. Prostate. 2021;81(13):983-991. doi:10.1002/pros.24193 34254330

[50] Zhang Y, Zeng N, Zhang FB, Rui Huang YX, Tian Y. Performing precise biopsy in naive patients with equivocal PI-RADS, version 2, score 3, lesions: an MRI-based nomogram to avoid unnecessary surgical intervention. Clin Genitourin Cancer. 2020;18(5):367-377. doi:10.1016/j.clgc.2019.11.011 32771334

[51] Görtz M, Radtke JP, Hatiboglu G, . The value of prostate-specific antigen density for Prostate Imaging-Reporting and Data System 3 lesions on multiparametric magnetic resonance imaging: a strategy to avoid unnecessary prostate biopsies. Eur Urol Focus. 2021;7(2):325-331. doi:10.1016/j.euf.2019.11.012 31839564

[52] Saba K, Wettstein MS, Lieger L, . External validation and comparison of prostate cancer risk calculators incorporating multiparametric magnetic resonance imaging for prediction of clinically significant prostate cancer. J Urol. 2020;203(4):719-726. doi:10.1097/JU.0000000000000622 31651228

[53] Petersmann AL, Remmers S, Klein T, . External validation of two MRI-based risk calculators in prostate cancer diagnosis. World J Urol. 2021;39(11):4109-4116. doi:10.1007/s00345-021-03770-x 34169337

[54] Tan TW, Png KS, Lee CH, . MRI fusion-targeted transrectal prostate biopsy and the role of prostate-specific antigen density and prostate health index for the detection of clinically significant prostate cancer in Southeast Asian Men. J Endourol. 2017;31(11):1111-1116. doi:10.1089/end.2017.0485 28797178

[55] Hansen NL, Barrett T, Koo B, . The influence of prostate-specific antigen density on positive and negative predictive values of multiparametric magnetic resonance imaging to detect Gleason score 7-10 prostate cancer in a repeat biopsy setting. BJU Int. 2017;119(5):724-730. doi:10.1111/bju.13619 27488931

[56] Thompson JE, van Leeuwen PJ, Moses D, . The diagnostic performance of multiparametric magnetic resonance imaging to detect significant prostate cancer. J Urol. 2016;195(5):1428-1435. doi:10.1016/j.juro.2015.10.140 26529298

[57] Zhu H, Ding XF, Lu SM, . The application of biopsy density in transperineal templated-guided biopsy patients with PI-RADS<3. Front Oncol. 2022;12:918300 doi:10.3389/fonc.2022.918300 35756615 PMC9214307

[58] Morote J, Borque-Fernando A, Triquell M, . Comparative analysis of PSA density and an MRI-based predictive model to improve the selection of candidates for prostate biopsy. Cancers (Basel). 2022;14(10):2374. doi:10.3390/cancers14102374 35625978 PMC9139805

[59] Gan JM, Kikano EG, Smith DA, . Clinically significant prostate cancer detection after a negative prebiopsy MRI examination: comparison of biparametric versus multiparametric MRI. AJR Am J Roentgenol. 2022;218(5):859-866. doi:10.2214/AJR.21.26569 34817189

[60] Zhang CC, Tu X, Lin TH, . The role of prostate-specific antigen density and negative multiparametric magnetic resonance imaging in excluding prostate cancer for biopsy-naïve men: clinical outcomes from a high-volume center in China. Asian J Androl. 2022;24(6):615-619. doi:10.4103/aja202220 35532555 PMC9809478

[61] Zhou Z, Liang Z, Zuo Y, . Development of a nomogram combining multiparametric magnetic resonance imaging and PSA-related parameters to enhance the detection of clinically significant cancer across different region. Prostate. 2022;82(5):556-565. doi:10.1002/pros.24302 35098557

[62] van Riel LAMJG, Jager A, Meijer D, . Predictors of clinically significant prostate cancer in biopsy-naïve and prior negative biopsy men with a negative prostate MRI: improving MRI-based screening with a novel risk calculator. Ther Adv Urol. 2022;14:17562872221088536. doi:10.1177/17562872221088536 35356754 PMC8958520

[63] Morote J, Campistol M, Triquell M, . Improving the early detection of clinically significant prostate cancer in men in the challenging prostate imaging-reporting and data system 3 category. Eur Urol Open Sci. 2022;37:38-44. doi:10.1016/j.euros.2021.12.009 35243388 PMC8883194

[64] Wagaskar VG, Lantz A, Sobotka S, . Development and external validation of a prediction model to identify candidates for prostate biopsy. Urol J. 2022;19(5):379-385. doi:10.22037/uj.v18i.6852 34978065

[65] Chau EM, Russell B, Santaolalla A, . MRI-based nomogram for the prediction of prostate cancer diagnosis: a multi-centre validated patient–physician decision tool. J Clin Urol. 2023;16(6):588-595. doi:10.1177/20514158211065949

[66] Frisbie JW, Van Besien AJ, Lee A, . PSA density is complementary to prostate MP-MRI PI-RADS scoring system for risk stratification of clinically significant prostate cancer. Prostate Cancer Prostatic Dis. Published online May 6, 2022. doi:10.1038/s41391-022-00549-y 35523940

[67] Girometti R, Giannarini G, Panebianco V, . Comparison of different thresholds of PSA density for risk stratification of PI-RADSv2.1 categories on prostate MRI. Br J Radiol. 2022;95(1131):20210886. doi:10.1259/bjr.20210886 34762506 PMC8978227

[68] Wei X, Xu J, Zhong S, . Diagnostic value of combining PI-RADS v2.1 with PSAD in clinically significant prostate cancer. Abdom Radiol (NY). 2022;47(10):3574-3582. doi:10.1007/s00261-022-03592-4 35788882

[69] Pan JF, Su R, Cao JZ, . Modified predictive model and nomogram by incorporating prebiopsy biparametric magnetic resonance imaging with clinical indicators for prostate biopsy decision making. Front Oncol. 2021;11:740868. doi:10.3389/fonc.2021.740868 34589437 PMC8473816

[70] Ryoo H, Kang MY, Sung HH, . Detection of prostate cancer using prostate imaging reporting and data system score and prostate-specific antigen density in biopsy-naive and prior biopsy-negative patients. Prostate Int. 2020;8(3):125-129. doi:10.1016/j.prnil.2020.03.003 33102394 PMC7557180

[71] Deniffel D, Zhang Y, Salinas E, Satkunasivam R, Khalvati F, Haider MA. Reducing unnecessary prostate multiparametric magnetic resonance imaging by using clinical parameters to predict negative and indeterminate findings. J Urol. 2020;203(2):292-298. doi:10.1097/JU.0000000000000518 31479397

[72] Campistol M, Morote J, Triquell M, . Comparison of Proclarix, PSA density and MRI-ERSPC risk calculator to select patients for prostate biopsy after mpMRI. Cancers (Basel). 2022;14(11):2702. doi:10.3390/cancers14112702 35681685 PMC9179369

[73] Hogan D, Yao H, Kanagarajah A, . Can multi-parametric magnetic resonance imaging and prostate-specific antigen density accurately stratify patients prior to prostate biopsy? J Clin Urol. Published online April 12, 2022. doi:10.1177/20514158221084820

[74] Maggi M, Del Giudice F, Falagario UG, . SelectMDx and multiparametric magnetic resonance imaging of the prostate for men undergoing primary prostate biopsy: a prospective assessment in a multi-institutional study. Cancers (Basel). 2021;13(9):2047. doi:10.3390/cancers13092047 33922626 PMC8122883

[75] Wei C, Pan P, Chen T, . A nomogram based on PI-RADS v2.1 and clinical indicators for predicting clinically significant prostate cancer in the transition zone. Transl Androl Urol. 2021;10(6):2435-2446. doi:10.21037/tau-21-49 34295730 PMC8261422

[76] Wang ZB, Wei CG, Zhang YY, . The role of PSA density among PI-RADS v2.1 categories to avoid an unnecessary transition zone biopsy in patients with PSA 4-20 ng/mL. Biomed Res Int. 2021;2021:3995789. doi:10.1155/2021/3995789 34671673 PMC8523253

[77] Morote J, Schwartzman I, Borque A, . Prediction of clinically significant prostate cancer after negative prostate biopsy: the current value of microscopic findings. Urol Oncol. 2021;39(7):432.e11-432.e19. doi:10.1016/j.urolonc.2020.10.016 33160846

[78] Püllen L, Radtke JP, Wiesenfarth M, . External validation of novel magnetic resonance imaging-based models for prostate cancer prediction. BJU Int. 2020;125(3):407-416. doi:10.1111/bju.14958 31758738

[79] Truong M, Wang B, Gordetsky JB, . Multi-institutional nomogram predicting benign prostate pathology on magnetic resonance/ultrasound fusion biopsy in men with a prior negative 12-core systematic biopsy. Cancer. 2018;124(2):278-285. doi:10.1002/cncr.31051 28976544

[80] Záleský M, Stejskal J, Adamcova V, . Use of prostate specific antigen density combined with multiparametric magnetic resonance imaging improves triage for prostate biopsy. Urol Int. 2019;103(1):33-40. doi:10.1159/000500350 31067560

[81] Punnen S, Nahar B, Soodana-Prakash N, . Optimizing patient’s selection for prostate biopsy: a single institution experience with multi-parametric MRI and the 4Kscore test for the detection of aggressive prostate cancer. PLoS One. 2018;13(8):e0201384. doi:10.1371/journal.pone.0201384 30092002 PMC6084850

[82] Cumpston M, Li T, Page MJ, . Updated guidance for trusted systematic reviews: a new edition of the Cochrane Handbook for Systematic Reviews of Interventions. Cochrane Database Syst Rev. 2019;10(10):ED000142. doi:10.1002/14651858.ED000142 31643080 PMC10284251

[83] Page MJ, McKenzie JE, Bossuyt PM, . The PRISMA 2020 statement: an updated guideline for reporting systematic reviews. BMJ. 2021;372(71):n71. doi:10.1136/bmj.n71 33782057 PMC8005924

[84] Kasivisvanathan V, Rannikko AS, Borghi M, ; PRECISION Study Group Collaborators. MRI-targeted or standard biopsy for prostate-cancer diagnosis. N Engl J Med. 2018;378(19):1767-1777. doi:10.1056/NEJMoa1801993 29552975 PMC9084630

[85] Egevad L, Delahunt B, Srigley JR, Samaratunga H. International Society of Urological Pathology (ISUP) grading of prostate cancer—an ISUP consensus on contemporary grading. APMIS. 2016;124(6):433-435. doi:10.1111/apm.12533 27150257

[86] Covidence. Home page. Accessed January 10, 2024. http://www.covidence.org

[87] Wells GA, Shea B, O’Connell D, . The Newcastle-Ottawa Scale (NOS) for Assessing the Quality of Nonrandomised Studies in Meta-Analyses. The Ottawa Hospital; 2000.

[88] Whiting PF, Rutjes AWS, Westwood ME, ; QUADAS-2 Group. QUADAS-2: a revised tool for the quality assessment of diagnostic accuracy studies. Ann Intern Med. 2011;155(8):529-536. doi:10.7326/0003-4819-155-8-201110180-00009 22007046

[89] Stijnen T, Hamza TH, Ozdemir P. Random effects meta-analysis of event outcome in the framework of the generalized linear mixed model with applications in sparse data. Stat Med. 2010;29(29):3046-3067. doi:10.1002/sim.4040 20827667

[90] Harrer M, Cuijpers P, Furukawa TA, Ebert DD. Doing Meta-Analysis With R: A Hands-On Guide. Chapman and Hall/CRC; 2021. doi:10.1201/9781003107347

[91] Higgins JPT, Thompson SG, Deeks JJ, Altman DG. Measuring inconsistency in meta-analyses. BMJ. 2003;327(7414):557-560. doi:10.1136/bmj.327.7414.557 12958120 PMC192859

[92] Egger M, Davey Smith G, Schneider M, Minder C. Bias in meta-analysis detected by a simple, graphical test. BMJ. 1997;315(7109):629-634. doi:10.1136/bmj.315.7109.629 9310563 PMC2127453

[93] Mansfield ER, Helms BP. Detecting multicollinearity. Am Stat. 1982;36(3a):158-160. doi:10.1080/00031305.1982.10482818

[94] Model selection using the glmulti and MuMIn packages. the metafor package: a meta-analysis package for R. Accessed January 12, 2023. https://www.metafor-project.org/doku.php/tips:model_selection_with_glmulti_and_mumin

[95] Schoots IG, Roobol MJ. Multivariate risk prediction tools including MRI for individualized biopsy decision in prostate cancer diagnosis: current status and future directions. World J Urol. 2020;38(3):517-529. doi:10.1007/s00345-019-02707-9 30868240 PMC7064454

[96] Schoots IG, Padhani AR. Personalizing prostate cancer diagnosis with multivariate risk prediction tools: how should prostate MRI be incorporated? World J Urol. 2020;38(3):531-545. doi:10.1007/s00345-019-02899-0 31399825 PMC7064475

[97] Elkhoury FF, Felker ER, Kwan L, . Comparison of targeted vs systematic prostate biopsy in men who are biopsy naive: the Prospective Assessment of Image Registration in the Diagnosis of Prostate Cancer (PAIREDCAP) study. JAMA Surg. 2019;154(9):811-818. doi:10.1001/jamasurg.2019.1734 31188412 PMC6563598

[98] Park KJ, Choi SH, Lee JS, Kim JK, Kim MH, Jeong IG. Risk stratification of prostate cancer according to PI-RADS version 2 categories: meta-analysis for prospective studies. J Urol. 2020;204(6):1141-1149. doi:10.1097/JU.0000000000001306 32716687

[99] Oerther B, Engel H, Bamberg F, Sigle A, Gratzke C, Benndorf M. Cancer detection rates of the PI-RADSv2.1 assessment categories: systematic review and meta-analysis on lesion level and patient level. Prostate Cancer Prostatic Dis. 2022;25(2):256-263. doi:10.1038/s41391-021-00417-1 34230616 PMC9184264

[100] Annamalai A, Fustok JN, Beltran-Perez J, Rashad AT, Krane LS, Triche BL. Interobserver agreement and accuracy in interpreting mpMRI of the prostate: a systematic review. Curr Urol Rep. 2022;23(1):1-10. doi:10.1007/s11934-022-01084-y 35226257

[101] Glazer DI, Mayo-Smith WW, Sainani NI, . Interreader agreement of Prostate Imaging Reporting and Data System version 2 using an in-bore MRI-guided prostate biopsy cohort: a single institution’s initial experience. AJR Am J Roentgenol. 2017;209(3):W145-W151. doi:10.2214/AJR.16.17551 28657843 PMC5613666

[102] Purysko AS, Bittencourt LK, Bullen JA, Mostardeiro TR, Herts BR, Klein EA. Accuracy and interobserver agreement for prostate imaging reporting and data system, version 2, for the characterization of lesions identified on multiparametric MRI of the prostate. AJR Am J Roentgenol. 2017;209(2):339-349. doi:10.2214/AJR.16.17289 28570099

[103] Darst BF, Chou A, Wan P, . The Four-Kallikrein panel is effective in identifying aggressive prostate cancer in a multiethnic population. Cancer Epidemiol Biomarkers Prev. 2020;29(7):1381-1388. doi:10.1158/1055-9965.EPI-19-1560 32385116 PMC7334056

[104] Perdonà S, Bruzzese D, Ferro M, . Prostate Health Index (PHI) and prostate cancer antigen 3 (PCA3) significantly improve diagnostic accuracy in patients undergoing prostate biopsy. Prostate. 2013;73(3):227-235. doi:10.1002/pros.22561 22821756

[105] Wojno KJ, Costa FJ, Cornell RJ, . Reduced rate of repeated prostate biopsies observed in ConfirmMDx Clinical Utility Field Study. Am Health Drug Benefits. 2014;7(3):129-134. 24991397 PMC4070628

[106] Govers TM, Hessels D, Vlaeminck-Guillem V, . Cost-effectiveness of SelectMDx for prostate cancer in four European countries: a comparative modeling study. Prostate Cancer Prostatic Dis. 2019;22(1):101-109. doi:10.1038/s41391-018-0076-3 30127462

