# Supplementary material for: Magnetic Resonance Imaging, Clinical, and Biopsy Findings in Suspected Prostate Cancer: A Systematic Review and Meta-Analysis
Source: JAMA Netw Open. 2024 Mar 29;7(3):e244258. doi: 10.1001/jamanetworkopen.2024.4258 (PMC10980971; doi:10.1001/jamanetworkopen.2024.4258)
Supplement: Supplement 1. — eMethods 1. Search Strategies eMethods 2. Title and Abstract Screening Inclusion and Exclusion Criteria eMethods 3. Full-Text Review Stepwise Approach eMethods 4. Details of the Quality Assessment With the Newcastle-Ottawa Scale eTable 1. Results of the Quality Assessment With the Newcastle-Ottawa Scale eMethods 5. Details of the Quality Assessment With the QUADAS-2 Tool eTable 2. Results of the Quality Assessment With the QUADAS-2 Tool eFigure 1. Pooled Results of the Quality Assessment With the QUADAS-2 Tool eMethods 6. Methodology and Analytic Approach: Multimodel Inference Method eTable 3. Study Design of the Included Studies eTable 4. Baseline Characteristics of Included Studies eTable 5. Baseline Characteristics of the Included Studies: csPCa vs Non-cs PCa (ie, Nonsignificant PCa and Benign) eFigure 2. Funnel Plot eFigure 3. Univariable Meta-Regression for the Association Between the Rate of csPCa and Clinical Parameters eFigure 4. Univariable Meta-Regression for the Association Between the Rate of csPCa and Prostate Volume/Clinical Parameters eFigure 5. Univariable Meta-Regression for the Association Between the Rate of csPCa and Clinical/Imaging Parameters eFigure 6. Intercorrelation Matrix Between Risk Factors of csPCa eTable 6. Multiple Variable Meta-Regression on a Subset of Studies: Subgroup Analysis eReferences [file jamanetwopen-e244258-s001.pdf]

Supplemental Online Content

Haj-Mirzaian A, Burk KS, Lacson R, et al. Magnetic resonance imaging, clinical, and biopsy findings in suspected prostate cancer: a systematic review and meta-analysis. *JAMA Netw Open*. 2024;7(3):e244258. doi:10.1001/jamanetworkopen.2024.4258

eMethods 1. Search Strategies

eMethods 2. Title and Abstract Screening Inclusion and Exclusion Criteria

eMethods 3. Full-Text Review Stepwise Approach

eMethods 4. Details of the Quality Assessment With the Newcastle-Ottawa Scale

eTable 1. Results of the Quality Assessment With the Newcastle-Ottawa Scale

eMethods 5. Details of the Quality Assessment With the QUADAS-2 Tool

eTable 2. Results of the Quality Assessment With the QUADAS-2 Tool

eFigure 1. Pooled Results of the Quality Assessment With the QUADAS-2 Tool

eMethods 6. Methodology and Analytic Approach: Multimodel Inference Method

eTable 3. Study Design of the Included Studies

eTable 4. Baseline Characteristics of Included Studies

eTable 5. Baseline Characteristics of the Included Studies: csPCa vs Non-csPCa (ie, Nonsignificant PCa and Benign)

eFigure 2. Funnel Plot

eFigure 3. Univariable Meta-Regression for the Association Between the Rate of csPCa and Clinical Parameters

eFigure 4. Univariable Meta-Regression for the Association Between the Rate of csPCa and Prostate Volume/Clinical Parameters

eFigure 5. Univariable Meta-Regression for the Association Between the Rate of csPCa and Clinical/Imaging Parameters

eFigure 6. Intercorrelation Matrix Between Risk Factors of csPCa

eTable 6. Multiple Variable Meta-Regression on a Subset of Studies: Subgroup Analysis

eReferences

This supplemental material has been provided by the authors to give readers additional information about their work.

© 2024 Haj-Mirzaian A et al. *JAMA Network Open.*

eMethods 1. Search Strategies

Databases:

- PubMed/Medline
- EMBASE
- Web of Science Core Collection
- Cochrane Library

Date Searched: July 01, 2022

PubMed/ Medline

	**Search string**	**Category**	**No.**
#1	"prostatic neoplasms"[MeSH Terms] OR ("prostatic"[All Fields] AND "neoplasms"[All Fields]) OR "prostatic neoplasms"[All Fields] OR ("prostate"[All Fields] AND "cancer"[All Fields]) OR "prostate cancer"[All Fields]	Prostate cancer	198,109
#2	"magnetic resonance imaging"[MeSH Terms] OR ("magnetic"[All Fields] AND "resonance"[All Fields] AND "imaging"[All Fields]) OR "magnetic resonance imaging"[All Fields] OR "mri"[All Fields]	MRI	12,246
#3	"biparametric"[All Fields] OR "bi-parametric"[All Fields] OR "multiparametric"[All Fields] OR "multi-parametric"[All Fields] OR "bpMRI"[All Fields] OR "bp-MRI"[All Fields] OR "mpMRI"[All Fields] OR "mp-MRI"[All Fields] OR "PI-RADS"[All Fields] OR ("pirad"[All Fields] OR "pirads"[All Fields]) OR ("Prostate Imaging Reporting & Data System" [All Fields] OR " Prostate Imaging Reporting and Data System" [All Fields])	PI-RADS	4,092
#4	"PSA"[All Fields] OR "prostate specific antigen"[All Fields] OR "PSAD"[All Fields] OR "PSA density"[All Fields] OR "prostate specific antigen density"[All Fields] OR "age"[All Fields] OR "digital rectal exam"[All Fields] OR "DRE"	Clinical factors	1,787
#5	NOT ("animals"[MeSH Terms])	Human	1,493
#6	NOT (English)	Language	1,429
#7	#1 AND #2 AND #3 AND #4, NOT #5, NOT #6	Overall	1,429

© 2024 Haj-Mirzaian A et al. *JAMA Network Open.*

EMBASE

	Search string	Category	No.
#1	'prostate tumor':ab,ti OR 'prostate cancer':ab,ti OR 'prostate neoplasm':ab,ti OR 'prostatic cancer':ab,ti	Prostate cancer	206,366
#2	'mri':ab,ti OR ' magnetic resonance imaging':ab,ti	MRI	12,790
#3	'pi-rads':ab,ti OR 'pirads':ab,ti OR 'Prostate Imaging Reporting & Data System ':ab,ti OR 'Prostate Imaging Reporting and Data System ':ab,ti	PI-RADS	2,280
#4	'psa' OR 'prostate specific antigen' OR 'psad' OR 'psa density' OR 'prostate specific antigen density' OR 'age' OR 'digital rectal exam' OR 'dre	Clinical factors	1,562
#5	NOT (English)	Language	1,532
#6	#1 AND #2 AND #3 AND #4, NOT #5	Overall	1,532

Web of Science Core Collection

	Search string	Category	No.
#1	"prostate cancer" OR "prostate neoplasm" OR "prostatic neoplasm” OR “prostatic cancer" – Title/Abstract	Prostate cancer	110,123
#2	"MRI" OR "magnetic resonance imaging" – Title/Abstract	MRI	6,876
#3	"pi-rads" OR "pirads" OR "Prostate Imaging Reporting & Data System" OR "Prostate Imaging Reporting and Data System" – Title/Abstract	PI-RADS	1,141
#4	"psa" OR "prostate specific antige" OR "psad" OR "psa density" OR "prostate specific antigen density" OR "age" OR "digital rectal exam" OR "dre" – All fields	Clinical factors	528
#5	#1 AND #2 AND #3 AND #4	Overall	528

© 2024 Haj-Mirzaian A et al. *JAMA Network Open.*

Cochrane Library

	Search string	Category	No.
#1	“prostate cancer":ti,ab,kw OR “prostate neoplasm":ti,ab,kw OR “prostatic cancer ":ti,ab,kw OR “prostatic cancers":ti,ab,kw OR “prostate cancers":ti,ab,kw OR “prostate neoplasms":ti,ab,kw OR “prostatic neoplasm":ti,ab,kw OR “prostatic neoplasms":ti,ab,kw	Prostate cancer	1,523
**#2**	“magnetic resonance imaging":ti,ab,kw OR "mri":ti,ab,kw	MRI	109
**#3**	"biparametric":ti,ab,kw OR "multiparametric":ti,ab,kw OR "bpMRI":ti,ab,kw OR "mpmris":ti,ab,kw OR OR "PI-RADS":ti,ab,kw OR "PIRADS":ti,ab,kw	PI-RADS	1
**#4**	NOT animals	Date	1
**#6**	#1 AND #2 AND #3 AND #4 NOT	Overall	1

© 2024 Haj-Mirzaian A et al. *JAMA Network Open.*

eMethods 2. Title and Abstract Screening Inclusion and Exclusion Criteria

Inclusion Criteria

1. Combining PI-RADS with clinical parameters (including but not limited to total PSA, free PSA, free/total PSA ratio, PSAD, age, race, ethnicity, BMI, family history of prostate cancer, digital rectal exam, physical exam, and history of prior biopsy) to reduce unnecessary prostate biopsies.
2. Patients with suspected but not confirmed clinically significant prostate cancer.
3. PI-RADS with prebiopsy mpMRI or bpMRI.
4. Defining clinically significant prostate cancer using pathology.

Exclusion Criteria

1. Animal studies.
2. Case reports, case reports, review articles, conference abstracts, editorials, commentaries, letters, thesis, brief reports, non-English studies.
3. Lack of MRI or pathology.
4. Focus on other imaging like PET or US.
5. Technical or methodology articles.
6. Not combining PI-RADS with clinical parameters.
7. Active surveillance for prostate cancer.
8. Included patients with history of clinically significant prostate cancer.
9. Predicating other factors like lymph node invasion.

© 2024 Haj-Mirzaian A et al. *JAMA Network Open.*

eMethods 3. Full-Text Review Stepwise Approach

1. Does the article enroll and assess patients with suspected, but not confirmed, clinically significant prostate cancer?
   [No restrict definition for clinical suspicion, i.e., suspicion might be made based on elevated PSA, positive digital rectal exam, etc.]
   [Include all studies regardless of the level of suspicion, i.e., include studies on only patients with borderline PSA level and only patients with high PSA and high PI-RADS.]
   No -> STOP. Excluded because of wrong study population.
   Yes -> Proceed to 2.

2. Is there any irrelevant inclusion criteria or exclusion criteria (e.g., enrolling only patients who are on active survilance or with a confirmed diagnosis of clinically significant prostate cancer)?
   No -> Proceed to 3.
   Yes -> STOP. Excluded because of wrong study population.

3. Is a proportion or all enrolled patients evaluated by prostate bp-/mp-MRI and scored by PI-RADS?
   No -> Proceed to 4.
   Yes -> STOP. Excluded because of wrong study design.

4. Is a proportion or all enrolled patients underwnt prostate biopsy after prostate MRI?
   No -> Proceed to 5.
   Yes -> STOP. Excluded because of wrong study design.

5. Is the histopathological data (Gleason score) of the preformed prostate biopsy reported?
   No -> Proceed to 6.
   Yes -> STOP. Excluded because of wrong study design.

6. Is the article of any following study designs?
   [Case report, case series, review article, opinion/editorial, brief report, in-vitro, and animal study]
   No -> Proceed to 7.
   Yes -> STOP. Excluded because of wrong study design.

© 2024 Haj-Mirzaian A et al. *JAMA Network Open.*

7. Is the article published in English?
   No -> STOP. Excluded because of study language.
   Yes-> Proceed to 8.

8. Does the article present the findings of bp/mpMRI (i.e., PI-RADS) and biopsy (i.e., rate of clinically significant prostate cancer) using patient-based data (i.e., not lesion-based data)?
   No -> STOP. Excluded because of the wrong outcome.
   Yes -> Proceed to 9.

9. Does the article specifically combine clinical factors (including but not limited to total PSA, free PSA, free/total PSA ratio, PSAD, age, race, ethnicity, BMI, family history of prostate cancer, digital rectal exam, physical exam, and history of prior biopsy) with PI-RADS, and report its added value in terms of reducing unnecessary biopsies?
   No -> Proceed to 10.
   Yes -> STOP. Excluded because of wrong outcome.

10. Are the presented data of the article were previously presented by other included studies (e.g., the same data and results)?
   No -> Included.
   Yes -> STOP. Excluded because of the published data.

© 2024 Haj-Mirzaian A et al. *JAMA Network Open.*

eMethods 4. Details of the Quality Assessment With The Newcastle–Ottawa Scale

Selection Domain

Representativeness of the exposed cohort

a) Truly representative of the average patient in the community (one star)

b) Somewhat representative of the average patient in the community (one star)

c) No description of the derivation of the cohort

Selection of the non-exposed cohort

[This part is not applicable for our study because of lacking control cohort]

a) Drawn from the same community as the exposed cohort

b) Drawn from a different source

c) No description of the derivation of the non-exposed cohort

Ascertainment of exposure

a) Secure record (e.g., available images and reports using patients' medical records) (one star)

b) Structured interview or physical exam (one star)

c) Written self-report

d) No description

e) Other

Precision of defining exposure

a) Defined based on the positive medical history (e.g., elevated PSA) (one star)

b) Defined based on the positive physical exam (e.g., abnormal DRE) (one star)

c) No description

d) Other

Ascertainment of exposure done prospectively or retrospectively

a) Prospectively (one star)

b) Retrospectively

Demonstration that outcome of interest was not present at start of study

a) Yes (one star)

b) No

c) No description

© 2024 Haj-Mirzaian A et al. *JAMA Network Open.*

Comparability Domain

Comparability of cohorts on the basis of the design or analysis controlled for confounders

[This part is not applicable for our study because of lacking control cohort]

a) The study controls for age, sex and marital status

b) Study controls for other factors

c) Cohorts are not comparable on the basis of the design or analysis controlled for confounders

Outcome Domain

Assessment of outcome

a) Independent blind assessment (one star)

b) Record linkage (e.g., available MRIs and reports using patients' medical records) (one star)

c) Self-report

d) No description

e) Other

Was follow-up long enough for outcomes to occur

a) Yes (e.g., appropriate interval between MRI and biopsy) (one star)

b) No

c) No description

Adequacy of follow-up of cohorts

a) Complete follow-up- all subject accounted for (one star)

b) Subjects lost to follow up unlikely to introduce bias; small number (less than 20 %) lost, or description was provided of those lost (one star)

c) Follow up rate less than 80% and no description of those lost

d) No statement

Categorizing risk of bias (ROB) based on the Newcastle–Ottawa Scale:

- Low ROB: 4 or 5 stars in the selection domain AND 2 or 3 stars in the outcome domain
- Moderate ROB: 3 stars in the selection domain AND 2 or 3 stars in the outcome domain
- High ROB: <3 stars in the selection domain OR <2 stars in the outcome domain

© 2024 Haj-Mirzaian A et al. *JAMA Network Open.*

eTable 1. Results of the Quality Assessment With the Newcastle–Ottawa Scale

	Selection						Outcome				
Study	Representativeness of exposed cohort	Ascertainment of exposure	Precision of defining exposure	Exposure: prospectively or retrospectively	Outcome not present at beginning of study	Total score	Assessment of outcome	Was follow-up long enough?	Adequacy of follow-up	Total score	Risk of Bias (ROB)
Bittencourt et al., 2022	*	*	*	*	*	5	*	*	*	3	Low
Wang et al., 2021	*	*	*	-	*	4	*	*	*	3	Low
Lendínez-Cano et al., 2021	*	*	*	*	*	5	*	*	*	3	Low
Sonmez et al., 2021	*	*	*	-	*	4	*	*	*	3	Low
Keck et al., 2021	*	*	*	*	*	5	*	*	*	3	Low
Deniffel et al., 2021	*	*	*	*	*	5	*	*	*	3	Low
Tosun and Uslu, 2021	-	*	*	-	*	3	*	*	*	3	Moderate
Liang et al., 2021	*	*	*	-	*	4	*	*	*	3	Low
Fan et al., 2021	*	*	*	*	*	5	*	*	*	3	Low
Noh et al., 2020	-	*	*	-	*	4	*	*	*	3	Moderate
Apfelbeck et al., 2020	-	*	*	-	*	4	*	*	*	3	Moderate
Morote et al., 2020	*	*	*	-	*	4	*	*	*	3	Low
Falagario et al., 2020	*	*	*	*	*	5	*	*	*	3	Low
Sokhi et al., 2020	*	*	*	-	*	4	*	*	*	3	Low
Anastay et al., 2020	*	*	*	-	*	4	*	*	*	3	Low
Sonmez et al., 2020	*	*	*	*	*	5	*	*	*	3	Low
Kim et al., 2020	-	*	*	-	*	3	*	*	*	3	Moderate
Busetto et al., 2020	*	*	*	*	*	5	*	*	*	3	Low
Stevens et al., 2020	*	*	*	-	*	4	*	*	*	3	Low
Wei et al., 2020	*	*	*	-	*	4	*	*	*	3	Low

© 2024 Haj-Mirzaian A et al. *JAMA Network Open.*

Study	Selection						Outcome				
	Representativeness of exposed cohort	Ascertainment of exposure	Precision of defining exposure	Exposure: prospectively or retrospectively	Outcome not present at beginning of study	Total score	Assessment of outcome	Was follow-up long enough?	Adequacy of follow-up	Total score	Risk of Bias (ROB)
Al Hussein Al Awamlh et al., 2020	*	*	*	*	*	5	*	*	*	3	Low
Han et al., 2020	-	*	*	-	*	3	*	*	*	3	Moderate
He et al., 2019	*	*	*	-	*	4	*	*	*	3	Low
Borque-Fernando et al., 2019	-	*	*	*	*	4	*	*	*	3	Low
Hsieh et al., 2019	*	*	*	*	*	5	*	*	*	3	Low
Lu, Zhang, Chen, et al., 2019	*	*	*	*	*	5	*	*	*	3	Low
Boesen, Nørgaard, et al., 2019	*	*	*	*	*	5	*	*	*	3	Low
Lu, Zhang, Yao, et al., 2019	-	*	*	-	*	3	*	*	*	3	Moderate
Boesen, Thomsen, et al., 2019	*	*	*	*	*	5	*	*	*	3	Low
Bhat et al., 2018	*	*	*	*	*	5	*	*	*	3	Low
Kim et al., 2018	*	*	*	*	*	5	*	*	*	3	Low
Cuocolo et al., 2018	*	*	*	-	*	4	*	*	*	3	Low
Hansen et al., 2017	*	*	*	*	*	5	*	*	*	3	Low
Radtke et al., 2017	*	*	*	*	*	5	*	*	*	3	Low
Distler et al., 2017	*	*	*	*	*	5	*	*	*	3	Low
van Leeuwen et al., 2017	-	*	*	*	*	4	*	*	*	3	Low
Washino et al., 2016	*	*	*	*	*	5	*	*	*	3	Low
Mehralivand et al., 2018	*	*	*	-	*	4	*	*	*	3	Low
Alberts et al., 2018	*	*	*	*	*	5	*	*	*	3	Low

© 2024 Haj-Mirzaian A et al. *JAMA Network Open.*

Study	Selection						Outcome				
	Representativeness of exposed cohort	Ascertainment of exposure	Precision of defining exposure	Exposure: prospectively or retrospectively	Outcome not present at beginning of study	Total score	Assessment of outcome	Was follow-up long enough?	Adequacy of follow-up	Total score	Risk of Bias (ROB)
Sakaguchi et al., 2021	*	*	*	-	*	4	*	*	*	3	Low
Liu et al., 2021	*	*	*	-	*	4	*	*	*	3	Low
Zhang et al., 2019	*	*	*	*	*	5	*	*	*	3	Low
Görtz et al., 2019	*	*	*	*	*	5	*	*	*	3	Low
Saba et al., 2019	*	*	*	-	*	4	*	*	*	3	Low
Radtke et al., 2019	*	*	*	*	*	5	*	*	*	3	Low
Záleský et al., 2019	-	*	*	*	*	4	*	*	*	3	Low
Punnen et al., 2018	-	*	*	-	*	3	*	*	*	3	Moderate
Truong et al., 2017	*	*	*	-	*	4	*	*	*	3	Low
Tan et al., 2017	*	*	*	*	*	5	*	*	*	3	Low
Hansen et al., 2016	*	*	*	-	*	4	*	*	*	3	Low
Thompson et al., 2015	-	*	*	*	*	4	*	*	*	3	Low
Zhu et al., 2022	*	*	*	-	*	4	*	*	*	3	Low
Campistol et al., 2022	*	*	*	*	*	5	*	*	*	3	Low
Morote, Borque-Fernando, et al., 2022	*	*	*	-	*	4	*	*	*	3	Low
Gan et al., 2022	*	*	*	-	*	4	*	*	*	3	Low
Zhang et al., 2022	*	*	*	-	*	4	*	*	*	3	Low
Zhou et al., 2022	*	*	*	-	*	4	*	*	*	3	Low
van Riel et al., 2022	*	*	*	*	*	5	*	*	*	3	Low
Morote, Campistol, et al., 2022	*	*	*	*	*	5	*	*	*	3	Low

© 2024 Haj-Mirzaian A et al. *JAMA Network Open.*

Study	Selection						Outcome				Risk of Bias (ROB)
	Representativeness of exposed cohort	Ascertainment of exposure	Precision of defining exposure	Exposure: prospectively or retrospectively	Outcome not present at beginning of study	Total score	Assessment of outcome	Was follow-up long enough?	Adequacy of follow-up	Total score	
Wagaskar et al., 2022	*	*	*	-	*	4	*	*	*	3	Low
Chau et al., 2022	*	*	*	*	*	5	*	*	*	3	Low
Ho Hogan et al., 2022	*	*	*	-	*	4	*	*	*	3	Low
Frisbie et al., 2022	*	*	*	-	*	4	*	*	*	3	Low
Girometti et al., 2022	*	*	*	-	*	4	*	*	*	3	Low
Wei et al., 2022	*	*	*	-	*	4	*	*	*	3	Low
Pan et al., 2021	*	*	*	-	*	4	*	*	*	3	Low
Wei et al., 2021	-	*	*	-	*	3	*	*	*	3	Moderate
Ryoo et al., 2020	*	*	*	-	*	4	*	*	*	3	Low
Maggi et al., 2021	*	*	*	*	*	5	*	*	*	3	Low
Deniffel et al., 2020	*	*	*	-	*	4	*	*	*	3	Low
Pet Petersmann et al., 2021	*	*	*	-	*	4	*	*	*	3	Low
Püllen et al., 2019	*	*	*	-	*	4	*	*	*	3	Low

© 2024 Haj-Mirzaian A et al. *JAMA Network Open.*

eMethods 5. Details of the Quality Assessment With the QUADAS-2 Tool

Review questions:

- What are the independent risk factors of the clinically significant prostate cancer?
- How to combine PI-RADS and clinical parameters (e.g., PSA, age, prior negative biopsy, etc.) to optimize prostate biopsy decision planning by reducing the number of unnecessary biopsies in patients with suspected prostate cancer?

Patients:

Adult patients with suspected, but not confirmed, clinically significant prostate cancer (i.e., abnormal PSA, etc.).

Index tests:

- Pre-biopsy bp-/mp-MRI with PI-RADS.
- Pre-biopsy clinical parameters (e.g., PSA, age, prior negative biopsy, etc.).

Reference standard:

Prostate biopsy (ultrasound- or MRI-guided systemic and/or targeted biopsy in PIRADS ≥ 3 lesions).

Domains:

Patient selection

1) Was a consecutive or random sample of patients enrolled?
2) Was a case-control design avoided?
3) Did the study avoid inappropriate inclusions or exclusions (e.g., including clinically significant prostate cancer)?
    ROB: Could the selection of patients have introduced bias?
    Applicability concerns: Are there concerns that the included patients do not match the review question?

Index test

1) Were the index test results interpreted without knowledge of and/or before the results of the reference standard?
2) Appropriate index test performed in all patients?
    ROB: Could the conduct or interpretation of the index test have introduced bias?
    Applicability concerns: Are there concerns that the index test, its conduct, or interpretation differ from the review question?

© 2024 Haj-Mirzaian A et al. *JAMA Network Open.*

Reference standard

1) Is the reference standard likely to correctly classify the target condition?

ROB: Could the reference standard, its conduct, or its interpretation have introduced bias?

Applicability concerns: Are there concerns that the target condition as defined by the reference standard does not match the review question?

Following and timing

1) Was there an appropriate interval between index tests and reference standard?
2) Did all patients receive a reference standard?
3) Did all patients receive the same reference standard?
4) Were all patients included in the analysis?

ROB: Could the patient flow have introduced bias?

Categorizing ROB based on the QUADAS-2 tool:

- Low ROB: low or less than two unclear risk on all domains relating to bias or applicability.
- Moderate ROB: two unclear risks on all domains relating to bias or applicability.
- High ROB: at least one high or more than two unclear risk on all domains relating to bias or applicability.

© 2024 Haj-Mirzaian A et al. *JAMA Network Open.*

eTable 2. Results of the Quality Assessment With the QUADAS-2 Tool

Study	Risk of bias: Patient selection	Risk of bias: Index test	Risk of bias: Reference standard	Risk of bias: Flow & timing	Applicability concerns: Patient selection	Applicability concerns: Index test	Applicability concerns: Reference standard
Bittencourt et al., 2022	☺	☺	☺	☺	☺	☺	☺
Wang et al., 2021	☺	☺	☺	☺	☺	☺	☺
Lendínez-Cano et al., 2021	☺	☺	☺	☺	☺	☺	☺
Sonmez et al., 2021	☺	☺	☺	☺	☺	☺	☺
Keck et al., 2021	☺	☺	☺	☺	☺	☺	☺
Deniffel et al., 2021	☺	☺	☺	☺	☺	☺	☺
Tosun and Uslu, 2021	?	☺	☺	☺	☺	☺	☺
Liang et al., 2021	☺	☺	☺	☺	☺	☺	☺
Fan et al., 2021	☺	☺	☺	☺	☺	☺	☺
Noh et al., 2020	?	☺	☺	☺	☺	☺	☺
Apfelbeck et al., 2020	?	☺	☺	☺	☺	☺	☺
Morote et al., 2020	☺	☺	☺	☺	☺	☺	☺
Falagario et al., 2020	☺	☺	☺	☺	☺	☺	☺
Sokhi et al., 2020	☺	☺	☺	☺	☺	☺	☺
Anastay et al., 2020	☺	☺	☺	☺	☺	☺	☺
Sonmez et al., 2020	☺	☺	☺	☺	☺	☺	☺
Kim et al., 2020	?	☺	☺	☺	☺	☺	☺
Busetto et al., 2020	☺	☺	☺	☺	☺	☺	☺
Stevens et al., 2020	☺	☺	☺	☺	☺	☺	☺
Wei et al., 2020	☺	☺	?	☺	☺	☺	☺
Al Hussein Al Awamlh et al., 2020	☺	☺	☺	☺	☺	☺	☺
Han et al., 2020	?	☺	☺	☺	☺	☺	☺

© 2024 Haj-Mirzaian A et al. *JAMA Network Open.*

He et al., 2019	☺	☺	☺	☺	☺	☺	☺
Borque-Fernando et al., 2019	?	☺	☺	☺	☺	☺	☺
Hsieh et al., 2019	☺	☺	☺	☺	☺	☺	☺
Lu, Zhang, Chen, et al., 2019	☺	☺	☺	☺	☺	☺	☺
Boesen, Nørgaard, et al., 2019	☺	☺	☺	☺	☺	☺	☺
Lu, Zhang, Yao, et al., 2019	?	☺	☺	☺	☺	☺	☺
Boesen, Thomsen, et al., 2019	☺	☺	☺	☺	☺	☺	☺
Bhat et al., 2018	☺	☺	☺	☺	☺	☺	☺
Kim et al., 2018	☺	☺	☺	☺	☺	☺	☺
Cuocolo et al., 2018	☺	☺	☺	☺	☺	☺	☺
Hansen et al., 2017	☺	☺	☺	☺	☺	☺	☺
Radtke et al., 2017	☺	☺	☺	☺	☺	☺	☺
Distler et al., 2017	☺	☺	☺	☺	☺	☺	☺
van Leeuwen et al., 2017	?	☺	☺	☺	☺	☺	☺
Washino et al., 2016	☺	☺	☺	☺	☺	☺	☺
Mehralivand et al., 2018	?	☺	☺	☺	☺	☺	☺
Alberts et al., 2018	☺	☺	☺	☺	☺	☺	☺
Sakaguchi et al., 2021	☺	☺	☺	☺	☺	☺	☺
Liu et al., 2021	☺	☺	☺	☺	☺	☺	☺
Zhang et al., 2019	☺	☺	☺	☺	☺	☺	☺
Görtz et al., 2019	☺	☺	☺	☺	☺	☺	☺
Saba et al., 2019	☺	☺	☺	☺	☺	☺	☺
Radtke et al., 2019	☺	☺	☺	☺	☺	☺	☺
Záleský et al., 2019	?	☺	☺	☺	☺	☺	☺
Punnen et al., 2018	?	☺	☺	☺	☺	☺	☺
Truong et al., 2017	☺	☺	☺	☺	☺	☺	☺
Tan et al., 2017	☺	☺	☺	☺	☺	☺	☺
Hansen et al., 2016	☺	☺	☺	☺	☺	☺	☺
Thompson et al., 2015	?	☺	☺	☺	☺	☺	☺
Zhu et al., 2022	☺	☺	☺	☺	☺	☺	☺

© 2024 Haj-Mirzaian A et al. *JAMA Network Open.*

Campistol et al., 2022	☺	☺	☺	☺	☺	☺	☺
Morote, Borque-Fernando, et al., 2022	☺	☺	☺	☺	☺	☺	☺
Gan et al., 2022	☺	☺	☺	☺	☺	☺	☺
Zhang et al., 2022	☺	☺	☺	☺	☺	☺	☺
Zhou et al., 2022	☺	☺	☺	☺	☺	☺	☺
van Riel et al., 2022	☺	☺	☺	☺	☺	☺	☺
Morote, Campistol, et al., 2022	☺	☺	☺	☺	☺	☺	☺
Wagaskar et al., 2022	☺	☺	☺	☺	☺	☺	☺
Chau et al., 2022	☺	☺	☺	☺	☺	☺	☺
Ho Hogan et al., 2022	☺	☺	☺	☺	☺	☺	☺
Frisbie et al., 2022	☺	☺	☺	☺	☺	☺	☺
Girometti et al., 2022	☺	☺	☺	☺	☺	☺	☺
Wei et al., 2022	☺	☺	☺	☺	☺	☺	☺
Pan et al., 2021	☺	☺	☺	☺	☺	☺	☺
Wei et al., 2021	?	☺	☺	☺	☺	☺	☺
Ryoo et al., 2020	☺	☺	☺	☺	☺	☺	☺
Maggi et al., 2021	☺	☺	☺	☺	☺	☺	☺
Deniffel et al., 2020	☺	☺	☺	☺	☺	☺	☺
Pet Petersmann et al., 2021	☺	☺	☺	☺	☺	☺	☺
Püllen et al., 2019	☺	☺	☺	☺	☺	☺	☺

☺ Low ? Unclear ☹ High

© 2024 Haj-Mirzaian A et al. *JAMA Network Open.*

eFigure 1. Pooled Results of the Quality Assessment With the QUADAS-2 Tool

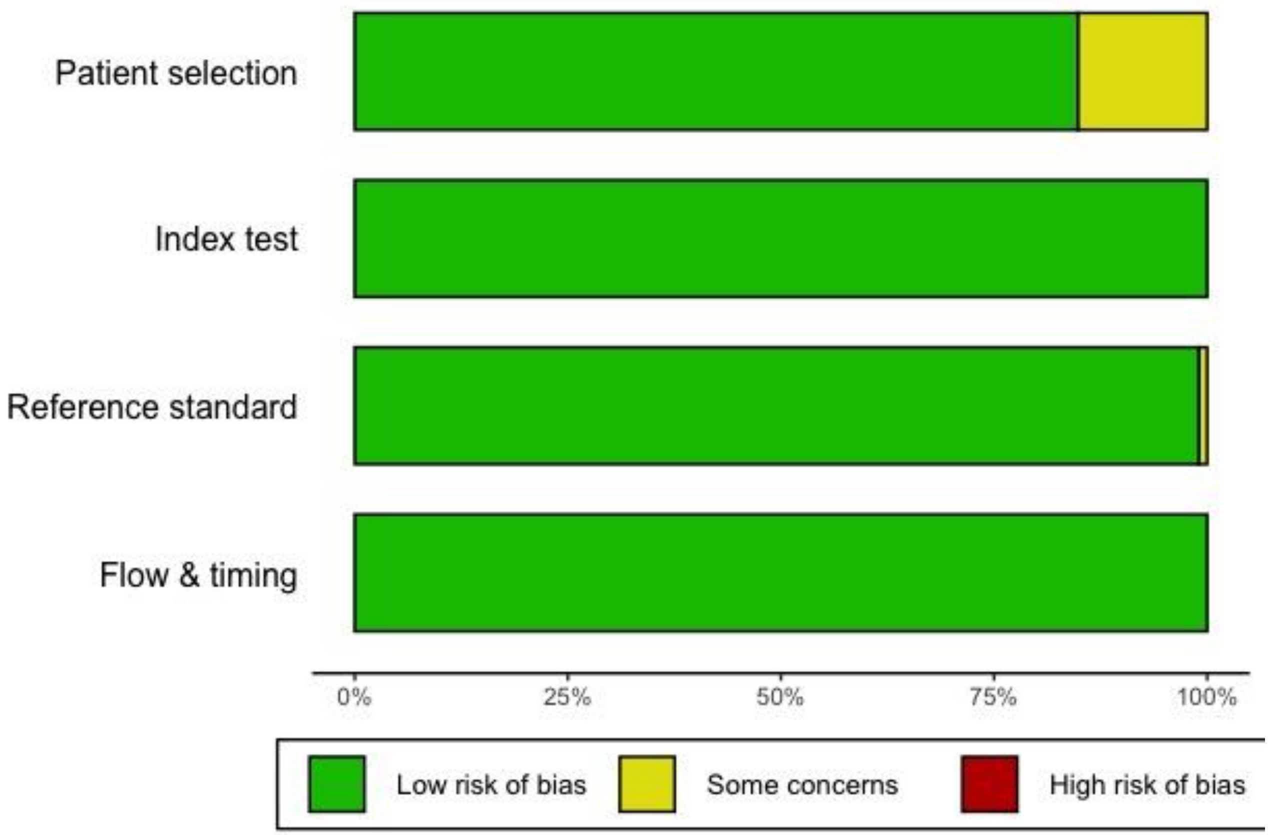

© 2024 Haj-Mirzaian A et al. *JAMA Network Open.*

eMethods 6. Methodology and Analytic Approach: Multimodel Inference Method

The detailed analytic approach used to investigate and define the most important risk factors for csPCa is outlined below:

1. Study Inclusion Criteria:
    a. Included studies that combined clinical factors with PI-RADS and reported the added value in reducing unnecessary biopsies.
    b. Explored previously published narrative/systematic reviews on this topic to create a list of relevant clinical parameters, including but not limited to total PSA, free PSA, free/total PSA ratio, PSAD, age, race, ethnicity, BMI, family history of prostate cancer, DRE, history of prior biopsy, etc.
2. Data Extraction:
    a. Extracted the rate of csPCa and the overall value of each clinical/imaging factor from each study.
3. Univariate Analysis:
    a. Assessed the association between each factor and the rate of csPCa using univariate analysis.
4. Multi-Model Inference Method:
    a. Utilized the multi-model inference method, which generates all possible combinations of variables in sets of multiple variable meta-regression models. This method identifies the best-fit combination of risk factors and determines the most important risk factors of csPCa.
    b. Variables were selected based on a clearly defined scientific rationale (known risk factors of csPCa), a statistical criterion (variables that significantly explained the variability in the data on univariate analysis) and ensured there was no co-linearity.
    c. The factor(s) showing a significant association with csPCa on the multi-model inference method were considered as the main risk factors of csPCa and were chosen for further evaluation in the stepwise approach analysis.
5. Stepwise Approach Analysis:
    a. Selected studies that assessed the yield of combining PI-RADS and PSAD by reporting patient-level data.
    b. Created 2 x 2 tables for the findings of each study, outlining true-positive, true-negative, false-positive, and false-negative test results for each combining PI-RADS + PSAD strategy.
    c. Calculated pooled diagnostic parameters for each stepwise strategy using a generalized linear mixed-effects model."

© 2024 Haj-Mirzaian A et al. *JAMA Network Open.*

eTable 3. Study Design of the Included Studies

Study			Patient selection			Index tests		Reference standard			
	Design	Enrollment	Inclusion criteria	Exclusion criteria	Study population	MRI*	Clinical data	Biopsy	Pathology	Interval	Included PI-RADS
Bittencourt et al., 2022	Retro.	Cons.	Suspected prostate cancer – Biopsy naïve men; Suspicion of PCa was defined as elevated PSA serum level above 2.5 ng/mL, suspicious DRE (cT ≥ 2), or positive family history of aggressive PCa.	Referred patients with previous treatment, with non-PI-RADS-compliant MRI, significant MRI artifacts, incomplete biopsy procedure, or within > 6-month interval between MRI and biopsy.	Subjects without but suspected to have csPCa - Moderate risk	1.5 & 3T mp	PSAD, DRE, FHx	Systematic biopsy (12 cores per patient) and targeted biopsy (2–4 cores per target, up to 4 targets per patient).	ISUP	Subsequent <6 months	All
Lendínez-Cano et al., 2021	Pros.	Cons.	Suspected prostate cancer - Biopsy-naïve patients; Normal DRE; Total PSA between 3 and 10 ng/ml; %free PSA ≤ 20%; Age 50–75 years; and at least two consecutive measurements of PSA levels within 2–4 weeks before the inclusion.	A prior diagnosis of PCa, medical therapy known to affect serum PSA levels within six months before urine sample collection (such as finasteride, dutasteride, etc.), invasive treatment of BPH within six months of urine sample collection, inability to complete study procedures (pacemakers that prevent MRI, absence of an anus that would make DRE and prostate massage impossible), or alterations that would compromise the ability to grant consent.	Subjects without but suspected to have csPCa – Low to moderate risk	3T mp	Risk score mode – select MDx, ERSPC3-RC	Systematic 12-core trans-rectal biopsy and a targeted biopsy (maximum of four cores) was added when a PIRADS > 2 lesion was detected on MRI.	ISUP	Subsequent	All
Sonmez et al., 2021	Retro.	NA	Suspected prostate cancer - Age 45-75 years, PSA 2.5–10 ng/mL and/or a suspicious DRE, and PI-RADS ≥3, with or without previous negative prostate biopsy history.	Patients with other malignancies, a known infection, hematological diseases, and clinical conditions causing platelet disorders. Moreover, patients with a	Subjects without but suspected to have csPCa - Moderate risk	1.5T mp	SII (symstectimc immune inflammation	All the patients initially underwent 12-lead standard transrectal prostate biopsy. Additional 2-5 cores were obtained from suspicious lesions using mpMRI-targeted biopsy.	ISUP	Subsequent	PI-RADS ≥3

© 2024 Haj-Mirzaian A et al. *JAMA Network Open.*

				histopathological diagnosis of atypical small acinar proliferation, prostatic intraepithelial neoplasia or patients who already had a diagnosis of PCa (AS).			index) + PIRADS				
Keck et al., 2021	Pros.	Cons.	Suspected prostate cancer - Prostate MRI and biopsy in suspected patients.	Confirmed PCa.	Subjects without but suspected to have csPCa - Moderate risk	3T mp	Age, PSA, prior Bx	A combined targeted and systematic 12-core transrectal MRI-ultrasound fusion-guided biopsy.	ISUP	Subsequent	All
Deniffel et al., 2021	Retro.	Cons.	Suspected prostate cancer - Completed mpMRI and MRI-targeted biopsy without prior PCa diagnosis.	Insufficient image quality.	Subjects without but suspected to have csPCa - Moderate risk	3T mp	Multiple risk score models	The addition of a minimum of 12 systematic biopsy cores and the number of targeted cores, with at least two targeted cores per lesion.	ISUP	Subsequent	All
Tosun and Uslu, 2021	Retro.	NA	Suspected prostate cancer - Underwent prebiopsy mpMRI, had serum PSA and available histopathological data.	History of prostate surgery, no prebiopsy MRI and with PSA concentrations measured at another hospital.	Subjects without but suspected to have csPCa - Moderate risk	3T mp	PSA, PSAD, age	Targeted biopsy together with systematic biopsy. Two biopsy cores of each targeted lesion. A 12-core systematic extended biopsy.	ISUP	Subsequent	All
Liang et al., 2021	Retro.	Cons.	Suspected prostate cancer - Negative prebiopsy bpMRI, defined as PI-RADS score less than 3.	Per inclusion.	Subjects without but suspected to have csPCa – Low risk	3T bp	PSAD, age, PV, DRE, PSA	Systematic 14-core biopsy [no focal lesion – all PI-RADS 1-2 or no focal lesion].	ISUP	Subsequent	PI-RADS ≤2
Fan et al., 2021	Pros.	NA	Suspected prostate cancer - Patients with at least one PI-RADS ≥ 3 lesions on mpMRI who underwent an MRI-TRUS fusion-targeted biopsy. All patients had PSA ≥ 4 ng/ml and/or a suspicious DRE with or without a	Acute prostatitis or urinary tract infections, a prior history of PCa, the use of 5-α reductase inhibitors within the previous three months and having undergone transurethral resection of the prostate.	Subjects without but suspected to have csPCa - Moderate to high risk	3T mp	PSA, PSAD, F PSA, PHI	MRI-targeted biopsy was performed under TRUS-guidance. A systematic 12-core biopsy was subsequently performed after the targeted biopsy.	ISUP	Subsequent	PI-RADS ≥3

© 2024 Haj-Mirzaian A et al. *JAMA Network Open.*

			previous negative prostate biopsy.								
Noh et al., 2020	Retro.	NA	Suspected prostate cancer - PSA ≥ 4.0 ng/mL and/or abnormal DRE who underwent prebiopsy bpMRI-ultrasound fusion transperineal targeted and systematic biopsies in the same session.	Per inclusion	Subjects without but suspected to have csPCa - Moderate risk	3T bp	Age, PSAD, PSA	Fusion trans-perineal targeted and systematic biopsies.	ISUP	Subsequent	All
Apfelbeck et al., 2020	Retro.	NA	Suspected prostate cancer - Underwent mpMRI and prostate biopsy. PI-RADS 4 or 5.	Per inclusion.	Subjects without but suspected to have csPCa - High risk	NA mp	Age, PSA, PV, PSAD, prior bx	Targeted bx for focal lesions on MRI. In every patient an additional systematic biopsy was performed.	ISUP	Subsequent	PI-RADS ≥4
Falagario et al., 2020	Pros.	Cons.	Suspected prostate cancer - Underwent prostate biopsies and MRI. PSA value of <20 ng/ml.	Prior diagnosis of PCa.	Subjects without but suspected to have csPCa - Moderate risk	1.5 & 3T bp	PSAD, PSA, Prior bx	Negative MRI underwent systematic biopsy using a 12-core template. Additionally, in patients with positive MRI, two to four extra target cores were taken from each suspicious lesion.	ISUP	Subsequent	All
Sokhi et al., 2020	Retro.	NA	Suspected prostate cancer - Including abnormal DRE and/or elevated PSA, mpMRI as an initial diagnostic test and then biopsy. All biopsy naïve or previous negative biopsy.	Per inclusion.	Subjects without but suspected to have csPCa – Moderate risk	3T mp	PSA, PSAD, DRE	In positive MRI (PI-RADS 3-5), targeted biopsies were performed; systematic non-targeted biopsies of non-suspicious MRIs were also obtained. In negative MRI, systematic prostate biopsies were performed.	ISUP	Subsequent	All
Anastay et al., 2020	Retro.	Cons.	Suspected prostate cancer - Underwent prostatic transrectal ultrasound-guided biopsy. Included all patients with negative prebiopsy mpMRI defined as PI-RADS ≤ 2.	History of radiotherapy, prostatectomy, and high-intensity-focused ultrasound or metastatic disease.	Subjects without but suspected to have csPCa – Low risk	1.5T mp	PSAD	TRUSGB was systematically performed regardless of MRI findings (no targeted as all included subjects had negative MRI).	ISUP	Subsequent	PI-RADS ≤2

© 2024 Haj-Mirzaian A et al. *JAMA Network Open.*

Sonmez et al., 2020	Pros.	NA	Suspected prostate cancer - Underwent mpMRI-guided fusion prostate biopsy due to elevated PSA or a suspicious DRE. Inclusion criteria were PSA < 10 ng/mL and detection of a PI-RADS 3 lesion on prebiopsy mpMRI alone.	Atypical small acinar proliferation and high-grade prostatic intraepithelial neoplasia. Patients who already had a diagnosis of PCa (active surveillance).	Subjects without but suspected to have csPCa – Low risk	3T mp	PSA DRE, F/T PSA, PSAD	A total of 2-5 biopsy cores were obtained from each PI-RADS 3 lesion marked on the mpMRI. Following the completion of the target biopsy, standard 12-core biopsy was performed in each patient.	ISUP	Subsequent < 6 months	PI-RADS =3
Kim et al., 2020	Retro.	NA	Suspected prostate cancer - mpMRI within 6 months prior to the biopsy, in men received a consequent prostate biopsy. 5% of patients with prior positive prostate biopsy, not clearly mentioned it was GS 6.	Prostate sarcoma, extra-renal Wilims' tumor, and PI-RADS 1–2 lesions.	Subjects without but suspected to have csPCa - Moderate to high risk	3T mp	Age, PSAD, PSA, PV, prior bx	12 cores systemic biopsy were taken from all patients. Additionally, 2–4 MRI TB cores for suspicious or equivocal lesions were obtained.	ISUP	Subsequent <6 months	PI-RADS ≥3
Busetto et al., 2020	Pros.	Cons.	Suspected prostate cancer - Men who were scheduled for initial prostate biopsy, based on elevated total PSA level (>3 ng/ml) or abnormal DRE.	History of PCa or other neoplasm under active treatments, medical therapies known to affect PSA and the prostate gland, invasive treatments for BPH, prior prostatic biopsy.	Subjects without but suspected to have csPCa - Moderate risk	3T mp	Select MDx, PSA, PSAD	12 systematic cores were obtained. In cases of PI-RADS score 3–5 at mpMRI, additional targeted samples (2 cores per lesion) were obtained.	ISUP	Subsequent	All
Stevens et al., 2020	Retro.	NA	Suspected prostate cancer - Undergone MRI and prostate biopsies. No previous diagnosis of PCa and those with GG group 1 PCa undergoing AS.	Underwent MRI for indications other than initial detection and AS, patients who had undergone a biopsy procedure more than 6 months after MRI, and patients without serum PSA values or adequate gland volume measurements recorded in the registry.	Subjects without but suspected to have csPCa - Moderate risk	3T bp	PSA, PSAD	12-core systematic biopsy procedure in patients who had at least 1 lesion with a PI-RADS score of 3 or higher. In patients with no lesions of interest on MRI (PI-RADS 1 or 2), a systematic biopsy procedure was performed.	ISUP	Subsequent <6 months	All

© 2024 Haj-Mirzaian A et al. *JAMA Network Open.*

Wei et al., 2020	Retro.	NA	Suspected prostate cancer - Suspected PCa based on increased serum PSA levels of 4–10 ng/mL. All patients who enrolled underwent prostate bp-MRI examination and a systematic TRUS- guided prostate biopsy before a pathological diagnosis was finally obtained.	If they met one of the following criteria: underwent biopsy or other therapies before MRI examination, PSA level <4 ng/mL or >10 ng/mL, MR images were not satisfactory (obvious artifacts from patient movements and other conditions, such as hip replacement), or other prostate pathology independent of prostate adenocarcinoma.	Subjects without but suspected to have csPCa – Low to moderate risk	3T bp	PSAD, PV	All patients were subject to a 10-core systematic. An additional 2 or 3 targeted cores were added for suspicious areas detected by TRUS.	ISUP	Subsequent	All
Al Hussein Al Awamlh et al., 2020	Pros.	NA	Suspected prostate cancer - Only those with a category 3 index lesion on mpMRI using PI-RADS classification.	Subjects who had a prior biopsy.	Subjects without but suspected to have csPCa – Low risk	3T mp	Age, PSA	Following targeted biopsies, a 12-14 core systematic (template) biopsy was performed.	ISUP	Subsequent	PI-RADS =3
Han et al., 2020	Retro.	Cons.	Suspected prostate cancer - Underwent a prostate mpMRI examination.	No histopathological results or clinical information, previous treatment for PCa before mpMRI examination, PSA serum level outside of the 4~10 ng/mL rang, severe susceptibility artifacts or respiratory motion artifacts on MRI images, no TRUS-guided biopsy performed within 3 months after MRI examination, no follow-up or follow-up for less than 12 months if no csPCa was found after the initial biopsy.	Subjects without but suspected to have csPCa – Low to moderate risk	3T Both bp & mp (mp data was used)	PSAD	Systematic or systematic plus cognitive-targeted biopsy (based on the lesion on MRI).	ISUP	Subsequent <3 months	All
He et al., 2019	Retro.	Cons.	Suspected prostate cancer - Biopsy- naïve or had previous negative biopsy.	PSA levels above 50 ng/ml and missing data.	Subjects without but suspected to	3T mp	PSA, PV, age, obesity	216 patients underwent cognitive MRI-guided TB combined with systematic biopsy, while 26	ISUP	Subsequent	All

© 2024 Haj-Mirzaian A et al. *JAMA Network Open.*

					have csPCa - Moderate risk			underwent only systematic biopsy because the large volume or multifocality of suspicious lesions located in the region of the systematic biopsy cores were highly unlikely to be missed. In addition, 143 men with PI-RADS scores less than 3 underwent only systematic biopsy.			
Borque-Fernando et al., 2019	Pros.	NA	Suspected prostate cancer - Suspected PCa on the basis of PSA level above 4 ng/mL or abnormal finding in DRE. All patients underwent a mpMRI prior to biopsy.	Per inclusion.	Subjects without but suspected to have csPCa - Moderate risk	3T mp	Age, PSAD, DRE, PNBx	12-core systematic transrectal ultrasound-guided PBx, with 2 extra cores by cognitive fusion from each suspicious area.	ISUP	Subsequent	All
Hsieh et al., 2019	Pros.	NA	Suspected prostate cancer - More than 40 years and underwent prostate biopsy for suspicious PC due to elevated serum PSA level (PSA > 4 ng/mL) and/or abnormal findings on DRE.	Histories of PCa, bacterial prostatitis in 3 months before biopsy, use of 5-alpha reductase inhibitors, or inability/unwillingness to sign informed consent.	Subjects without but suspected to have csPCa - Moderate risk	3T mp	PHI	PI-RADS ≥ 3 as the target lesions. Then, cognitive registration targeted biopsy was performed, followed by systematic biopsy.	ISUP	Subsequent	All
Lu, Zhang, Chen, et al., 2019	Retro.	NA	Suspected prostate cancer - Patients underwent prostate MRI and prostate biopsy.	Prior prostate mpMRI, patients previously accepted treatment (drug treatment, biopsies, or surgical therapy); Serum examinations were missing or performed after treatment; The time interval between MRI and the following TRUS-guided 12-core prostate biopsy was more than 2 weeks.	Subjects without but suspected to have csPCa - Moderate risk	3T mp	PSAD, PSA	12+X-G needle under ultrasound-guidance. TRUS-guided biopsy combined TRUS- guided targeted biopsy with cognitive MRI fusion-guided targeted biopsy.	ISUP	Subsequent <2 weeks	All
Boesen, Nørgaard, et al., 2019	Pros.	NA	Suspected prostate cancer - Biopsy-naïve with clinical suspicion of localized PCa	Prior prostate biopsies, prior prostate MRI, PSA >20 ng/ml, DRE with suspicion	Subjects without but suspected to	1.5T bp	PSAD	Systematic 10-core TRUS biopsy followed by additional TBx of any suspicious lesions (bpMRI score ≥3).	ISUP	Subsequent	All

© 2024 Haj-Mirzaian A et al. *JAMA Network Open.*

			(PSA <20ng/ml and DRE <cT3).	of locally advanced PCa (>cT3), evidence of acute urinary tract infections, acute prostatitis, general contraindications for MRI, and prior hip replacement surgery or other metallic implants in the pelvic area.	have csPCa - Moderate risk						
Lu, Zhang, Yao, et al., 2019	Retro.	NA	Suspected prostate cancer - All patients underwent standardized prostate mpMRI before drug, biopsy or surgical therapy, serum examination was performed before treatment, and transrectal ultrasonography and 12-core prostate biopsy with pathological results were performed in the next 2 weeks after mpMRI.	Per inclusion.	Subjects without but suspected to have csPCa - Moderate risk	3T mp	PSAD, F/T PSA, PSA	12 + X needle under ultrasound guidance (six in the peripheral zone, six in the transitional zone, X in the suspicious zone). TRUS-guided biopsy was combined with TRUS-guided targeted biopsy and cognitive MRI fusion-guided targeted biopsy.	ISUP	Subsequent <2 weeks	All
Boesen, Thomsen, et al., 2019	Pros.	NA	Suspected prostate cancer - All men to have clinical suspicion of PCa (PSA >4ng/mL and/or suspicious DRE results) warranting diagnostic prostate biopsies.	Prior prostate biopsies, prior prostate MRI, evidence of acute urinary tract infection, prostatitis, general contra- indications for MRI (e.g., claustrophobia, pacemaker, metal implants), and prior hip replacement surgery or other metallic implants in the pelvic area.	Subjects without but suspected to have csPCa - Moderate risk	3T bp	Age, PSAD, DRE	Standard 10-core TRUS bx plus additional targeted biopsies (1–2 cores/lesion) of any bpMRI suspicious lesion (score 3–5) by one of two operators.	ISUP	Subsequent	All
Bhat et al., 2018	Retro.	NA	Suspected prostate cancer - Men who received prostate mpMRI, including biopsy naïve or with previous negative biopsy.	Known PCa prior to biopsy.	Subjects without but suspected to have csPCa - Moderate risk	3T mp	PSA, PSAD	Fusion MRI-targeted biopsy as well as standard 12-core systematic biopsy.	ISUP	Subsequent	All

© 2024 Haj-Mirzaian A et al. *JAMA Network Open.*

Kim et al., 2018	Retro.	NA	Suspected prostate cancer - Patients who underwent mpMRI of the prostate and subsequent prostate biopsy. PI-RADS 3. Biopsy naïve or with previous negative biopsy.	Previous prostate surgery. Inadequate medical records or MRI studies. Patients on anti-androgen therapy at the time of biopsy. Did not undergo biopsy after MRI.	Subjects without but suspected to have csPCa – Low risk	3T mp	Age, PSAD	12 cores were taken in all patients. Two cognitive fusion-targeted biopsy cores were added for each lesion in patients with suspicious or equivocal lesions.	ISUP	Subsequent ~25 days	PI-RADS =3
Cuocolo et al., 2018	Retro.	Cons.	Suspected prostate cancer - Clinical suspicion of PCa, derived from either elevated PSA or abnormal DRE.	Absence of TRUS-biopsy within 30 days following the MR exam, previous prostate biopsy, MR images affected by artifacts, incomplete MR exam.	Subjects without but suspected to have csPCa - Moderate risk	3T bp	PSAD	Standard 12-core TRUS-guided biopsy. Additionally, cognitive-targeted biopsy was used after careful review of the patient's bp-MR exam.	ISUP	Subsequent ~1 month	All
Hansen et al., 2017	Pros.	Cons.	Suspected prostate cancer - All patients with first suspicion of prostate cancer without previous negative biopsies or previous diagnosis or treatment of PCa.	Patients aged >79 years and with a PSA level >30 ng/mL.	Subjects without but suspected to have csPCa - Moderate risk	1.5 & 3T mp	PSAD, PSA	Systematic trans-perineal biopsies according to the Ginsburg protocol. In patients with PI-RADS 3–5 MRI lesions, at least two biopsy cores were taken from each lesion before the systematic biopsies.	ISUP	Subsequent	All
Radtke et al., 2017	Pros.	Cons.	Suspected prostate cancer - mpMRI with PI-RADS scoring and fusion biopsy.	Men under AS and men who had missing data.	Subjects without but suspected to have csPCa - Moderate risk	3T mp	ERSPC, PSA, PV, DRE, age	Underwent transperineal fusion targeted biopsy of MRI-suspicious lesions first (2–5 cores per lesion) and then systemic biopsy adjusted to PV.	ISUP	Subsequent ~1 day	All
Distler et al., 2017	Pros.	Cons.	Suspected prostate cancer - PSA greater than 4.0 ng/ml and/or suspicious DRE, and who were biopsy naïve or had undergone a previous negative biopsy.	Per inclusion	Subjects without but suspected to have csPCa - Moderate risk	3T NA mp	PSAD, prior bx, DRE	Trans-perineal MRI targeted-TRUS fusion biopsy of MRI suspicious lesions and then trans-perineal systematic biopsy with a median of 24 cores. Men with no lesion only underwent trans-perineal systematic MRI-TRUS fusion biopsy.	ISUP	Subsequent	All
van Leeuwen et al., 2017	Pros.	Cons.	Suspected prostate cancer - Men were aged >40 years, planned for biopsy for abnormal PSA level or DRE, and had a life-expectancy of >10 years.	Per inclusion.	Subjects without but suspected to have csPCa - Moderate risk	1.5 & 3T mp	PSA, DRE, PV, age, previous bx	Trans-perineal mapping biopsies from 18 template locations. Urologists reviewed the mpMRI report and images and collected two additional targeted cores from all ROI potentially under sampled by	ISUP	Subsequent	All

© 2024 Haj-Mirzaian A et al. *JAMA Network Open.*

			mpMRI was performed before prostate biopsy.					template biopsy using MRI/TRUS-fusion biopsy or cognitive.			
Washino et al., 2016	Retro.	NA	Suspected prostate cancer - Underwent mpMRI before their first prostate biopsy.	Previous prostate surgical intervention, duration between mpMRI and biopsy of >6 months, <14 biopsy cores taken, and taking a 5a-reductase inhibitor or anti- androgen at the time of biopsy.	Subjects without but suspected to have csPCa - Moderate risk	1.5 & 3T bp	PSAD, PSA	14 cores for the systematic biopsies in all patients. Two cognitive fusion-targeted biopsy cores were added for each lesion in patients who had suspicious or equivocal lesions on mpMRI.	ISUP	Subsequent <6 months	All
Mehralivand et al., 2018	Pros.	Cons.	Suspected prostate cancer - Underwent mpMRI and prostate biopsy. Patients with elevated serum PSA or abnormal DRE and at least 1 lesion detected on results of multiparametric MRI were included.	Negative MRI results, nondiagnostic MRI results owing to artifacts (eg, excess patient motion or metallic prosthesis–related artifacts), prior treatment for prostate cancer (radical prostatectomy, external beam radio- therapy, brachytherapy, focal therapy, or androgen deprivation therapy), or other forms of local treatment (transurethral resection of prostate or bladder instillation therapy).	Subjects without but suspected to have csPCa - Moderate risk	3T mp	Age, race, DRE, PNB, PV, PSAD	Each lesion was biopsied with at least 2 biopsy cores per lesion. After obtaining the targeted biopsies, a 12-core systematic biopsy was performed.	ISUP	Subsequent	All
Alberts et al., 2018	Pros.	Cons.	Suspected prostate cancer - Men with a clinical suspicion of PCa (no prior PCa diagnosis), who received mpMRI and subsequent TRUS-biopsy and/or targeted biopsy.	Per inclusion.	Subjects without but suspected to have csPCa - Moderate risk	3T mp	ERSPC, age, DRE	MRI-TBx was performed for each PI-RADS ≥3 lesion, and systemic biopsy.	ISUP	Subsequent	All
Sakaguchi et al., 2021	Retro.	NA	Suspected prostate cancer - Indications for biopsy were high PSA level (≥ 4.0 ng/ml), abnormal DRE or lesions suggestive of	Previous prostate surgery, previous diagnosis of PCa or administration of 5-alpha-reductase inhibitors or anti-androgens, as	Subjects without but suspected to have csPCa -	1.5 & 3T bp	Age, PSA	Systematic trans-perineal and trans-rectal biopsy (8–14 cores). If one or more lesions suggestive of prostate cancer were detected on bpMRI (PI-RADS score ≥ 3), trans-perineal	ISUP	Subsequent	All

© 2024 Haj-Mirzaian A et al. *JAMA Network Open.*

			prostate cancer on bpMRI.	agents that affect PSA values.	Moderate risk			cognitive targeted biopsies were added for each lesion (2–4 cores of each lesion).			
Liu et al., 2021	Retro.	Cons.	Suspected prostate cancer - Men aged greater than 18 years who underwent consecutive TRUS-guided systematic prostate biopsy, had negative mpMRI score <3 points evaluated by PI-RADS, negative transrectal ultrasound, and negative DRE. Men with a PSA level ≤30ng/ml.	Patients with prior PCa, surgical treatment for BPH, no MRI data due to contraindications (metal implants, etc.), evidence of acute urinary tract infections, acute prostatitis, and patients taking a 5a-reductase inhibitor or anti-androgen at the time of biopsy or during the previous 6 months.	Subjects without but suspected to have csPCa – Low risk	3T mp	PNB, age, PSAD	Systematic 12-core prostate biopsy was performed under local an-esthesia, according to international guidelines	ISUP	Subsequent <3 months.	PI-RADS ≤2
Zhang et al., 2019	Pros.	NA	Suspected prostate cancer - Transrectal ultrasound-guided 24-core biopsy and mpMRI. Patients who were biopsy-naïve and had a maximum PI-RADS score of 3.	Inappropriate MRI sequences or who had undergone surgery for benign prostatic hyperplasia.	Subjects without but suspected to have csPCa – Low risk	3T mp	Age, PSA, PSAD, PV	Systematic TRUS-Bx with 24 needle cores. The targeted lesions were sampled using 2 or 3 cores.	ISUP	Subsequent	PI-RADS =3
Görtz et al., 2019	Pros.	Cons.	Suspected prostate cancer - Biopsy-naïve and had suspicion of PCa with a PSA level >4 ng/ml and/or suspicious DRE. Underwent prostate MRI and biopsy. PI-RADS 3.	Taking 5a-reductase inhibitors.	Subjects without but suspected to have csPCa – Low risk	3T mp	Age, DRE, PSA, PSAD	Trans-perineal grid-directed biopsy with elastic software registration. TB of MRI-suspicious lesions was performed first (median of 5 cores per lesion), followed by systematic saturation biopsy.	ISUP	Subsequent	PI-RADS =3
Saba et al., 2019	Retro.	NA	Suspected prostate cancer - All men who underwent mpMRI and trans-perineal template saturation prostate biopsy with additional fusion guided targeted biopsy for suspicion of csPCa.	Insufficient MRIs and history of PCa.	Subjects without but suspected to have csPCa - Moderate risk	NA mp	Multiple risk score models	Systemic with additional fusion guided targeted biopsies (2 to 4 cores) were performed for each ROI classified as grade 3 or greater according to the PI-RADS.	ISUP	Subsequent	All

© 2024 Haj-Mirzaian A et al. *JAMA Network Open.*

Radtke et al., 2019	Pros.	Cons.	Suspected prostate cancer - Based on PSA or DRE. mpMRI with PI-RADS scoring and fusion-biopsy. All biopsy naïve.	Men under AS and men who had missing data.	Subjects without but suspected to have csPCa - Moderate risk	1.5 & 3T mp	PSA, PV, DRE, age, ERSPC RC3	MRI-suspicious lesions first (2–5 cores per lesion) and then standard systemic biopsy adjusted to PV.	ISUP	Subsequent	All
Záleský et al., 2019	Pros.	Cons.	Suspected prostate cancer - Age <80 years, elevated PSA, negative DRE, no coagulopathy.	Previous prostate surgery, inability to undergo mpMRI examination (metal implants, pace-maker present, chronic renal failure, claustrophobia).	Subjects without but suspected to have csPCa - Moderate risk	1.5T mp	PSAD, prior bx	MRI/TRUS fusion-targeted biopsies were performed on patients with PIRADS 3–5 lesions followed by a systematic trans-rectal biopsy. Fusion-targeted biopsies consisted of 1–3 cores from each MRI suspicious lesion PIRADS ≥3. Systematic transrectal biopsy consisted of 12 cores.	ISUP	Subsequent	All
Punnen et al., 2018	Retro.	Cons.	Suspected prostate cancer - Men who underwent mpMRI and a 4Kscore test for evaluation of prostate cancer. Most of the patients were referred for a biopsy due to an elevated PSA, while some were also referred due to abnormal DRE or mpMRI.	Per inclusion.	Subjects without but suspected to have csPCa - Moderate risk	3T mp	4K score, PSA, age, DRE, previous bx	Two cores taken from each target if a suspicious mpMRI visible target was seen (PIRADS 3 or higher). In addition, all men underwent a 12-core extended template biopsy.	ISUP	Subsequent < 6 months	All
Truong et al., 2017	Retro.	Cons.	Suspected prostate cancer - Patients with at least 1 prior negative biopsy session who underwent biopsy for abnormal mpMRI findings (i.e., a PI-RADS score of 3-5). The indication for performing mpMRI was continued clinical suspicion for clinically significant PCa.	Biopsy-naive or had an existing PCa diagnosis.	Subjects without but suspected to have csPCa - Moderate to high risk	3T mp	Age, PV, PSA	Targeted biopsy and concurrent 12- to 14-core systemic biopsy. At least 2 targeted biopsy cores were obtained for each visible region of interest on mpMRI.	ISUP	Subsequent	PI-RADS ≥3

© 2024 Haj-Mirzaian A et al. *JAMA Network Open.*

Tan et al., 2017	Pros.	Cons.	Suspected prostate cancer - Patients with at least one PI-RADS 3 or higher lesion on mpMRI. All patients had either a raised serum PSA and/or suspicious DRE and/or previous negative systematic TRUS prostate biopsy.	Patients who had undergone previous PCa treatment.	Subjects without but suspected to have csPCa - Moderate risk	3T mp	PSAD, PHI	Targeted lesions with real-time MRI-TRUS fusion biopsy. In the same session, a systematic 12-core biopsy.	ISUP	Subsequent ~48 days	PI-RADS ≥3
Hansen et al., 2016	Retro.	NA	Suspected prostate cancer - Underwent trans-perineal prostate biopsies.	Previous prostate cancer. Patients on AS for Gleason score 7 cancer. Patients on AS who were diagnosed with Gleason score 6 cancer before 2010, to ensure that the diagnostic Gleason grading for all included patients was done according to the 2005 ISUP criteria and insufficient data recording.	Subjects without but suspected to have csPCa - Moderate risk	1.5 & 3T mp	PSAD	24 systematic biopsies according to the Ginsburg protocol. In patients with PI-RADS 3–5 MRI lesions, two biopsy cores were taken from each lesion before the systematic biopsies.	ISUP	Subsequent	All
Thompson et al., 2015	Pros.	NA	Suspected prostate cancer - Men older than 40 years, scheduled to undergo biopsy for abnormal PSA or DRE, with a life expectancy greater than 10 years and no previous prostate MRI or biopsy.	Per inclusion.	Subjects without but suspected to have csPCa - Moderate risk	1.5 & 3T mp	PSA, DRE, PV, age	All participants underwent systemic biopsy (median of 30 cores). Urologists reviewed the MRI report and images and collected 2 additional targeted cores from all ROIs.	ISUP	Subsequent	All
Zhu et al., 2022	Retro.	NA	Suspected prostate cancer - Patients with PI-RADS<3 and DRE finding nodules or persistent PSA >4 ng/ml.	Patients with prior biopsy. Patients with a prior diagnosis of prostate cancer, and patients with incomplete clinical data.	Subjects without but suspected to have csPCa – Low risk	3T mp	PSAD	Systemic biopsy with targeted in PI-RADS 1-2	ISUP	Subsequent	PI-RADS ≤2
Morote, Borque-Fernando, et al., 2022	Retro.	NA	Suspected prostate cancer - Suspected PCa due to a serum PSA > 3.0 ng/mL or an abnormal DRE.	Men undergoing 5-α reductase inhibitor treatment due to symptomatic benign	Subjects without but suspected to have csPCa -	3T mp	PSAD	Underwent 2 to 4-core mpMRI-TRUS cognitive fusion-guided biopsies of suspicious lesions and 12-core TRUS systematic biopsies	ISUP	Subsequent	All

© 2024 Haj-Mirzaian A et al. *JAMA Network Open.*

				prostatic hyperplasia, having a previous diagnosis of PCa, exhibiting isolated atypical small acinar proliferation, and exhibiting high-grade prostatic intraepithelial neoplasia with atypia.	Moderate risk			when the PI-RADS reported in the pre-biopsy mpMRI was 3 or higher, while 12-core TRUS systematic biopsies were performed when the PI-RADS was lower than 3.			
Gan et al., 2022	Retro.	NA	Suspected prostate cancer - Indications for prebiopsy bpMRI or mpMRI include elevated serum PSA level, family history of prostate cancer, and abnormal DRE. Biopsy naïve patients.	PIRADS ≥3.	Subjects without but suspected to have csPCa – Low risk	1.5 & 3T mp and bp	PSAD	Systematic biopsy	ISUP	Subsequent	PI-RADS ≤2
Zhang et al., 2022	Retro.	Cons.	Suspected prostate cancer - Underwent mpMRI followed by systematic biopsy. Included patients receiving a first biopsy with negative mpMRI.	Previous biopsy, patients with either or both prebiopsy PSA and mpMRI performed elsewhere, and patients who had received any previous surgical treatment for their prostate. PIRADS ≥3.	Subjects without but suspected to have csPCa – Low risk	3T mp	Age, PSA, PV, PSAD	Systematic biopsy	ISUP	Subsequent <1 month	PI-RADS ≤2
Zhou et al., 2022	Retro.	Cons.	Suspected prostate cancer - Biopsy-naïve patients with suspected PCa who subsequently underwent MRI targeted biopsy combined with trans-perineal ultrasound-guided systematic biopsy. At least one of the following criteria were considered eligible for prostate biopsy: elevated PSA (>4.0 ng/ml), free/total PSA ratio < 0.16, or PSAD > 0.15 ng/ml/ml, abnormal DRE,	Presence of a medical history of 5-α-reductase inhibitors, evidence of acute prostatitis, urinary tract infection, and those with a previous history of prostatic surgery for any prostatic condition. Patients who underwent repeat prostate or with incomplete data were also excluded. Furthermore, patients with atypical small acinar proliferation or high-grade intraepithelial	Subjects without but suspected to have csPCa - Moderate risk	3T mp	Age, PSA, PV, PSAD	Targeted biopsy combined with trans-perineal ultrasound-guided systematic biopsy	ISUP	Subsequent	All

© 2024 Haj-Mirzaian A et al. *JAMA Network Open.*

			prostate ultrasound showing focal lesion, and at least one suspicious MR identified lesion with a PI-RADS score of ≥3.	neoplasia due to the small number of cases.						
van Riel et al., 2022	Pros.	NA	Suspected prostate cancer - Biopsy naïve or prior negative men if their prostate MRI was negative (PI-RADS 1-2) and performed within 6 months prior to prostate biopsy.	Men with prior PCa treatment or prior positive prostate biopsies.	Subjects without but suspected to have csPCa – Low risk	1.5 & 3T NA	Multiple risk scores	Systematic prostate biopsy procedures were performed transrectally or transperineally.	ISUP	Subsequent <6 months	PI-RADS ≤2
Morote, Campistol, et al., 2022	Pros.	Cons.	Suspected prostate cancer - PSA >3 ng/ml and/or abnormal DRE underwent prebiopsy mpMRI and had guided and systematic prostate biopsies. PI-RADS 3.	AS and those with symptomatic benign prostatic hyperplasia treated with 5a-reductase inhibitors.	Subjects without but suspected to have csPCa – Low risk	3T mp	Multiple risk scores, PSAD	Two- to three-core trans-rectal ultrasound-guided biopsies of suspected lesions and 12-core systematic biopsies.	ISUP	Subsequent	PI-RADS =3
Wagaskar et al., 2022	Retro.	NA	Suspected prostate cancer - PSA >4ng/ml and 4Kscore of >7%; PSAD >1.5; suspicious DRE; or PI-RADS scores of 3, 4, or 5 on mpMRI, or a combination of any of the above.	Contra-indication for mpMRI; prior hormone therapy or radiation; or missing data on family history of prostate cancer, history of prior negative biopsy or DRE. Prior PCa.	Subjects without but suspected to have csPCa - Moderate risk	3T mp	PNBx, PSAD, DRE, FHx, age	Underwent either systematic or systematic and MRI-TRUS fusion targeted biopsy in the case of a positive MRI (PI-RADS >3), and 2-4 extra cores from each lesion.	ISUP	Subsequent	All
Chau et al., 2022	Pros.	NA	Suspected prostate cancer - Men had mpMRI and were biopsy naïve.	PSA>30ng/mL or any missing data required for the logistic regression analysis.	Subjects without but suspected to have csPCa - Moderate risk	1.5 & 3T NA mp	Age, PSAD	All men with a positive mpMRI had image guided targeted biopsy in addition to systematic sampling. Men with negative mpMRI findings were offered systematic biopsy only.	ISUP	Subsequent	All
Frisbie et al., 2022	Retro.	NA	Suspected prostate cancer - Patients were identified with visible lesions on	Previous PCa diagnosis or treatment.	Subjects without but suspected to	NA mp	PSA, age, race, PSAD	Standard 12-core template biopsy and two targeted biopsies per lesion.	ISUP	Subsequent	All

© 2024 Haj-Mirzaian A et al. *JAMA Network Open.*

			prostate MRI and subsequent prostate biopsy.		have csPCa - Moderate risk						
Girometti et al., 2022	Retro.	NA	Suspected prostate cancer - Underwent mpMRI because of persistently increased serum PSA level and/or suspicious DRE. In all patients, serum PSA level was obtained ≤1 month before mpMRI. All biopsy naïve.	Absence of contrast administration due to contraindications, poor image quality due to artifacts, and ongoing therapy with 5-alfa reductase inhibitor at the time of mpMRI	Subjects without but suspected to have csPCa - Moderate risk	3T mp	PSAD	Targeted for suspected lesions (PI-RADS >=3) and then systemic in all.	ISUP	Subsequent	All
Wei et al., 2022	Retro.	Cons.	Suspected prostate cancer - Underwent prostate MRI and a subsequent prostate biopsy.	Incomplete MRI examination or clinical data (including PSA and the pathological results of the biopsy), MRI was performed on 1.5-T scanner; history of endocrine therapy, radiotherapy or prostate surgery, or prior PCa diagnosis; image quality poor and prevented PI-RADS scoring; PSA > 100 ng/ml.	Subjects without but suspected to have csPCa - Moderate risk	3T mp	PSAD	Underwent a 6- or 12-core biopsy as a systematic biopsy. One or two cognitive fusion-targeted biopsy cores were added through MRI-TRUS fusion-guided biopsy for suspicious or equivocal lesions which were classified with a PI-RADS score of 3 or greater.	ISUP	Subsequent	All
Pan et al., 2021	Retro.	NA	Suspected prostate cancer - PSA > 4 ng/ml and/or suspicious DRE result, and patients who underwent prostate bpMRI scan within 3 months prior to biopsy. Included biopsy naïve or prior negative biopsy.	Treatment prior to biopsy (e.g., prostate surgery, chemotherapy, radiation therapy, or androgen deprivation therapy), bpMRI scan performed after biopsy, and incomplete data.	Subjects without but suspected to have csPCa - Moderate risk	1.5T bp	Age, PSA, F/T PSA, PSAD	Ten biopsy samples were obtained from each patient. If suspicious areas were found in MRI or ultrasound images, additional one to two targeted cores were also performed.	ISUP	Subsequent <3 months	All
Ryoo et al., 2020	Retro.	NA	Suspected prostate cancer - Underwent mpMRI before a prostate biopsy, who had PSA value from	Per inclusion.	Subjects without but suspected to have csPCa –	3T mp	Age, PSA, PV, PSAD	Standard paired sextant pattern and MRI/TRUS fusion target biopsy and cognitive target biopsy on lesions	ISUP	Subsequent	All

© 2024 Haj-Mirzaian A et al. *JAMA Network Open.*

			2.5 to 15 ng/mL and who underwent their second prostate biopsy with an initial benign negative prostatic biopsy or their first prostate biopsy.		Low or moderate risk			with PI- RADS scores 1 to 5 on mpMRI.			
Deniffel et al., 2020	Retro.	NA	Suspected prostate cancer - mpMRI on patients without a prior PCa diagnosis.	Lack of serum PSA records 6 months or more before mpMRI, magnetic field strength 1.5 T or incomplete radiology reports.	Subjects without but suspected to have csPCa - Moderate risk	3T mp	Age, PV, PSA, PSAD	Fusion techniques, and/or systematic TRUS guided biopsy after mpMR.	ISUP	Subsequent <1 year	All
Campistol et al., 2022	Pros.	Cons.	Men with suspected PCa due to PSA levels > 3 ng/mL and/or abnormal DRE scheduled for a 3-tesla mpMRI prior to biopsy	Men with PCa on active surveillance and those with symptomatic benign prostatic hyperplasia treated with 5- α-reductase inhibitors.	Subjects without but suspected to have csPCa - Moderate risk	3T mp	ERSPC	Twelve core transrectal ultrasound (TRUS) systematic-biopsies were performed in all participants, and two to four core TRUS cognitive fusion biopsies were taken in those patients with suspicious lesions (PI-RADSv.2 ≥ 3).	ISUP	Subsequent	All
Hogan et al., 2022	Retro.	Cons.	Biopsy naïve patients with no prior diagnosis of PCa who underwent pre-biopsy mpMRI followed by trans-perineal biopsy of the prostate.	Per inclusion.	Subjects without but suspected to have csPCa - Moderate risk	Majority 3T mp	PSAD	Underwent systematic template biopsy under general anaesthesia. Cognitive target biopsy was performed in addition to the systematic biopsy in patients with a lesion present on pre-biopsy mpMRI.	ISUP	Subsequent	All
Maggi et al., 2021	Pros.	Cons.	All patients were scheduled for first prostate biopsy, and inclusion criteria were: elevated total PSA level (>3 ng/mL confirmed) and/or abnormal DRE.	History of PCa or different neoplasm under treatment, any medical treatment that could alter PSA value, any invasive treatments for BPH or any prior prostatic biopsy.	Subjects without but suspected to have csPCa - Moderate risk	1.5 & 3T mp	Select MDx, PSA, PSAD	12 random systematic cores. In all patients where a mpMRI PI-RADS 3–5 lesion has been described, additional targeted samples (2 to 3 biopsy cores per lesion) have been obtained using an imaging fusion technique.	ISUP	Subsequent	All
Wei et al., 2021	Retro.	Cons.	Underwent biparametric MRI, and pathological results were obtained by systematic transrectal	Per inclusion.	Subjects without but suspected to	3T bp	PSAD	10-core systematic TRUS-guided prostate biopsy. In addition, MRI-TRUS fusion targeted biopsy was used for suspicious PCa lesions on	ISUP	Subsequent	TZ

© 2024 Haj-Mirzaian A et al. *JAMA Network Open.*

			ultrasound-guided prostate biopsy and/or MRI-TRUS fusion targeted biopsy. All patients had either elevated PSA levels or abnormal findings on DRE. Eligible patients who had a dominant lesion in the TZ were included in this study.		have csPCa - Low risk			MRI and 2–3 targeted cores were added for these lesions.			
Wang et al., 2021	Retro.	NA	All patients derived from elevated PSA levels within 4-20 ng/mL. Patients who had the index lesion in TZ which was determined as the lesion with the highest PI-RADS v2.1 score.	Index lesion in PZ, incomplete bp-MRI examination, unsatisfactory MR images affected by artifacts from patient movement or hip replacement and prostate biopsy or therapies before MRI examination.	Subjects without but suspected to have csPCa - Low risk	3T bp	PSAD	10-core systematic transrectal ultrasound guided prostate biopsy. For suspicious PCa lesions on MRI (PI-RADS v2.1 score ≥ 3), an MRI-TRUS fusion-guided targeted biopsy was used, and then, 2-3 targeted cores would be added for these lesions.	ISUP	Subsequent	TZ
Morote et al., 2020	Retro.	NA	Undergoing 5-alfa-reductase inhibitors treatment as well as those with previous PCa. All previous negative biopsy. Prebiopsy MRI and then biopsy.	Per inclusion.	Subjects without but suspected to have csPCa - Moderate risk	3T bp	PSAD, DRE, age	At least 2-core TRUS-MRI cognitive-fusion targeted biopsies of each PI-RADSv2 ≥3 lesion, and/or 12-core TRUS systematic biopsy, both through trans-rectal approach.	ISUP	Subsequent	All
Püllen et al., 2019	Retro.	Cons.	Biopsy-naïve or had undergone a previous negative biopsy. All the men received mpMRI and subsequent TRUS/MRI fusion and systematic biopsy.	Per inclusion.	Subjects without but suspected to have csPCa - Moderate risk	3T mp	Multiple risk score models	Systematic and TRUS/MRI fusion-targeted (median of 4 cores/lesion) trans-perineal biopsy.	ISUP	Subsequent	All
Petersmann et al., 2021	NA	NA	Clinical suspicion of prostate cancer. Patients were either biopsy naïve or had a previous negative biopsy.	Per inclusion	Subjects without but suspected to have csPCa -	3T mp	Multiple risk score models	All patients received an mpMRI followed by a trans-perineal MRI/TRUS fusion biopsy, systematic and/or targeted (suspicious lesions with PI-RADS ≥ 3).	ISUP	Subsequent	All

© 2024 Haj-Mirzaian A et al. *JAMA Network Open.*

					Moderate risk						

* All the included studies followed the PI-RADS MRI acquisition protocol.

© 2024 Haj-Mirzaian A et al. *JAMA Network Open.*

eTable 4. Baseline Characteristics of the Included Studies

	Number	csPCa (rate)	Non-sig PCa (rate)	Age (years)	BMI (kg/m2)	Total PSA (ng/mL)	Free/Total PSA (ratio)	Free PSA (ng/mL)	Prostate volume (mL^2)	PSAD (ng/mL^2)
Bittencourt et al., 2022	499	0.53	0.23	65.20	NA	5.60	NA	NA	43.00	0.13
Wang *et al.*, 2021	333	0.10	0.10	69.30	NA	9.48	0.15	1.42	62.95	0.16
Lendínez-Cano et al., 2021	163	0.32	0.12	62.00	NA	5.21	NA	13.00	43.00	NA
Sonmez et al., 2021	508	0.39	NA	62.49	30.79	7.28	NA	NA	NA	0.13
Keck et al., 2021	289	0.42	0.09	66.00	NA	8.19	NA	NA	NA	NA
Deniffel et al., 2021	385	0.41	0.16	65.74	NA	7.86	NA	NA	58.40	0.13
Tosun and Uslu, 2021	150	0.19	0.29	61.85	NA	9.52	0.19	NA	44.34	0.12
Liang et al., 2021	225	0.16	0.11	66.00	24.15	9.38	NA	NA	43.35	0.21
Fan et al., 2021	164	0.41	0.16	67.00	NA	9.05	0.15	NA	53.20	0.16
Noh et al., 2020	300	0.34	0.19	66.00	24.70	11.30	NA	NA	NA	0.33
Apfelbeck et al., 2020	848	0.80	NA	69.00	NA	8.22	NA	NA	47.00	0.18
Morote *et al.*, 2020	377	0.34	0.07	68.00	NA	8.10	NA	NA	NA	0.13
Falagario et al., 2020	2512	0.37	0.18	64.00	NA	6.20	NA	NA	47.00	0.13
Sokhi et al., 2020	262	0.390	0.11	NA	NA	14.7 or 8.7	NA	NA	NA	NA
Anastay et al., 2020	191	0.05	0.21	65.00	NA	9.30	NA	NA	60.00	0.15
Sonmez et al., 2020	288	0.22	0.03	62.18	27.00	7.20	NA	NA	61.31	NA
Kim et al., 2020	755	0.33	0.09	65.30	24.70	11.17	NA	NA	43.40	0.29
Busetto et al., 2020	52	0.135	0.33	64.00	NA	6.80	NA	NA	47.60	0.16
Stevens et al., 2020	526	0.32	0.20	63.88	NA	8.82	NA	NA	59.84	0.17
Wei et al., 2020	364	0.14	0.13	68.34	NA	7.22	0.16	NA	49.92	0.15
Al Hussein Al Awamlh et al., 2020	247	0.22	NA	63.55	NA	6.40	NA	NA	51.56	0.15
Han et al., 2020	123	0.30	NA	66.30	NA	7.23	NA	NA	NA	0.21
He et al., 2019	385	0.405	0.10	67.00	24.22	9.49	NA	NA	37.52	NA
Borque-Fernando et al., 2019	346	0.33	0.07	67.70	NA	6.10	NA	1.17	50.50	0.13

© 2024 Haj-Mirzaian A et al. *JAMA Network Open.*

Hsieh et al., 2019	102	0.235	0.15	65.50	NA	7.78	0.17	NA	NA	NA
Lu, Zhang, Chen, et al., 2019	357	0.42	0.10	68.00	NA	11.06	NA	NA	49.18	NA
Boesen, Nørgaard, et al., 2019	808	0.35	0.22	65.00	NA	6.90	NA	NA	54.00	0.12
Lu, Zhang, Yao, et al., 2019	357	0.46	0.06	68.00	NA	11.06	0.13	NA	NA	0.24
Boesen, Thomsen, et al., 2019	876	0.40	0.20	65.00	NA	7.30	NA	NA	53.00	0.13
Bhat et al., 2018	372	0.41	NA	64.70	NA	9.40	NA	NA	58.40	0.20
Kim et al., 2018	138	0.10	0.07	63.90	24.90	9.10	NA	NA	45.80	NA
Cuocolo et al., 2018	114	0.33	0.20	65.75	NA	7.76	NA	NA	61.37	0.14
Hansen et al., 2017	807	0.49	0.20	65.00	NA	6.50	NA	NA	42.00	0.15
Radtke et al., 2017	1159	0.42	0.21	65.00	NA	7.30	NA	NA	45.00	0.16
Distler et al., 2017	1040	0.43	0.20	65.00	NA	7.20	NA	NA	45.00	0.16
van Leeuwen et al., 2017	591	0.40	0.22	63.01	NA	5.57	NA	NA	42.01	NA
Washino et al., 2016	288	0.49	0.06	69.00	NA	7.50	NA	NA	28.70	0.26
Mehralivand et al., 2018	651	0.44	0.23	64.39	NA	6.40	NA	NA	50.52	0.12
Alberts et al., 2018	1353	0.35	0.16	66.00	NA	8.70	NA	NA	49.70	0.17
Sakaguchi et al., 2021	773	0.44	NA	66.77	NA	7.74	NA	NA	35.30	NA
Liu et al., 2021	1062	0.12	NA	68.87	24.22	12.51	0.14	NA	NA	0.21
Zhang et al., 2019	218	0.15	NA	69.10	NA	10.75	NA	NA	51.93	0.19
Görtz et al., 2019	101	0.15	0.16	64.00	NA	6.70	NA	NA	63.00	0.10
Saba et al., 2019	468	0.41	0.12	64.50	NA	6.60	NA	NA	48.90	NA
Radtke et al., 2019	293	0.39	NA	65.00	NA	7.20	0.16	NA	42.00	NA
Záleský et al., 2019	397	0.35	0.13	62.70	NA	8.52	NA	NA	61.13	0.16
Punnen et al., 2018	149	0.33	0.16	66.00	NA	6.30	NA	NA	NA	NA
Truong et al., 2017	285	0.39	0.15	66.00	28.60	8.80	NA	NA	56.10	NA
Tan et al., 2017	115	0.35	0.09	69.00	NA	8.70	NA	NA	56.00	0.16
Hansen et al., 2016	514	0.31	NA	65.00	NA	7.60	NA	NA	59.00	0.13
Thompson et al., 2015	344	0.42	0.21	62.90	NA	5.20	NA	NA	NA	NA
Zhu et al., 2022	115	0.06	0.06	66.00	NA	8.03	NA	NA	56.00	0.13

© 2024 Haj-Mirzaian A et al. *JAMA Network Open.*

Campistol *et al.*, 2022	567	0.41	0.12	69.00	NA	7.00	0.15	1.10	55.00	0.13
Morote, Borque-Fernando, et al., 2022	2432	0.38	0.12	68.00	NA	6.50	NA	NA	55.00	0.12
Gan et al., 2022	155	0.15	0.16	NA	NA	NA	NA	NA	NA	NA
Zhang et al., 2022	240	0.09	0.07	63.90	NA	11.57	0.14	1.31	49.66	0.21
Zhou et al., 2022	688	0.465	0.15	66.48	NA	9.08	0.15	NA	40.73	0.24
van Riel et al., 2022	232	0.17	0.19	64.00	NA	6.50	NA	NA	55.00	0.12
Morote, Campistol, et al., 2022	169	0.15	0.17	66.00	NA	6.00	0.16	1.10	66.00	0.11
Wagaskar et al., 2022	2254	0.39	0.22	63.41	NA	5.73	NA	NA	NA	0.12
Chau et al., 2022	506	0.45	0.18	66.00	NA	6.60	NA	NA	43.00	0.14
Hogan *et al.*, 2022	140	0.29	0.29	61.30	NA	6.00	NA	NA	38.00	0.15
Frisbie et al., 2022	327	0.26	0.16	65.00	NA	8.00	NA	NA	55.00	NA
Girometti et al., 2022	123	0.44	0.11	67.00	NA	6.16	NA	NA	NA	0.12
Wei et al., 2022	508	0.25	0.08	66.69	NA	12.31	0.14	NA	48.66	0.30
Pan et al., 2021	530	0.33	0.09	67.37	NA	11.29	0.13	1.32	41.75	0.29
Wei *et al.*, 2021	511	0.145	0.09	69.06	NA	11.12	0.15	NA	61.15	0.21
Ryoo et al., 2020	1098	0.29	0.13	65.00	NA	5.10	NA	NA	37.30	0.14
Maggi *et al.*, 2021	310	0.20	0.14	64.00	NA	7.60	NA	NA	59.00	0.14
Deniffel et al., 2020	865	0.28	0.10	62.97	NA	7.91	NA	NA	61.88	0.12
Petersmann *et al.*, 2021	265	0.46	0.17	68.00	NA	9.80	NA	NA	53.00	NA
Püllen *et al.*, 2019	307	0.40	0.20	67.00	NA	8.80	NA	NA	55.00	0.16

© 2024 Haj-Mirzaian A et al. *JAMA Network Open.*

eTable 4. Baseline characteristics of the included studies (continued).

	Positive Family Hx (rate)	Positive DRE (rate)	PZ (rate)	TZ (rate)	Start year	End year	1.5T MRI (rate)	3 MRI (rate)	Reliably for PIRADS (Kappa/ICC)
Bittencourt et al., 2022	NA	NA	0.65	0.35	2016	2019	Both	Both	NA
Wang *et al.*, 2021	NA	NA	0.00	1.00	2016	2020	0.00	1.00	NA
Lendínez-Cano et al., 2021	0.10	NA	NA	NA	2019	2020	1.00	0.00	NA
Sonmez et al., 2021	NA	NA	NA	NA	2016	2020	1.00	0.00	NA
Keck et al., 2021	NA	NA	NA	NA	2015	2016	0.00	1.00	NA
Deniffel et al., 2021	NA	0.13	NA	NA	2015	2019	0.00	1.00	NA
Tosun and Uslu, 2021	NA	NA	NA	NA	2015	2018	0.00	1.00	0.97
Liang et al., 2021	NA	0.16	NA	NA	2018	2019	0.00	1.00	NA
Fan et al., 2021	NA	0.33	NA	NA	2017	2019	0.00	1.00	NA
Noh et al., 2020	NA	NA	NA	NA	2019	2020	0.00	1.00	NA
Apfelbeck et al., 2020	NA	NA	0.48	0.19	2015	2020	NA	NA	NA
Morote *et al.*, 2020	NA	0.28	NA	NA	2014	2017	0.00	1.00	NA
Falagario et al., 2020	NA	0.25	NA	NA	2013	2019	1.00	1.00	NA
Sokhi et al., 2020	NA	0.51	NA	NA	2017	2018	1.00	0.00	NA
Anastay et al., 2020	NA	0.14	NA	NA	2014	2019	both	both	NA
Sonmez et al., 2020	0.35	NA	NA	NA	2017	2019	0.00	1.00	NA
Kim et al., 2020	NA	NA	0.39	0.61	2013	2019	0.00	1.00	NA
Busetto et al., 2020	0.19	0.19	NA	NA	2018	2019	0.00	1.00	NA
Stevens et al., 2020	NA	NA	NA	NA	2014	2018	0.00	1.00	NA
Wei et al., 2020	NA	NA	NA	NA	2015	2019	0.00	1.00	0.751
Al Hussein Al Awamlh et al., 2020	NA	NA	0.64	0.36	2015	2018	0.00	1.00	NA
Han et al., 2020	NA	NA	NA	NA	2010	2017	0.00	1.00	NA
He et al., 2019	NA	0.12	NA	NA	2015	2018	0.00	1.00	NA
Borque-Fernando et al., 2019	NA	0.32	NA	NA	2015	2016	0.00	1.00	NA

© 2024 Haj-Mirzaian A et al. *JAMA Network Open.*

Hsieh et al., 2019	NA	0.38	NA	NA	2016	2018	0.00	1.00	NA
Lu, Zhang, Chen, et al., 2019	NA	NA	NA	NA	2014	2017	0.00	1.00	NA
Boesen, Nørgaard, et al., 2019	NA	NA	NA	NA	2015	2017	0.00	1.00	NA
Lu, Zhang, Yao, et al., 2019	NA	NA	NA	NA	2014	2017	0.00	1.00	NA
Boesen, Thomsen, et al., 2019	NA	0.30	NA	NA	2015	2017	0.00	1.00	NA
Bhat et al., 2018	0.27	0.15	NA	NA	2014	2016	0.00	1.00	NA
Kim et al., 2018	NA	NA	0.53	0.47	2016	2018	0.00	1.00	NA
Cuocolo et al., 2018	NA	NA	NA	NA	2015	2017	0.00	1.00	NA
Hansen et al., 2017	NA	0.23	NA	NA	2012	2016	both	both	NA
Radtke et al., 2017	NA	0.23	NA	NA	2012	2015	0.00	1.00	NA
Distler et al., 2017	NA	NA	NA	NA	2012	2015	0.00	1.00	NA
van Leeuwen et al., 2017	0.27	0.36	NA	NA	2012	2014	both	both	NA
Washino et al., 2016	NA	0.16	NA	NA	2010	2014	0.41	0.59	NA
Mehralivand et al., 2018	0.24	0.10	NA	NA	2015	2016	0.00	1.00	NA
Alberts et al., 2018	NA	0.22	NA	NA	2012	2017	0.00	1.00	NA
Sakaguchi et al., 2021	NA	NA	NA	NA	2011	2016	both	both	NA
Liu et al., 2021	0.01	NA	NA	NA	2014	2020	0.00	1.00	0.822
Zhang et al., 2019	NA	NA	0.39	0.61	2012	2018	0.00	1.00	NA
Görtz et al., 2019	NA	0.08	NA	NA	2017	2018	0.00	1.00	NA
Saba et al., 2019	0.11	0.16	NA	NA	2014	2018	NA	NA	NA
Radtke et al., 2019	NA	0.35	NA	NA	2010	2017	both	both	NA
Záleský et al., 2019	NA	NA	NA	NA	NA	NA	1.00	0.00	NA
Punnen et al., 2018	NA	0.68	NA	NA	2014	2017	0.00	1.00	NA
Truong et al., 2017	NA	NA	NA	NA	2014	2016	0.00	1.00	NA
Tan et al., 2017	NA	NA	NA	NA	2016	2017	0.00	1.00	NA
Hansen et al., 2016	NA	NA	NA	NA	2013	2015	both	both	NA
Thompson et al., 2015	0.27	0.44	NA	NA	2012	2014	0.49	0.51	NA
Zhu et al., 2022	NA	NA	NA	NA	2012	2022	0.00	1.00	NA

© 2024 Haj-Mirzaian A et al. *JAMA Network Open.*

Campistol *et al.*, 2022	0.09	0.19	NA	NA	2018	2020	0.00	1.00	NA
Morote, Borque-Fernando, et al., 2022	0.07	0.25	NA	NA	2006	2019	0.00	1.00	NA
Gan et al., 2022	NA	NA	NA	NA	2017	2020	0.00	1.00	NA
Zhang et al., 2022	NA	NA	NA	NA	2017	2021	0.00	1.00	NA
Zhou et al., 2022	NA	NA	NA	NA	2016	2019	0.00	1.00	NA
van Riel et al., 2022	0.13	0.17	NA	NA	2015	2021	both	both	NA
Morote, Campistol, et al., 2022	0.07	0.07	NA	NA	2018	2020	0.00	1.00	NA
Wagaskar et al., 2022	0.24	0.32	NA	NA	2014	2020	0.00	1.00	NA
Chau et al., 2022	NA	NA	NA	NA	2018	2022	1.00	1.00	NA
Hogan *et al.*, 2022	0.14	0.33	NA	NA	2017	2018	NA	NA	NA
Frisbie et al., 2022	NA	NA	NA	NA	2015	2019	NA	NA	NA
Girometti et al., 2022	NA	NA	NA	NA	2019	2020	0.00	1.00	NA
Wei et al., 2022	NA	NA	NA	NA	2013	2019	1.00	1.00	0.854
Pan et al., 2021	NA	NA	NA	NA	2018	2020	1.00	0.00	NA
Wei *et al.*, 2021	NA	NA	0.00	1.00	2015	2020	0.00	1.00	0.648
Ryoo et al., 2020	NA	NA	NA	NA	2016	2018	0.00	1.00	NA
Maggi *et al.*, 2021	0.19	0.25	NA	NA	2018	2019	both	both	NA
Deniffel et al., 2020	NA	NA	NA	NA	2009	2017	0.00	1.00	NA
Petersmann *et al.*, 2021	NA	0.24	NA	NA	2016	2019	0.00	1.00	NA
Püllen *et al.*, 2019	NA	0.19	NA	NA	2015	2018	0.00	1.00	NA

© 2024 Haj-Mirzaian A et al. *JAMA Network Open.*

eTable 4. Baseline characteristics of the included studies (continued).

	PI-RADS									
	**1 or 2**	**3**	**4 or 5**	**4**	**5**	**PNB (ratio)**	**Biopsy naïve (ratio)**	**Prior biopsy (ratio)**	**Gleason 6 baseline (ratio)**	**Race**
Bittencourt et al., 2022	0.12	0.15	0.74	0.55	0.18	0.00	1.00	0.00	0.00	NA
Wang *et al.*, 2021	0.64	0.21	0.15	0.08	0.07	0.00	1.00	0.00	0.00	NA
Lendínez-Cano et al., 2021	0.36	0.17	0.48	0.35	0.13	0.00	1.00	0.00	0.00	0.99 White
Sonmez et al., 2021	0.00	0.33	0.68	0.49	0.18	0.28	0.72	0.28	0.00	NA
Keck et al., 2021	0.09	0.23	68.80	0.46	0.23	NA	0.27	0.73	NA	NA
Deniffel et al., 2021	0.00	0.24	0.76	0.51	0.24	0.83	NA	NA	0.00	0.01 AA
Tosun and Uslu, 2021	0.34	0.20	0.46	0.30	0.16	NA	NA	NA	NA	NA
Liang et al., 2021	1.00	0.00	0.00	0.00	0.00	0.08	0.92	0.08	0.00	NA
Fan et al., 2021	NA	NA	NA	NA	NA	0.28	0.72	0.28	0.00	NA
Noh et al., 2020	0.15	0.34	0.51	0.31	0.21	NA	NA	NA	NA	NA
Apfelbeck et al., 2020	0.00	0.00	1.00	0.58	0.42	NA	NA	NA	NA	NA
Morote *et al.*, 2020	0.30	0.38	0.32	0.26	0.05	1.00	0.00	0.00	0.00	NA
Falagario et al., 2020	0.19	0.27	0.54	0.32	0.21	0.28	0.72	0.28	0.00	NA
Sokhi et al., 2020	0.33	0.08	0.59	0.21	0.38	0.10	0.91	0.10	0.00	NA
Anastay et al., 2020	1.00	0.00	0.00	0.00	0.00	0.19	0.66	0.34	0.15	NA
Sonmez et al., 2020	0.00	1.00	0.00	0.00	0.00	0.34	0.66	0.34	0.00	NA
Kim et al., 2020	0.00	0.46	0.54	0.34	0.27	0.37	0.58	0.42	0.06	NA
Busetto et al., 2020	0.56	0.21	0.23	NA	NA	0.00	1.00	0.00	0.00	NA
Stevens et al., 2020	0.23	0.23	0.53	0.34	0.29	NA	NA	NA	NA	NA
Wei et al., 2020	0.55	0.19	0.26	0.20	0.07	0.00	1.00	0.00	0.00	NA
Al Hussein Al Awamlh et al., 2020	0.00	1.00	0.00	0.00	0.00	0.00	1.00	0.00	0.00	NA
Han et al., 2020	0.53	0.15	0.33	0.17	0.15	NA	NA	NA	NA	NA

He et al., 2019	0.37	0.20	0.43	0.25	0.18	0.18	0.82	0.18	0.00	NA
Borque-Fernando et al., 2019	0.23	0.27	0.50	NA	NA	NA	0.47	0.53	NA	NA
Hsieh et al., 2019	0.34	0.08	0.58	0.37	0.21	0.33	0.67	0.33	0.00	NA
Lu, Zhang, Chen, et al., 2019	0.43	0.10	0.47	NA	NA	0.00	1.00	0.00	0.00	NA
Boesen, Nørgaard, et al., 2019	0.37	0.15	0.48	NA	NA	0.00	1.00	0.00	0.00	NA
Lu, Zhang, Yao, et al., 2019	0.43	0.10	0.47	NA	NA	NA	NA	NA	NA	NA
Boesen, Thomsen, et al., 2019	0.34	0.15	0.52	0.20	0.32	0.00	1.00	0.00	0.00	NA
Bhat et al., 2018	0.19	0.21	0.61	0.32	0.29	0.48	0.52	0.48	0.00	NA
Kim et al., 2018	0.00	1.00	0.00	0.00	0.00	0.42	0.58	0.42	0.00	NA
Cuocolo et al., 2018	0.48	0.08	0.44	0.21	0.23	0.00	1.00	0.00	0.00	NA
Hansen et al., 2017	0.29	0.19	0.52	NA	NA	0.00	1.00	0.00	0.00	NA
Radtke et al., 2017	0.15	0.33	0.52	0.32	0.20	0.42	0.58	0.42	0.00	NA
Distler et al., 2017	0.33	0.24	0.43	0.25	0.18	0.57	0.43	0.57	0.00	NA
van Leeuwen et al., 2017	0.34	NA	NA	NA	NA	0.13	NA	NA	NA	NA
Washino et al., 2016	0.44	0.15	0.41	NA	NA	0.00	1.00	0.00	0.00	NA
Mehralivand et al., 2018	0.11	0.17	0.72	0.42	0.29	0.49	0.51	0.49	NA	0.14 AA
Alberts et al., 2018	0.18	0.18	0.64	0.40	0.24	0.59	0.41	0.59	0.00	NA
Sakaguchi et al., 2021	0.22	0.27	0.51	0.23	0.28	0.00	1.00	0.00	0.00	NA
Liu et al., 2021	1.00	0.00	0.00	0.00	0.00	0.12	0.88	0.12	0.00	NA
Zhang et al., 2019	0.00	1.00	0.00	0.00	0.00	0.00	1.00	0.00	0.00	NA
Görtz et al., 2019	0.00	1.00	0.00	0.00	0.00	0.00	1.00	0.00	0.00	NA
Saba et al., 2019	0.33	0.20	0.47	0.35	0.12	0.31	0.69	0.31	0.00	NA
Radtke et al., 2019	0.00	0.40	0.60	0.32	0.28	0.00	1.00	0.00	0.00	NA
Záleský et al., 2019	0.20	0.35	0.45	0.36	0.09	NA	0.56	0.44	NA	NA
Punnen et al., 2018	0.16	0.38	0.46	0.38	0.08	NA	0.52	0.48	NA	NA
Truong et al., 2017	0.00	0.31	0.69	0.36	0.33	1.00	0.00	1.00	0.00	0.78 White, 0.105 AA,

© 2024 Haj-Mirzaian A et al. *JAMA Network Open.*

										0.12 Other
Tan et al., 2017	0.00	0.61	0.39	0.30	0.10	0.64	0.24	0.76	0.11	NA
Hansen et al., 2016	0.34	0.24	0.43	NA	NA	0.68	0.00	1.00	0.32	NA
Thompson et al., 2015	0.23	0.40	0.37	0.26	0.11	0.00	1.00	0.00	0.00	NA
Zhu et al., 2022	1.00	0.00	0.00	0.00	0.00	0.00	1.00	0.00	0.00	NA
Campistol *et al.*, 2022	0.18	0.30	0.53	0.34	0.19	0.24	0.77	0.24	0.00	NA
Morote, Borque-Fernando, et al., 2022	0.23	0.27	0.51	0.35	0.16	0.22	0.78	0.22	0.00	NA
Gan et al., 2022	1.00	0.00	0.00	0.00	0.00	NA	1.00	0.00	0.00	NA
Zhang et al., 2022	1.00	0.00	0.00	0.00	0.00	0.00	1.00	0.00	0.00	NA
Zhou et al., 2022	0.00	0.09	0.91	0.58	0.33	0.00	1.00	0.00	0.00	NA
van Riel et al., 2022	1.00	0.00	0.00	0.00	0.00	0.19	0.81	0.19	0.00	AA/Carrab in 0.21
Morote, Campistol, et al., 2022	0.00	1.00	0.00	0.00	0.00	0.28	0.72	0.28	0.00	NA
Wagaskar et al., 2022	0.29	0.18	0.51	0.36	0.16	0.24	NA	NA	0.00	NA
Chau et al., 2022	0.24	0.21	0.55	0.23	0.32	0.00	1.00	0.00	0.00	NA
Hogan *et al.*, 2022	0.30	0.24	0.46	0.25	0.21	0.00	1.00	0.00	0.00	NA
Frisbie et al., 2022			0.79	0.55	0.24	0.19	NA	NA	0.00	0.29 AA
Girometti et al., 2022	0.24	0.09	0.67	0.38	0.28	0.00	1.00	0.00	0.00	NA
Wei et al., 2022	0.49	0.17	0.33	NA	NA	NA	NA	NA	NA	NA
Pan et al., 2021	0.29	0.28	0.42	0.29	0.13	NA	NA	NA	0.00	NA
Wei *et al.*, 2021	0.56	0.25	0.19	0.06	0.13	NA	NA	NA	NA	NA
Ryoo et al., 2020	0.11	0.19	0.70	0.53	0.17	0.45	0.55	0.45	0.00	NA
Maggi *et al.*, 2021	0.57	0.17	0.25	NA	NA	0.00	1.00	0.00	0.00	NA
Deniffel et al., 2020	0.51	0.30	0.19	0.12	0.07	0.90	0.10	0.90	0.00	NA
Petersmann *et al.*, 2021	0.00	0.20	0.80	0.54	0.26	0.29	0.71	0.29	0.00	NA
Püllen *et al.*, 2019	0.00	0.37	0.63	0.44	0.18	0.30	0.74	0.30	0.00	NA

© 2024 Haj-Mirzaian A et al. *JAMA Network Open.*

eTable 5. Baseline Characteristics of the Included Studies: csPCa vs Non-csPCa (ie, Nonsignificant PCa and Benign)

	Category	Number	csPCa (rate)	Non-sig PCa (rate)	Age (years)	BMI (kg/m2)	Total PSA (ng/mL)	Free/Total PSA (ratio)	Free PSA (ng/mL)	Prostate volume (mL^2)
Wang *et al.*, 2021	csPCa	33	1	0.00	72.00	NA	11.42	0.12	1.50	34.16
Wang *et al.*, 2021	non-csPCa	300	0	1.00	69.00	NA	9.27	0.15	1.41	66.12
Liang et al., 2021	csPCa	37	1	0.00	71.00	23.94	12.60	NA	NA	30.05
Liang et al., 2021	non-csPCa	188	0	0.13	65.70	24.19	9.02	NA	NA	47.42
Fan et al., 2021	csPCa	68	1	0.00	69.00	NA	9.05	0.12	NA	46.80
Fan et al., 2021	non-csPCa	96	0	0.27	66.00	NA	9.02	0.17	NA	56.80
Apfelbeck et al., 2020	csPCa	676	1	NA	70.00	NA	8.32	NA	NA	44.00
Apfelbeck et al., 2020	non-csPCa	172	0	NA	65.00	NA	8.07	NA	NA	65.50
Anastay et al., 2020	csPCa	10	1	0.00	66.00	NA	11.10	NA	NA	62.00
Anastay et al., 2020	non-csPCa	181	0	0.23	65.00	NA	9.10	NA	NA	60.00
Kim et al., 2020	csPCa	250	1	0.00	68.10	24.90	16.68	NA	NA	37.10
Kim et al., 2020	non-csPCa	505	0	0.13	63.85	24.66	8.45	NA	NA	46.46
Busetto et al., 2020	csPCa	7	1	0.00	62.00	NA	12.80	NA	NA	NA
Busetto et al., 2020	non-csPCa	45	0	0.22	65.07	NA	5.87	NA	NA	NA
Wei et al., 2020	csPCa	51	1	0.00	70.45	NA	7.16	0.13	NA	32.36
Wei et al., 2020	non-csPCa	313	0	0.15	68.00	NA	7.23	0.16	NA	52.78
Al Hussein Al Awamlh et al., 2020	csPCa	55	1	NA	65.10	NA	6.94	NA	NA	39.20
Al Hussein Al Awamlh et al., 2020	non-csPCa	192	0	NA	63.10	NA	6.25	NA	NA	55.10
Han et al., 2020	csPCa	37	1	NA	71.20	NA	7.39	NA	NA	NA
Han et al., 2020	non-csPCa	86	0	NA	64.20	NA	7.16	NA	NA	NA
Borque-Fernando et al., 2019	csPCa	113	1	0.00	72.40	NA	8.80	NA	1.07	40.00
Borque-Fernando et al., 2019	non-csPCa	233	0	0.10	66.20	NA	5.70	NA	1.19	56.00
Hsieh et al., 2019	csPCa	24	1	0.00	67.00	NA	9.05	0.13	NA	NA
Hsieh et al., 2019	non-csPCa	78	0	0.19	64.50	NA	7.55	0.19	NA	NA

© 2024 Haj-Mirzaian A et al. *JAMA Network Open.*

Boesen, Nørgaard, et al., 2019	csPCa	283	1	1.00	68.00	NA	8.40	NA	NA	43.00
Boesen, Nørgaard, et al., 2019	non-csPCa	525	0	0.34	64.00	NA	6.30	NA	NA	62.28
Boesen, Thomsen, et al., 2019	csPCa	350	1	0.00	67.00	NA	9.10	NA	NA	43.00
Boesen, Thomsen, et al., 2019	non-csPCa	526	0	0.33	64.00	NA	6.37	NA	NA	61.40
Sakaguchi et al., 2021	csPCa	343	1	0.00	69.00	NA	9.01	NA	NA	29.60
Sakaguchi et al., 2021	non-csPCa	430	0	1.00	65.00	NA	6.72	NA	NA	39.85
Liu et al., 2021	csPCa	127	1	NA	67.50	24.22	16.72	0.11	NA	NA
Liu et al., 2021	non-csPCa	935	0	NA	69.06	24.22	11.94	0.15	NA	NA
Zhang et al., 2022	csPCa	22	1	0.00	69.40	NA	16.16	0.11	1.61	44.88
Zhang et al., 2022	non-csPCa	218	0	0.08	63.30	NA	10.68	0.14	1.31	51.58
Zhou et al., 2022	csPCa	320	1	0.00	69.00	NA	10.71	0.14	NA	35.00
Zhou et al., 2022	non-csPCa	368	0	0.29	64.29	NA	7.67	0.17	NA	45.72
van Riel et al., 2022	csPCa	40	1	0.00	65.00	NA	7.40	NA	NA	44.90
van Riel et al., 2022	non-csPCa	192	0	0.23	64.23	NA	6.25	NA	NA	57.71
Wagaskar et al., 2022	csPCa	874	1	0.00	64.01	NA	6.48	NA	NA	NA
Wagaskar et al., 2022	non-csPCa	1380	0	0.37	63.02	NA	5.26	NA	NA	NA
Wei et al., 2022	csPCa	125	1	0.00	68.65	NA	21.78	0.11	NA	31.02
Wei et al., 2022	non-csPCa	383	0	0.11	66.05	NA	9.22	0.15	NA	54.42
Frisbie et al., 2022	csPCa	175	1	0.00	69.98	NA	14.69	0.11	1.38	33.59
Frisbie et al., 2022	non-csPCa	355	0	0.14	66.08	NA	9.61	0.14	1.29	45.78
Wei *et al.*, 2021	csPCa	74	1	0.00	71.00	NA	19.55	0.12	NA	41.14
Wei *et al.*, 2021	non-csPCa	437	0	0.11	68.73	NA	9.69	0.15	NA	64.54
Maggi *et al.*, 2021	csPCa	62	1	0.00	63.00	NA	9.50	NA	NA	NA
Maggi *et al.*, 2021	non-csPCa	248	0	0.17	64.25	NA	7.13	NA	NA	NA

© 2024 Haj-Mirzaian A et al. *JAMA Network Open.*

eTable 5. Baseline characteristics of the included studies: csPCa versus non-csPCa (continued).

	Category	PSAD (ng/mL^2)	Positive Family Hx (rate)	Positive DRE (rate)	PZ (rate)	TZ (rate)	Start year	End year	1.5T MRI (rate)	3 MRI (rate)
Wang *et al.*, 2021	csPCa	0.31	NA	NA	0.00	1.00	2016	2020	0.00	1.00
Wang *et al.*, 2021	non-csPCa	0.14	NA	NA	0.00	1.00	2016	2020	0.00	1.00
Liang et al., 2021	csPCa	0.42	NA	0.43	NA	NA	2018	2019	0.00	1.00
Liang et al., 2021	non-csPCa	0.19	NA	0.87	NA	NA	2018	2019	0.00	1.00
Fan et al., 2021	csPCa	0.20	NA	0.53	NA	NA	2017	2019	0.00	1.00
Fan et al., 2021	non-csPCa	0.14	NA	0.14	NA	NA	2017	2019	0.00	1.00
Apfelbeck et al., 2020	csPCa	0.19	NA	NA	0.50	0.15	2015	2020	NA	NA
Apfelbeck et al., 2020	non-csPCa	0.13	NA	NA	0.41	0.38	2015	2020	NA	NA
Anastay et al., 2020	csPCa	0.22	NA	0.40	NA	NA	2014	2019	both	both
Anastay et al., 2020	non-csPCa	0.14	NA	0.13	NA	NA	2014	2019	both	both
Kim et al., 2020	csPCa	0.48	NA	NA	0.54	0.46	2013	2019	0.00	1.00
Kim et al., 2020	non-csPCa	0.20	NA	NA	0.32	0.68	2013	2019	0.00	1.00
Busetto et al., 2020	csPCa	0.33	NA	NA	NA	NA	2018	2019	0.00	1.00
Busetto et al., 2020	non-csPCa	0.14	NA	NA	NA	NA	2018	2019	0.00	1.00
Wei et al., 2020	csPCa	0.20	NA	NA	NA	NA	2015	2019	0.00	1.00
Wei et al., 2020	non-csPCa	0.14	NA	NA	NA	NA	2015	2019	0.00	1.00
Al Hussein Al Awamlh et al., 2020	csPCa	0.21	NA	NA	0.66	0.35	2015	2018	0.00	1.00
Al Hussein Al Awamlh et al., 2020	non-csPCa	0.13	NA	NA	0.64	0.37	2015	2018	0.00	1.00
Han et al., 2020	csPCa	0.27	NA	NA	NA	NA	2010	2017	0.00	1.00
Han et al., 2020	non-csPCa	0.18	NA	NA	NA	NA	2010	2017	0.00	1.00
Borque-Fernando et al., 2019	csPCa	0.19	NA	0.43	NA	NA	2015	2016	0.00	1.00
Borque-Fernando et al., 2019	non-csPCa	0.11	NA	0.26	NA	NA	2015	2016	0.00	1.00
Hsieh et al., 2019	csPCa	NA	NA	0.13	NA	NA	2016	2018	0.00	1.00

© 2024 Haj-Mirzaian A et al. *JAMA Network Open.*

Hsieh et al., 2019	non-csPCa	NA	NA	0.25	NA	NA	2016	2018	0.00	1.00
Boesen, Nørgaard, et al., 2019	csPCa	0.18	NA	NA	NA	NA	2015	2017	0.00	1.00
Boesen, Nørgaard, et al., 2019	non-csPCa	0.11	NA	NA	NA	NA	2015	2017	0.00	1.00
Boesen, Thomsen, et al., 2019	csPCa	0.20	NA	0.55	NA	NA	2015	2017	0.00	1.00
Boesen, Thomsen, et al., 2019	non-csPCa	0.11	NA	0.13	NA	NA	2015	2017	0.00	1.00
Sakaguchi et al., 2021	csPCa	NA	NA	NA	NA	NA	2011	2016	both	both
Sakaguchi et al., 2021	non-csPCa	NA	NA	NA	NA	NA	2011	2016	both	both
Liu et al., 2021	csPCa	0.44	0.02	NA	NA	NA	2014	2020	0.00	1.00
Liu et al., 2021	non-csPCa	0.18	0.01	NA	NA	NA	2014	2020	0.00	1.00
Zhang et al., 2022	csPCa	0.39	NA	NA	NA	NA	2017	2021	0.00	1.00
Zhang et al., 2022	non-csPCa	0.20	NA	NA	NA	NA	2017	2021	0.00	1.00
Zhou et al., 2022	csPCa	0.33	NA	NA	NA	NA	2016	2019	0.00	1.00
Zhou et al., 2022	non-csPCa	0.16	NA	NA	NA	NA	2016	2019	0.00	1.00
van Riel et al., 2022	csPCa	0.16	0.33	0.23	NA	NA	2015	2021	both	both
van Riel et al., 2022	non-csPCa	0.12	0.08	0.16	NA	NA	2015	2021	both	both
Wagaskar et al., 2022	csPCa	0.16	0.30	0.44	NA	NA	2014	2020	0.00	1.00
Wagaskar et al., 2022	non-csPCa	0.09	0.20	0.24	NA	NA	2014	2020	0.00	1.00
Wei et al., 2022	csPCa	0.70	NA	NA	NA	NA	2013	2019	1.00	1.00
Wei et al., 2022	non-csPCa	0.16	NA	NA	NA	NA	2013	2019	1.00	1.00
Frisbie et al., 2022	csPCa	0.43	NA	NA	NA	NA	2018	2020	1.00	0.00
Frisbie et al., 2022	non-csPCa	0.22	NA	NA	NA	NA	2018	2020	1.00	0.00
Wei *et al.*, 2021	csPCa	0.56	NA	NA	0.00	1.00	2015	2020	0.00	1.00
Wei *et al.*, 2021	non-csPCa	0.14	NA	NA	0.00	1.00	2015	2020	0.00	1.00
Maggi *et al.*, 2021	csPCa	0.18	NA	NA	NA	NA	2018	2019	both	both
Maggi *et al.*, 2021	non-csPCa	0.13	NA	NA	NA	NA	2018	2019	both	both

© 2024 Haj-Mirzaian A et al. *JAMA Network Open.*

eTable 5. Baseline characteristics of the included studies: csPCa versus non-csPCa (continued).

		PI-RADS								
	**Category**	**1 or 2**	**3**	**4 or 5**	**4**	**5**	**PNB (ratio)**	**Biopsy naïve (ratio)**	**Prior biopsy (ratio)**	**Gleason 6 baseline (ratio)**
Wang *et al.*, 2021	csPCa	0.06	0.21	0.73	0.24	0.49	0.00	1.00	0.00	0.00
Wang *et al.*, 2021	non-csPCa	0.70	0.21	0.09	0.06	0.03	0.00	1.00	0.00	0.00
Liang et al., 2021	csPCa	1.00	0.00	0.00	0.00	0.00	0.03	0.97	0.03	0.00
Liang et al., 2021	non-csPCa	1.00	0.00	0.00	0.00	0.00	0.09	0.91	0.09	0.00
Fan et al., 2021	csPCa	0.00	0.27	0.00	0.24	0.49	0.15	NA	NA	NA
Fan et al., 2021	non-csPCa	NA	NA	NA	NA	NA	0.38	NA	NA	NA
Apfelbeck et al., 2020	csPCa	0.00	0.00	1.00	0.54	0.46	NA	NA	NA	NA
Apfelbeck et al., 2020	non-csPCa	0.00	0.00	1.00	0.73	0.26	NA	NA	NA	NA
Anastay et al., 2020	csPCa	1.00	0.00	0.00	0.00	0.00	0.20	0.60	0.40	0.20
Anastay et al., 2020	non-csPCa	1.00	0.00	0.00	0.00	0.00	0.19	0.66	0.34	0.15
Kim et al., 2020	csPCa	0.00	0.21	0.79	0.32	0.48	0.24	0.70	0.30	0.06
Kim et al., 2020	non-csPCa	0.00	0.58	0.42	0.34	0.09	0.42	0.54	0.46	0.04
Busetto et al., 2020	csPCa	0.00	0.14	0.86	NA	NA	0.00	1.00	0.00	0.00
Busetto et al., 2020	non-csPCa	0.64	0.22	0.13	NA	NA	0.00	1.00	0.00	0.00
Wei et al., 2020	csPCa	0.14	0.06	0.80	0.57	0.24	0.00	1.00	0.00	0.00
Wei et al., 2020	non-csPCa	0.61	0.21	0.18	0.14	0.04	0.00	1.00	0.00	0.00
Al Hussein Al Awamlh et al., 2020	csPCa	0.00	1.00	0.00	0.00	0.00	0.00	1.00	0.00	0.00
Al Hussein Al Awamlh et al., 2020	non-csPCa	0.00	1.00	0.00	0.00	0.00	0.00	1.00	0.00	0.00
Han et al., 2020	csPCa	0.08	0.11	0.81	0.41	0.41	NA	NA	NA	NA
Han et al., 2020	non-csPCa	0.72	0.16	0.12	0.07	0.05	NA	NA	NA	NA
Borque-Fernando et al., 2019	csPCa	0.05	0.12	0.82	NA	NA	NA	0.59	0.41	NA
Borque-Fernando et al., 2019	non-csPCa	0.31	0.33	0.36	NA	NA	NA	0.41	0.59	NA
Hsieh et al., 2019	csPCa	0.00	0.00	1.00	0.50	0.50	0.21	0.79	0.21	0.00

© 2024 Haj-Mirzaian A et al. *JAMA Network Open.*

Hsieh et al., 2019	non-csPCa	0.45	0.10	0.45	0.33	0.12	0.37	0.63	0.37	0.00
Boesen, Nørgaard, et al., 2019	csPCa	0.07	0.08	0.84	NA	NA	0.00	1.00	0.00	0.00
Boesen, Nørgaard, et al., 2019	non-csPCa	0.53	0.19	0.28	NA	NA	0.00	1.00	0.00	0.00
Boesen, Thomsen, et al., 2019	csPCa	0.05	0.08	0.86	0.26	0.60	0.00	1.00	0.00	0.00
Boesen, Thomsen, et al., 2019	non-csPCa	0.53	0.19	0.28	0.15	0.13	0.00	1.00	0.00	0.00
Sakaguchi et al., 2021	csPCa	0.06	0.27	0.66	0.29	0.38	0.00	1.00	0.00	0.00
Sakaguchi et al., 2021	non-csPCa	0.35	0.27	0.39	0.18	0.21	0.00	1.00	0.00	0.00
Liu et al., 2021	csPCa	1.00	0.00	0.00	0.00	0.00	0.03	0.97	0.03	0.00
Liu et al., 2021	non-csPCa	1.00	0.00	0.00	0.00	0.00	0.13	0.87	0.13	0.00
Zhang et al., 2022	csPCa	1.00	0.00	0.00	0.00	0.00	0.00	1.00	0.00	0.00
Zhang et al., 2022	non-csPCa	1.00	0.00	0.00	0.00	0.00	0.00	1.00	0.00	0.00
Zhou et al., 2022	csPCa	0.00	0.03	0.98	0.47	0.51	0.00	1.00	0.00	0.00
Zhou et al., 2022	non-csPCa	0.00	0.15	0.85	0.67	0.18	0.00	1.00	0.00	0.00
van Riel et al., 2022	csPCa	1.00	0.00	0.00	0.00	0.00	0.13	0.88	0.13	0.00
van Riel et al., 2022	non-csPCa	1.00	0.00	0.00	0.00	0.00	0.20	0.80	0.20	0.00
Wagaskar et al., 2022	csPCa	0.09	0.09	0.82	0.49	0.33	0.06	NA	NA	0.00
Wagaskar et al., 2022	non-csPCa	0.42	0.24	0.32	0.27	0.05	0.35	NA	NA	0.00
Wei et al., 2022	csPCa	0.04	0.06	0.90	NA	NA	NA	NA	NA	NA
Wei et al., 2022	non-csPCa	0.64	0.21	0.15	NA	NA	NA	NA	NA	NA
Frisbie et al., 2022	csPCa	0.02	0.14	0.85	0.54	0.30	NA	NA	NA	0.00
Frisbie et al., 2022	non-csPCa	0.43	0.36	0.21	0.17	0.04	NA	NA	NA	0.00
Wei *et al.*, 2021	csPCa	0.05	0.19	0.76	0.05	0.70	NA	NA	NA	NA
Wei *et al.*, 2021	non-csPCa	0.65	0.26	0.09	0.07	0.03	NA	NA	NA	NA
Maggi *et al.*, 2021	csPCa	0.16	0.23	0.61	NA	NA	0.00	1.00	0.00	0.00
Maggi *et al.*, 2021	non-csPCa	0.68	0.16	0.16	NA	NA	0.00	1.00	0.00	0.00

© 2024 Haj-Mirzaian A et al. *JAMA Network Open.*

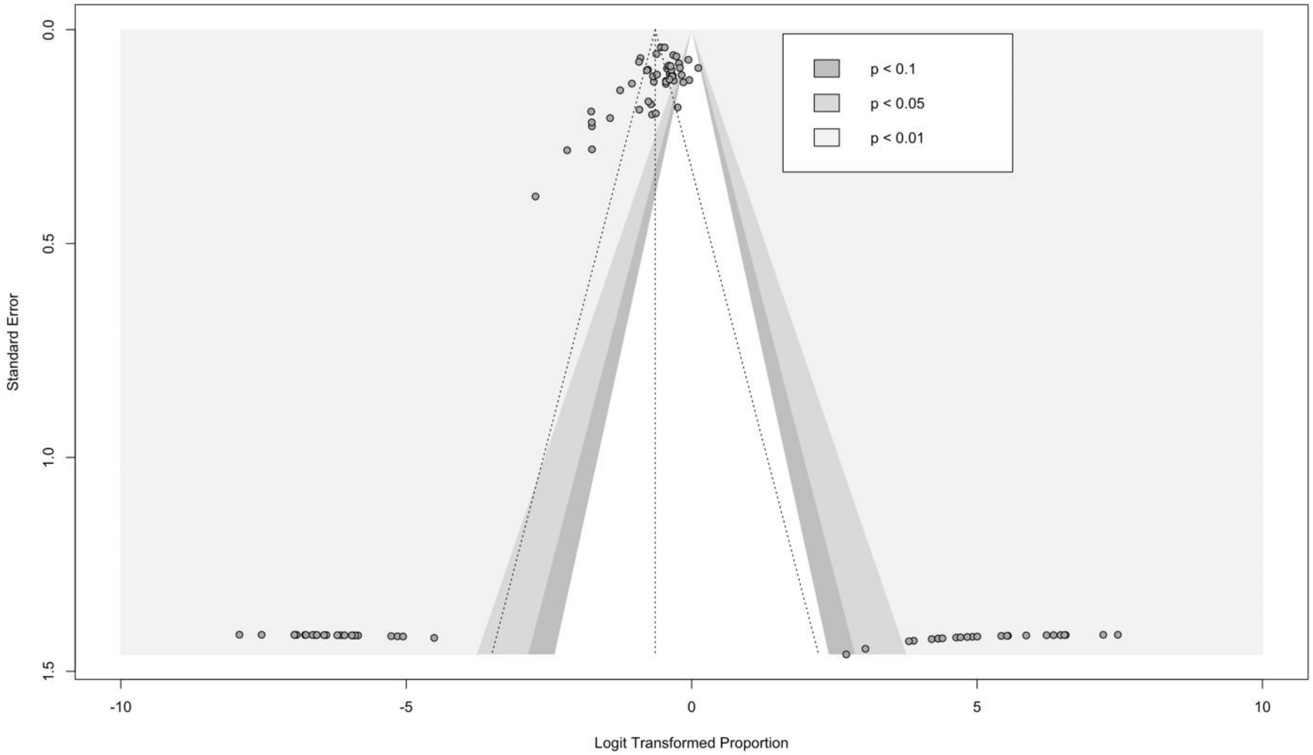

eFigure 2. Funnel Plot. Publication bias was not detected with *P* = 0.651 for the weighted linear regression test.

© 2024 Haj-Mirzaian A et al. *JAMA Network Open.*

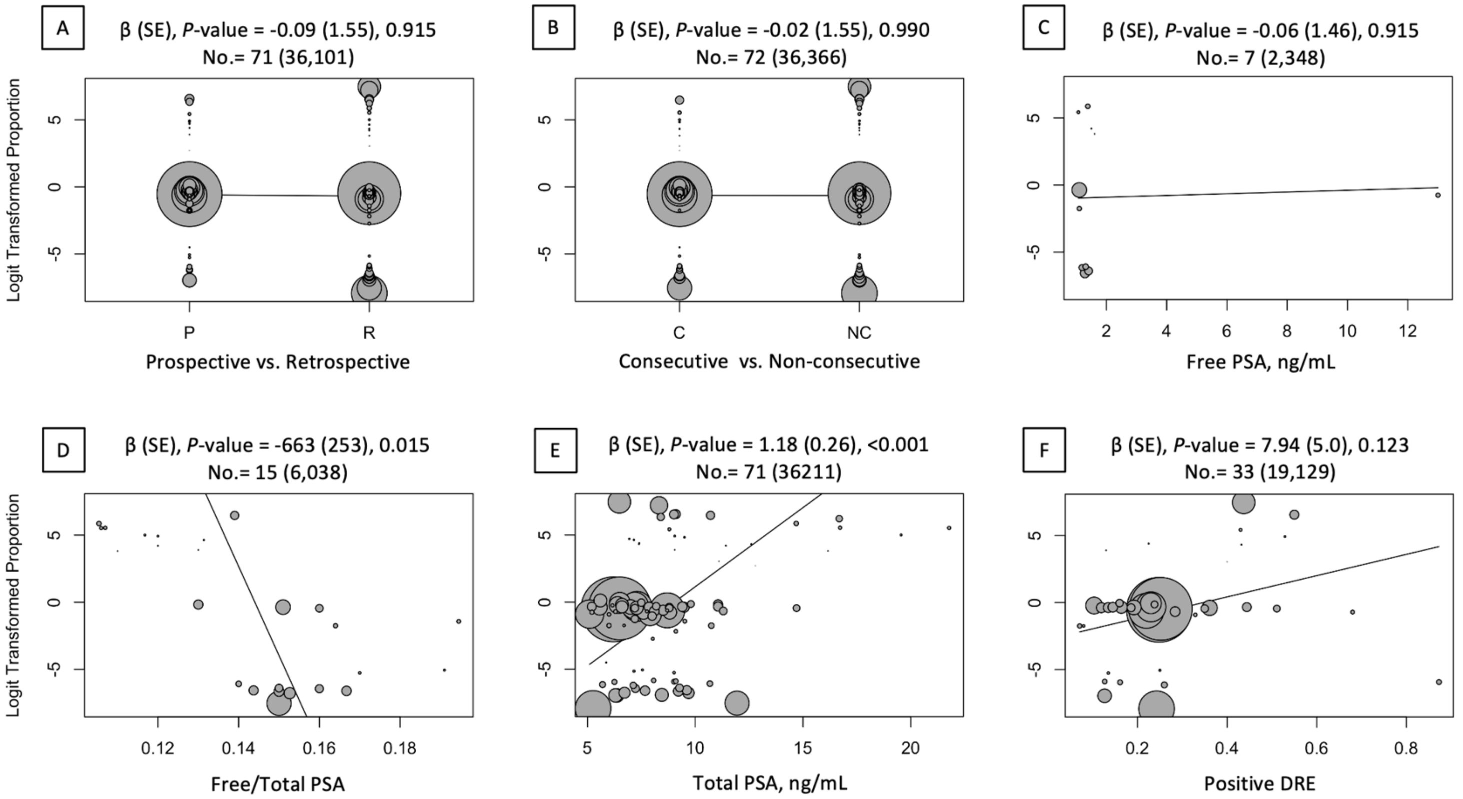

eFigure 3. Univariable Meta-Regression for the Association Between the Rate of csPCa and Clinical Parameters: meta-regression of the proportion of csPCa vs mean/median of parameters on a study-level basis. Values on the y axes represent the logit transformed rate of csPCa effect based on a random-effects model. *P* values were obtained from a univariate linear mixed effects model. The first line of each panel displays the beta-coefficient (β) and standard error (SE) of meta-regression, along with the P-value indicating whether the beta-coefficient differs from zero. In the second line of each panel, the number of studies (No.) is followed by the number of patients (in parentheses) used for meta-regression.

csPCa = clinically significant prostate cancer; PSA = prostate-specific antigen; DRE = Digital Rectal Exam

© 2024 Haj-Mirzaian A et al. *JAMA Network Open.*

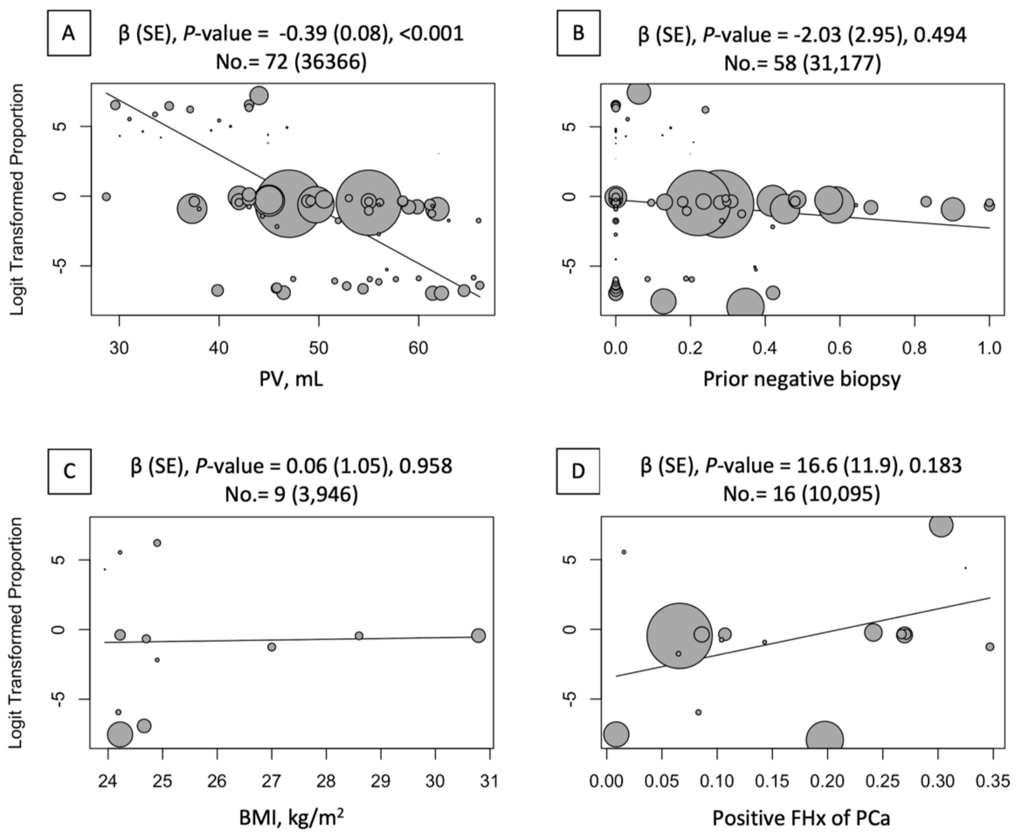

eFigure 4. Univariable Meta-Regression for the Association Between the Rate of csPCa and Prostate Volume/Clinical Parameters: meta-regression of the proportion of csPCa vs mean/median of parameters on a study-level basis. Values on the y axes represent the logit transformed rate of csPCa effect based on a random-effects model. *P* values were obtained from a univariate linear mixed effects model. The first line of each panel displays the beta-coefficient (β) and standard error (SE) of meta-regression, along with the P-value indicating whether the beta-coefficient differs from zero. In the second line of each panel, the number of studies (No.) is followed by the number of patients (in parentheses) used for meta-regression.

csPCa = clinically significant prostate cancer; PV = Prostate volume; BMI = Body Mass Index; FHx = Family history; PCa = prostate cancer

© 2024 Haj-Mirzaian A et al. *JAMA Network Open.*

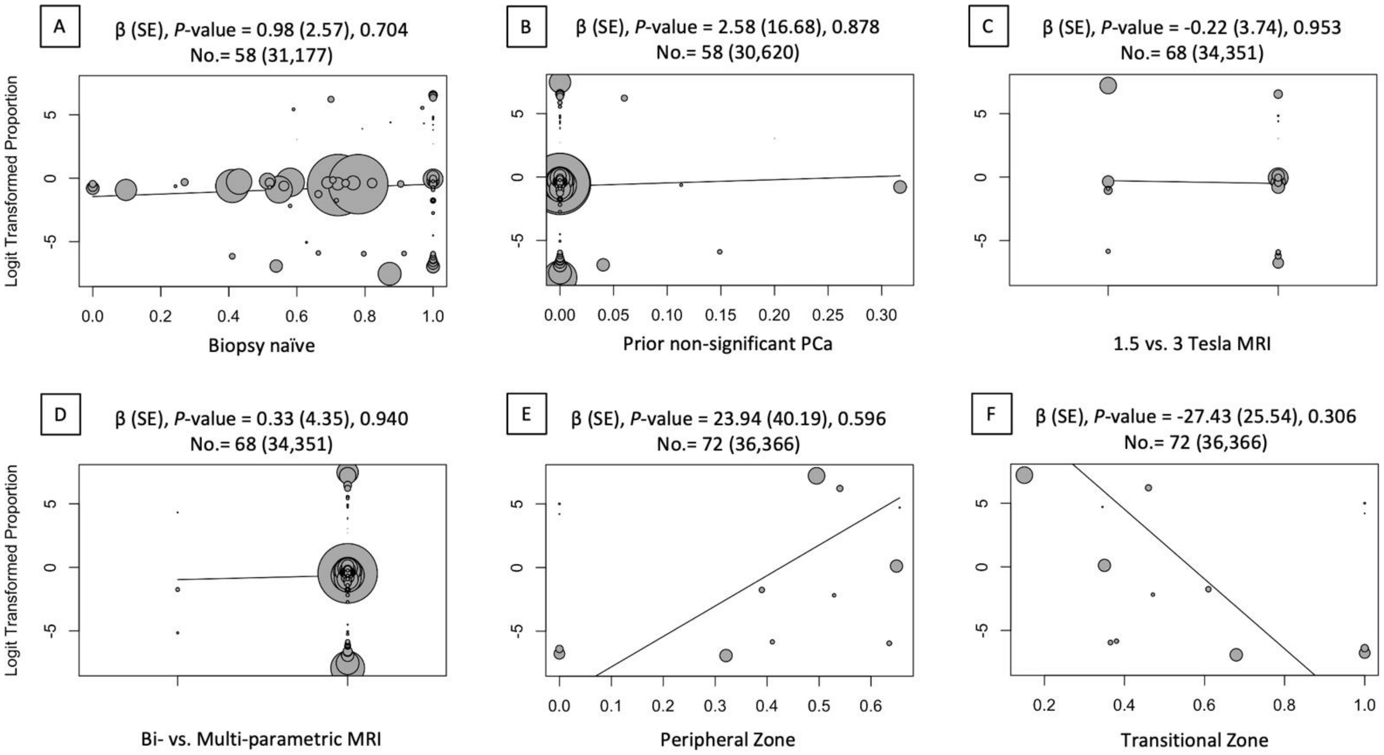

eFigure 5. Univariable Meta-Regression for the Association Between the Rate of csPCa and Clinical/Imaging Parameters: meta-regression of the proportion of csPCa vs mean/median of parameters on a study-level basis. Values on the y axes represent the logit transformed rate of csPCa effect based on a random-effects model. *P* values were obtained from a univariate linear mixed effects model. The first line of each panel displays the beta-coefficient (β) and standard error (SE) of meta-regression, along with the P-value indicating whether the beta-coefficient differs from zero. In the second line of each panel, the number of studies (No.) is followed by the number of patients (in parentheses) used for meta-regression.

csPCa = clinically significant prostate cancer; PCa = prostate cancer; MRI = Magnetic Resonance Imaging

© 2024 Haj-Mirzaian A et al. *JAMA Network Open.*

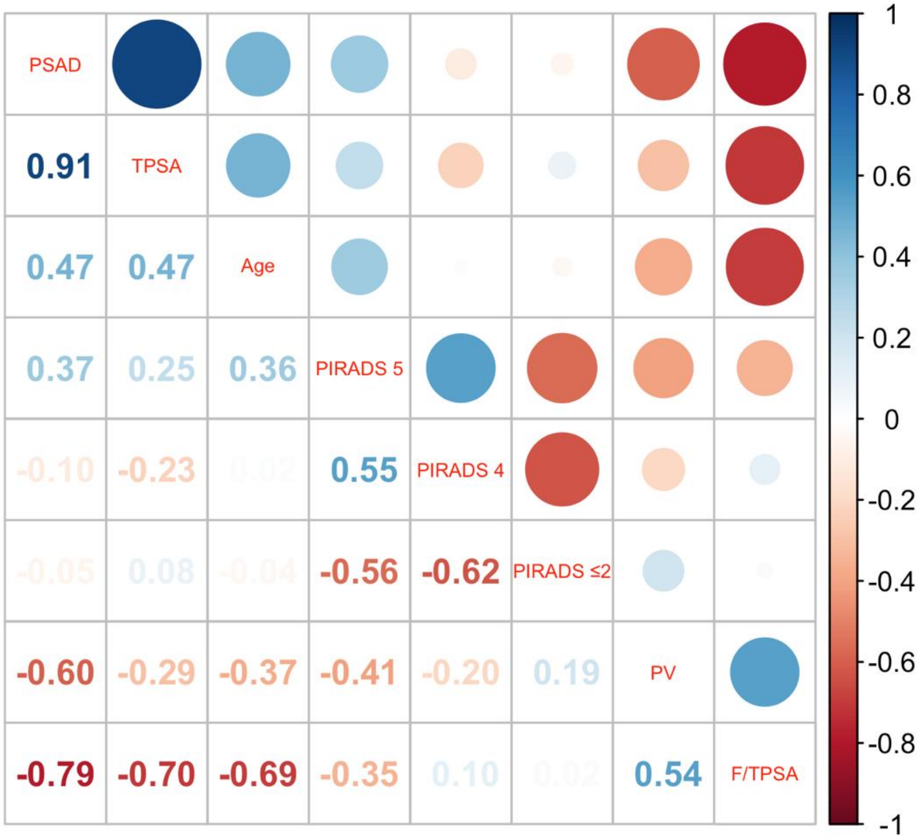

eFigure 6. Intercorrelation Matrix Between Risk Factors of csPCa. Correlation values (r) were represented (range -1 to +1). To avoid multi-collinearity, each multiple variables meta-regression model was built based on a distinct set of non-colinear variables.

csPCa = clinically significant prostate cancer; PSAD = prostate-specific antigen density; TPSA = total prostate-specific antigen; F/TPSA = free to total prostate-specific antigen ratio; PI-RADS = Prostate Imaging Reporting & Data System

© 2024 Haj-Mirzaian A et al. *JAMA Network Open.*

eTable 6. Multiple Variable Meta-Regression on a Subset of Studies: Subgroup Analysis

	Beta coefficient (Standard Error)	Risk factor importance	# *P*-value, comparing each model with the original model including all studies
**A) Studies with no risk of bias**			
Number of studies (patients)	64 (33173)		
**Model 1**			>0.05
Age, y	-0.02 (0.15)	0.63	
PSAD, ng/mL^2	27.8 (5.8)	1.0	
PI-RADS ≤2 or no focal lesion	-2.2 (1.4)	0.92	
PI-RADS 5	9.3 (3.3)	1.0	
**Model 2**			>0.05
Age, y	-0.01 (0.15)	0.64	
PSAD, ng/mL^2	25.4 (5.7)	1.0	
PI-RADS 4	1.7 (2.6)	0.73	
PI-RADS 5	11.0 (3.6)	0.99	
**B) Studies including all suspected patients regardless of the result of prostate MRI or clinical parameters**			
Number of studies (patients)	46 (27423)		
**Model 1**			>0.05
Age, y	0.05 (0.12)	0.56	
PSAD, ng/mL^2	1.3 (5.0)	1.0	
PI-RADS ≤2 or no focal lesion	-9.0 (2.5)	1.0	
PI-RADS 5	9.7 (3.7)	1.0	
**Model 2**			>0.05
Age, y	0.03 (0.12)	0.54	
PSAD, ng/mL^2	5.0 (4.9)	1.0	
PI-RADS 4	9.9 (2.5)	1.0	
PI-RADS 5	14.7 (3.0)	1.0	
**C) Studies only including biopsy-naïve patients**			
Number of studies (patients)	25 (8915)		
**Model 1**			
Age, y	-0.02 (0.19)	0.40	>0.05
PSAD, ng/mL^2	33.0 (10.3)	1.00	
PI-RADS ≤2 or no focal lesion	-3.4 (2.2)	0.87	

© 2024 Haj-Mirzaian A et al. *JAMA Network Open.*

	Beta coefficient (Standard Error)	Risk factor importance	# *P*-value, comparing each model with the original model including all studies
PI-RADS 5	9.0 (4.1)	1.00	
**Model 2**			
Age, y	-0.02 (0.22)	0.46	>0.05
PSAD, ng/mL^2	32.0 (11.6)	1.00	
PI-RADS 4	4.1 (5.2)	0.65	
PI-RADS 5	8.3 (5.8)	0.85	

Multiple variable meta-regression for the association between the rate of csPCa and clinical/imaging parameters in subset of included studies including (A) studies with no risk of bias, (B) studies including all suspected patients regardless of the result of prostate MRI or clinical parameters, and (C) studies only including biopsy-naïve patients: Multiple variables meta-regression of the proportion of csPCa vs. mean/median of continuous clinical/imaging parameters on a study-level basis. To avoid multi-collinearity, each model was built based on a distinct set of non-colinear variables. Multi-model inference model represents variables with the highest importance in estimating csPCa.

Beta-coefficient and standard error of meta-regression are reported. Number of studies followed by the number of patients (in parentheses) used for meta-regression are also presented. For risk factor importance, variables with an importance of 1 represent the highest significance.

#P-value comparing the results of meta-regression between each sub-group versus including all studies.

csPCa = clinically significant prostate cancer; MRI = magnetic resonance imaging; PSAD = prostate-specific antigen density; PI-RADS = Prostate Imaging Reporting & Data System

© 2024 Haj-Mirzaian A et al. *JAMA Network Open.*

eReferences

1. Al Hussein Al Awamlh B, Marks LS, Sonn GA, Natarajan S, Fan RE, Gross MD, et al. Multicenter analysis of clinical and MRI characteristics associated withdetecting clinically significant prostate cancer in PI-RADS (v2.0)category 3 lesions. Urol Oncol. 2020;38(7):637.e9-637.e15.

2. Alberts AR, Roobol MJ, Verbeek JFM, Schoots IG, Chiu PK, Osses DF, et al. Prediction of High-grade Prostate Cancer Following MultiparametricMagnetic Resonance Imaging: Improving the Rotterdam European RandomizedStudy of Screening for Prostate Cancer Risk Calculators. Eur Urol. 2018;75(2):310–8.

3. Anastay V, Gondran-Tellier B, McManus R, Delonca R, Akiki A, Gaillet S, et al. Nonsuspicious prebiopsy multiparametric MRI: is prostate biopsy stillnecessary? Abdom Radiol (NY). 2020;45(12):4160–5.

4. Apfelbeck M, Pfitzinger P, Bischoff R, Rath L, Buchner A, Mumm JN, et al. Predictive clinical features for negative histopathology ofMRI/Ultrasound-fusion-guided prostate biopsy in patients with highlikelihood of cancer at prostate MRI: Analysis from a urologic outpatientclinic1. Clin Hemorheol Microcirc. 2020;76(4):503–11.

5. Bhat NR, Vetter JM, Andriole GL, Shetty AS, Ippolito JE, Kim EH. Magnetic Resonance Imaging-Defined Prostate-Specific Antigen DensitySignificantly Improves the Risk Prediction for Clinically SignificantProstate Cancer on Biopsy. Urology. 2018;126:152–7.

6. Bittencourt LK, Guricova K, Zucker I, Durieux JC, Schoots IG. Risk-based MRI-directed diagnostic pathway outperforms non-risk-basedpathways in suspected prostate cancer biopsy-naïve men: a large cohortvalidation study. Eur Radiol. 2022;32(4):2330–9.

7. Boesen L, Nørgaard N, Løgager V, Balslev I, Bisbjerg R, Thestrup KC, et al. Prebiopsy Biparametric Magnetic Resonance Imaging Combined with Prostate-specific Antigen Density in Detecting and Ruling out Gleason 7-10 Prostate Cancer in Biopsy-naïve Men. Eur Urol Oncol. 2019 May;2(3):311–9.

8. Boesen L, Thomsen FB, Nørgaard N, Løgager V, Balslev I, Bisbjerg R, et al. A predictive model based on biparametric magnetic resonance imaging andclinical parameters for improved risk assessment and selection ofbiopsy-naïve men for prostate biopsies. Prostate Cancer Prostatic Dis. 2019;22(4):609–16.

9. Borque-Fernando Á, Esteban LM, Celma A, Roche S, Planas J, Regis L, et al. How to implement magnetic resonance imaging before prostate biopsy inclinical practice: nomograms for saving biopsies. World J Urol. 2019;38(6):1481–91.

10. Busetto GM, Del Giudice F, Maggi M, De Marco F, Porreca A, Sperduti I, et al. Prospective assessment of two-gene urinary test with multiparametricmagnetic resonance imaging of the prostate for men undergoing primaryprostate biopsy. World J Urol. 2020;39(6):1869–77.

© 2024 Haj-Mirzaian A et al. *JAMA Network Open.*

11. Campistol M, Morote J, Triquell M, Regis L, Celma A, de Torres I, et al. Comparison of Proclarix, PSA Density and MRI-ERSPC Risk Calculator to Select Patients for Prostate Biopsy after mpMRI. Cancers [Internet]. 2022;14(11).

12. Chau EM, Russell B, Santaolalla A, Van Hemelrijck M, McCracken S, Page T, et al. MRI-based nomogram for the prediction of prostate cancer diagnosis: A multi-centre validated patient–physician decision tool. Journal of Clinical Urology [Internet]. Available from: https://www.embase.com/search/results?subaction=viewrecord&id=L2014685902&from=export

13. Cuocolo R, Stanzione A, Rusconi G, Petretta M, Ponsiglione A, Fusco F, et al. PSA-density does not improve bi-parametric prostate MR detection ofprostate cancer in a biopsy naïve patient population. Eur J Radiol. 2018;104:64–70.

14. Deniffel D, Zhang Y, Salinas E, Satkunasivam R, Khalvati F, Haider MA. Reducing Unnecessary Prostate Multiparametric Magnetic Resonance Imaging by Using Clinical Parameters to Predict Negative and Indeterminate Findings. Journal of Urology. 2020;203(2):292–8.

15. Deniffel D, Healy GM, Dong X, Ghai S, Salinas-Miranda E, Fleshner N, et al. Avoiding Unnecessary Biopsy: MRI-based Risk Models versus a PI-RADS andPSA Density Strategy for Clinically Significant Prostate Cancer. Radiology. 2021;300(2):369–79.

16. Ding Z, Wu H, Song D, Tian H, Ye X, Liang W, et al. Development and validation of a nomogram for predicting prostate cancer in men with prostate-specific antigen grey zone based on retrospective analysis of clinical and multi-parameter magnetic resonance imaging/transrectal ultrasound fusion-derived data. Translational Andrology and Urology. 2020;9(5):2179–91.

17. Distler FA, Radtke JP, Bonekamp D, Kesch C, Schlemmer HP, Wieczorek K, et al. The Value of PSA Density in Combination with PI-RADS™ for the Accuracy ofProstate Cancer Prediction. J Urol. 2017;198(3):575–82.

18. Falagario UG, Jambor I, Lantz A, Ettala O, Stabile A, Taimen P, et al. Combined Use of Prostate-specific Antigen Density and Magnetic ResonanceImaging for Prostate Biopsy Decision Planning: A RetrospectiveMulti-institutional Study Using the Prostate Magnetic Resonance ImagingOutcome Database (PROMOD). Eur Urol Oncol. 2020;4(6):971–9.

19. Fan YH, Pan PH, Cheng WM, Wang HK, Shen SH, Liu HT, et al. The Prostate Health Index aids multi-parametric MRI in diagnosingsignificant prostate cancer. Sci Rep. 2021;11(1):1286.

20. Fang D, Zhao C, Ren D, Yu W, Wang R, Wang H, et al. Could Magnetic Resonance Imaging Help to Identify the Presence of ProstateCancer Before Initial Biopsy? The Development of Nomogram Predicting theOutcomes of Prostate Biopsy in the Chinese Population. Ann Surg Oncol. 2016;23(13):4284–92.

© 2024 Haj-Mirzaian A et al. *JAMA Network Open.*

21. Frisbie JW, Van Besien AJ, Lee A, Xu L, Wang S, Choksi A, et al. PSA density is complementary to prostate MP-MRI PI-RADS scoring system for risk stratification of clinically significant prostate cancer. Prostate Cancer and Prostatic Diseases [e-publication May 2022]. Available from: https://www.embase.com/search/results?subaction=viewrecord&id=L2016445852&from=export

22. Gan JM, Kikano EG, Smith DA, Rao S, Podury R, Wang M, et al. Clinically Significant Prostate Cancer Detection after a Negative Prebiopsy MRI Examination: Comparison of Biparametric Versus Multiparametric MRI. American Journal of Roentgenology. 2022;218(5):859–66.

23. Girometti R, Giannarini G, Panebianco V, Maresca S, Cereser L, De Martino M, et al. Comparison of different thresholds of PSA density for risk stratification of PI-RADSv2.1 categories on prostate MRI. British Journal of Radiology [Internet]. 2022;95(1131).

24. Görtz M, Radtke JP, Hatiboglu G, Schütz V, Tosev G, Güttlein M, et al. The Value of Prostate-specific Antigen Density for ProstateImaging-Reporting and Data System 3 Lesions on Multiparametric Magnetic Resonance Imaging: A Strategy to Avoid Unnecessary Prostate Biopsies. Eur Urol Focus. 2019;7(2):325–31.

25. Han C, Liu S, Qin XB, Ma S, Zhu LN, Wang XY. MRI combined with PSA density in detecting clinically significant prostate cancer in patients with PSA serum levels of 4∼10ng/mL: Biparametric versus multiparametric MRI. Diagn Interv Imaging. 2020;101(4):235–44.

26. Hansen NL, Barrett T, Kesch C, Pepdjonovic L, Bonekamp D, O’Sullivan R, et al. Multicentre evaluation of magnetic resonance imaging supported transperineal prostate biopsy in biopsy-naïve men with suspicion of prostate cancer. BJU Int. 2017;122(1):40–9.

27. Hansen NL, Barrett T, Koo B, Doble A, Gnanapragasam V, Warren A, et al. The influence of prostate-specific antigen density on positive and negative predictive values of multiparametric magnetic resonance imaging to detect Gleason score 7-10 prostate cancer in a repeat biopsy setting. BJU Int. 2016;119(5):724–30.

28. He BM, Shi ZK, Li HS, Lin HZ, Yang QS, Lu JP, et al. A Novel Prediction Tool Based on Multiparametric Magnetic Resonance Imaging to Determine the Biopsy Strategy for Clinically Significant Prostate Cancer in Patients with PSA Levels Less than 50 ng/ml. Ann Surg Oncol. 2019;27(4):1284–95.

29. Hogan D, Yao HHI, Kanagarajah A, Ogluszko C, Tran PVP, Dundee P, et al. Can multi-parametric magnetic resonance imaging and prostate-specific antigen density accurately stratify patients prior to prostate biopsy? Journal of Clinical Urology [Internet]. Available from: https://www.embase.com/search/results?subaction=viewrecord&id=L2015619735&from=export

30. Hsieh PF, Li WJ, Lin WC, Chang H, Chang CH, Huang CP, et al. Combining prostate health index and multiparametric magnetic resonance imaging in the diagnosis of clinically significant prostate cancer in an Asian population. World J Urol. 2019;38(5):1207–14.

© 2024 Haj-Mirzaian A et al. *JAMA Network Open.*

31. Huang C, Song G, Wang H, Ji G, Li J, Chen Y, et al. MultiParametric Magnetic Resonance Imaging-Based Nomogram for Predicting Prostate Cancer and Clinically Significant Prostate Cancer in Men Undergoing Repeat Prostate Biopsy. Biomed Res Int. 2018;2018:6368309.

32. Keck B, Borkowetz A, Poellmann J, Jansen T, Fischer M, Fuessel S, et al. Serum miRNAs Support the Indication for MRI-Ultrasound Fusion-Guided Biopsy of the Prostate in Patients with Low-PI-RADS Lesions. Cells [Internet]. 2021;10(6).

33. Kim M, Ryu H, Lee HJ, Hwang SI, Choe G, Hong SK. Who can safely evade a magnetic resonance imaging fusion-targeted biopsy(MRIFTB) for prostate imaging reporting and data system (PI-RADS) 3lesion? World J Urol. 2020;39(5):1463–71.

34. Kim TJ, Lee MS, Hwang SI, Lee HJ, Hong SK. Outcomes of magnetic resonance imaging fusion-targeted biopsy of prostate imaging reporting and data system 3 lesions. World J Urol. 2018;37(8):1581–6.

35. Lee CM, Park KJ, Kim MH, Kim JK. Ancillary imaging and clinical features for the characterization of prostate lesions: A proposed approach to reduce false positives. J Magn Reson Imaging. 2020;53(6):1887–97.

36. Lendínez-Cano G, Ojeda-Claro AV, Gómez-Gómez E, Morales Jimenez P, Flores Martin J, Dominguez JF, et al. Prospective study of diagnostic accuracy in the detection of high-grade prostate cancer in biopsy-naïve patients with clinical suspicion of prostate cancer who underwent the Select MDx test. Prostate. 2021;81(12):857–65.

37. Liang L, Qi F, Cheng Y, Zhang L, Cao D, Cheng G, et al. Analysis of risk factors for determining the need for prostate biopsy inpatients with negative MRI. Sci Rep. 2021;11(1):6048.

38. Lim C, Abreu-Gomez J, Leblond MA, Carrion I, Vesprini D, Schieda N, et al. When to biopsy prostate imaging and data reporting system version 2 (PI-RADSv2) assessment category 3 lesions? Use of clinical and imaging variables to predict cancer diagnosis at targeted biopsy. Canadian Urological Association Journal [Internet]. 2020;15(4).

39. Liu G, Zhu Y, Yao Z, Jiang Y, Wu B, Bai S. Development and validation of a predictive model for determining clinically significant prostate cancer in men with negative magnetic resonance imaging after transrectal ultrasound-guided prostate biopsy. Prostate. 2021;81(13):983–91.

40. Lu YF, Zhang Q, Chen HY, Chen JY, Pan Y, Xu CC, et al. Improving the detection rate of prostate cancer in the gray zone of PI-RADS v2 and serum tPSA by using prostate-specific antigen-age volume. Medicine (Baltimore). 2019;98(26):e16289.

41. Lu YF, Zhang Q, Yao WG, Chen HY, Chen JY, Xu CC, et al. Optimizing prostate cancer accumulating model: combined PI-RADS v2 with prostate specific antigen and its derivative data. Cancer Imaging. 2019;19(1):26.

© 2024 Haj-Mirzaian A et al. *JAMA Network Open.*

42. Maggi M, Del Giudice F, Falagario UG, Cocci A, Russo GI, Di Mauro M, et al. Select mdx and multiparametric magnetic resonance imaging of the prostate for men undergoing primary prostate biopsy: A prospective assessment in a multi-institutional study. Cancers [Internet]. 2021;13(9).

43. Mehralivand S, Shih JH, Rais-Bahrami S, Oto A, Bednarova S, Nix JW, et al. A Magnetic Resonance Imaging-Based Prediction Model for Prostate Biopsy Risk Stratification. JAMA Oncol. 2018;4(5):678–85.

44. Morote J, Borque-Fernando A, Triquell M, Celma A, Regis L, Mast R, et al. Comparative Analysis of PSA Density and an MRI-Based Predictive Model to Improve the Selection of Candidates for Prostate Biopsy. Cancers [Internet]. 2022;14(10).

45. Morote J, Campistol M, Triquell M, Celma A, Regis L, de Torres I, et al. Improving the Early Detection of Clinically Significant Prostate Cancer in Men in the Challenging Prostate Imaging-Reporting and Data System 3 Category. European Urology Open Science. 2022;37:38–44.

46. Morote J, Schwartzman I, Borque A, Esteban LM, Celma A, Roche S, et al. Prediction of clinically significant prostate cancer after negative prostate biopsy: The current value of microscopic findings. Urol Oncol. 2020;39(7):432.e11-432.e19.

47. Niu XK, He WF, Zhang Y, Das SK, Li J, Xiong Y, et al. Developing a new PI-RADS v2-based nomogram for forecasting high-grade prostate cancer. Clin Radiol. 2017;72(6):458–64.

48. Niu XK, Li J, Das SK, Xiong Y, Yang CB, Peng T. Developing a nomogram based on multiparametric magnetic resonance imaging for forecasting high-grade prostate cancer to reduce unnecessary biopsies within the prostate-specific antigen gray zone. BMC Med Imaging. 2017;17(1):11.

49. Noh TI, Hyun CW, Kang HE, Jin HJ, Tae JH, Shim JS, et al. A Predictive Model Based on Bi-parametric Magnetic Resonance Imaging and Clinical Parameters for Clinically Significant Prostate Cancer in the Korean Population. Cancer Res Treat. 2020;53(4):1148–55.

50. Oishi M, Shin T, Ohe C, Nassiri N, Palmer SL, Aron M, et al. Which Patients with Negative Magnetic Resonance Imaging Can Safely Avoid Biopsy for Prostate Cancer? J Urol. 2019;201(2):268–76.

51. Pan JF, Su R, Cao JZ, Zhao ZY, Ren DW, Ye SZ, et al. Modified Predictive Model and Nomogram by Incorporating Prebiopsy Biparametric Magnetic Resonance Imaging With Clinical Indicators for Prostate Biopsy Decision Making. Frontiers in Oncology [Internet]. Available from: https://www.embase.com/search/results?subaction=viewrecord&id=L636073522&from=export

52. Petersmann AL, Remmers S, Klein T, Manava P, Huettenbrink C, Pahernik SA, et al. External validation of two MRI-based risk calculators in prostate cancer diagnosis. World J Urol. 2021;39(11):4109–16.

© 2024 Haj-Mirzaian A et al. *JAMA Network Open.*

53. Polanec SH, Bickel H, Wengert GJ, Arnoldner M, Clauser P, Susani M, et al. Can the addition of clinical information improve the accuracy of PI-RADSversion 2 for the diagnosis of clinically significant prostate cancer in positive MRI? Clin Radiol. 2019;75(2):157.e1-157.e7.

54. Püllen L, Radtke JP, Wiesenfarth M, Roobol MJ, Verbeek JFM, Wetter A, et al. External validation of novel magnetic resonance imaging-based models for prostate cancer prediction. BJU Int. 2019;125(3):407–16.

55. Punnen S, Nahar B, Soodana-Prakash N, Koru-Sengul T, Stoyanova R, Pollack A, et al. Optimizing patient’s selection for prostate biopsy: A single institution experience with multi-parametric MRI and the 4Kscore test for the detection of aggressive prostate cancer. PLoS One. 2018;13(8):e0201384.

56. Radtke JP, Giganti F, Wiesenfarth M, Stabile A, Marenco J, Orczyk C, et al. Prediction of significant prostate cancer in biopsy-naïve men: Validationof a novel risk model combining MRI and clinical parameters and comparison to an ERSPC risk calculator and PI-RADS. PLoS One. 2019;14(8):e0221350.

57. Radtke JP, Wiesenfarth M, Kesch C, Freitag MT, Alt CD, Celik K, et al. Combined Clinical Parameters and Multiparametric Magnetic Resonance Imaging for Advanced Risk Modeling of Prostate Cancer-Patient-tailored Risk Stratification Can Reduce Unnecessary Biopsies. Eur Urol. 2017;72(6):888–96.

58. Ryoo H, Kang MY, Sung HH, Chang Jeong B, Seo SI, Jeon SS, et al. Detection of prostate cancer using prostate imaging reporting and data system score and prostate-specific antigen density in biopsy-naive and prior biopsy-negative patients. Prostate International. 2020;8(3):125–9.

59. Saba K, Wettstein MS, Lieger L, Hötker AM, Donati OF, Moch H, et al. External Validation and Comparison of Prostate Cancer Risk Calculators Incorporating Multiparametric Magnetic Resonance Imaging for Prediction of Clinically Significant Prostate Cancer. J Urol. 2019;203(4):719–26.

60. Sakaguchi K, Hayashida M, Tanaka N, Oka S, Urakami S. A risk model for detecting clinically significant prostate cancer based onbi-parametric magnetic resonance imaging in a Japanese cohort. Sci Rep. 2021;11(1):18829.

61. Sokhi HK, Padhani AR, Patel S, Pope A. Diagnostic yields in patients with suspected prostate cancer undergoing MRI as the first-line investigation in routine practice. Clin Radiol. 2020;75(12):950–6.

62. Sonmez G, Demirtas T, Tombul ST, Akgun H, Demirtas A. Diagnostic efficiency of systemic immune-inflammation index in fusion prostate biopsy. Actas Urol Esp (Engl Ed). 2021;45(5):359–65.

63. Sonmez G, Tombul ST, Demirtas T, Demirtas A. Clinical factors for predicting malignancy in patients with PSA < 10 ng/mLand PI-RADS 3 lesions. Asia Pac J Clin Oncol. 2020;17(2):e94–9.

© 2024 Haj-Mirzaian A et al. *JAMA Network Open.*

64. Stevens E, Truong M, Bullen JA, Ward RD, Purysko AS, Klein EA. Clinical utility of PSAD combined with PI-RADS category for the detection of clinically significant prostate cancer. Urol Oncol. 2020 Nov;38(11):846.e9-846.e16.

65. Tan TW, Png KS, Lee CH, Yuwono A, Yeow Y, Chong KT, et al. MRI Fusion-Targeted Transrectal Prostate Biopsy and the Role of Prostate-Specific Antigen Density and Prostate Health Index for the Detection of Clinically Significant Prostate Cancer in Southeast Asian Men. J Endourol. 2017;31(11):1111–6.

66. Thompson JE, van Leeuwen PJ, Moses D, Shnier R, Brenner P, Delprado W, et al. The Diagnostic Performance of Multiparametric Magnetic Resonance Imaging to Detect Significant Prostate Cancer. J Urol. 2015;195(5):1428–35.

67. Tosun M, Uslu H. Prebiopsy multiparametric MRI and PI-RADS version 2.0 for differentiating histologically benign prostate disease from prostate cancer in biopsies: A retrospective single-center comparison. Clin Imaging. 2021;78:98–103.

68. Truong M, Wang B, Gordetsky JB, Nix JW, Frye TP, Messing EM, et al. Multi-institutional nomogram predicting benign prostate pathology on magnetic resonance/ultrasound fusion biopsy in men with a prior negative12-core systematic biopsy. Cancer. 2017;124(2):278–85.

69. van Leeuwen PJ, Hayen A, Thompson JE, Moses D, Shnier R, Böhm M, et al. A multiparametric magnetic resonance imaging-based risk model to determine the risk of significant prostate cancer prior to biopsy. BJU Int. 2017;120(6):774–81.

70. van Riel LAMJG, Jager A, Meijer D, Postema AW, Smit RS, Vis AN, et al. Predictors of clinically significant prostate cancer in biopsy-naïve and prior negative biopsy men with a negative prostate MRI: improving MRI-based screening with a novel risk calculator. Therapeutic Advances in Urology [Internet]. 2022;14. Available from: https://www.embase.com/search/results?subaction=viewrecord&id=L2015414117&from=export

71. Wagaskar VG, Lantz A, Sobotka S, Ratnani P, Parekh S, Falagario UG, et al. Development and External Validation of a Prediction Model to Identify Candidates for Prostate Biopsy. Urology journal [Internet]. Available from: https://www.embase.com/search/results?subaction=viewrecord&id=L636978104&from=export

72. Wang ZB, Wei CG, Zhang YY, Pan P, Dai GC, Tu J, et al. The Role of PSA Density among PI-RADS v2.1 Categories to Avoid an Unnecessary Transition Zone Biopsy in Patients with PSA 4-20 ng/mL. Biomed Res Int. 2021;2021:3995789.

73. Washino S, Okochi T, Saito K, Konishi T, Hirai M, Kobayashi Y, et al. Combination of prostate imaging reporting and data system (PI-RADS) score and prostate-specific antigen (PSA) density predicts biopsy outcome in prostate biopsy naïve patients. BJU Int. 2016;119(2):225–33.

74. Wei C, Pan P, Chen T, Zhang Y, Dai G, Tu J, et al. A nomogram based on PI-RADS v2.1 and clinical indicators for predicting clinically significant prostate cancer in the transition zone. Translational Andrology and Urology. 2021;10(6):2435–46.

© 2024 Haj-Mirzaian A et al. *JAMA Network Open.*

75. Wei CG, Chen T, Zhang YY, Pan P, Dai GC, Yu HC, et al. Biparametric prostate MRI and clinical indicators predict clinically significant prostate cancer in men with “gray zone” PSA levels. Eur J Radiol. 2020;127:108977.

76. Wei X, Xu J, Zhong S, Zou J, Cheng Z, Ding Z, et al. Diagnostic value of combining PI-RADS v2.1 with PSAD in clinically significant prostate cancer. Abdominal Radiology [Internet]. Available from: https://www.embase.com/search/results?subaction=viewrecord&id=L2018145048&from=export

77. Záleský M, Stejskal J, Adamcova V, Hrbáček J, Minarik I, Pavlicko A, et al. Use of Prostate Specific Antigen Density Combined with Multiparametric Magnetic Resonance Imaging Improves Triage for Prostate Biopsy. Urol Int. 2019;103(1):33–40.

78. Zhang CC, Tu X, Lin TH, Cai DM, Yang L, Nie L, et al. The role of prostate-specific antigen density and negative multiparametric magnetic resonance imaging in excluding prostate cancer for biopsy-naïve men: clinical outcomes from a high-volume center in China. Asian journal of andrology [Internet]. Available from: https://www.embase.com/search/results?subaction=viewrecord&id=L637956924&from=export

79. Zhang Y, Zeng N, Zhang FB, Rui Huang YX, Tian Y. Performing Precise Biopsy in Naive Patients With Equivocal PI-RADS, Version 2, Score 3, Lesions: An MRI-based Nomogram to Avoid Unnecessary Surgical Intervention. Clin Genitourin Cancer. 2019;18(5):367–77.

80. Zhou Z, Liang Z, Zuo Y, Zhou Y, Yan W, Wu X, et al. Development of a nomogram combining multiparametric magnetic resonance imaging and PSA-related parameters to enhance the detection of clinically significant cancer across different region. Prostate. 2022;82(5):556–65.

81. Zhu H, Ding XF, Lu SM, Ding N, Pi SY, Liu Z, et al. The Application of Biopsy Density in Transperineal Templated-Guided Biopsy Patients With PI-RADS<3. Frontiers in Oncology [Internet]. Available from: https://www.embase.com/search/results?subaction=viewrecord&id=L2017978521&from=export

© 2024 Haj-Mirzaian A et al. *JAMA Network Open.*
