# Supplementary material for: Magnetic Resonance Imaging, Clinical, and Biopsy Findings in Suspected Prostate Cancer: A Systematic Review and Meta-Analysis
Source: JAMA Netw Open. 2024 Mar 29;7(3):e244258. doi: 10.1001/jamanetworkopen.2024.4258 (PMC10980971; doi:10.1001/jamanetworkopen.2024.4258)
Supplement: Supplement 2. — Data Sharing Statement [file jamanetwopen-e244258-s002.pdf]

Data Sharing Statement

Haj-Mirzaian. Magnetic Resonance Imaging, Clinical, and Biopsy Findings in Suspected Prostate Cancer. *JAMA Netw Open*. Published March 29, 2024.
doi:10.1001/jamanetworkopen.2024.4258

Data
Data available: No

Additional Information
Explanation for why data not available: No individual patient data in this meta analysis
